# The Need for Community Standards to Enable Accurate Comparison of Glycoproteomics Algorithm Performance

**DOI:** 10.3390/molecules26164757

**Published:** 2021-08-06

**Authors:** William E. Hackett, Joseph Zaia

**Affiliations:** 1 Bioinformatics Program, Boston University, Boston, MA 02215, USA; wehacket@bu.edu; 2 Department of Biochemistry, School of Medicine, Boston University, Boston, MA 02215, USA

**Keywords:** glycoproteomics, false-discovery rate, standards, target-decoy analysis

## Abstract

Protein glycosylation that mediates interactions among viral proteins, host receptors, and immune molecules is an important consideration for predicting viral antigenicity. Viral spike proteins, the proteins responsible for host cell invasion, are especially important to be examined. However, there is a lack of consensus within the field of glycoproteomics regarding identification strategy and false discovery rate (FDR) calculation that impedes our examinations. As a case study in the overlap between software, here as a case study, we examine recently published SARS-CoV-2 glycoprotein datasets with four glycoproteomics identification software with their recommended protocols: GlycReSoft, Byonic, pGlyco2, and MSFragger-Glyco. These software use different Target-Decoy Analysis (TDA) forms to estimate FDR and have different database-oriented search methods with varying degrees of quantification capabilities. Instead of an ideal overlap between software, we observed different sets of identifications with the intersection. When clustering by glycopeptide identifications, we see higher degrees of relatedness within software than within glycosites. Taking the consensus between results yields a conservative and non-informative conclusion as we lose identifications in the desire for caution; these non-consensus identifications are often lower abundance and, therefore, more susceptible to nuanced changes. We conclude that present glycoproteomics softwares are not directly comparable, and that methods are needed to assess their overall results and FDR estimation performance. Once such tools are developed, it will be possible to improve FDR methods and quantify complex glycoproteomes with acceptable confidence, rather than potentially misleading broad strokes.

## 1. Introduction

In order to properly assess the biological roles of glycosylation, there needs to be a set of consistent standards and assessments for false discovery rate (FDR) methods and identification schema. The field of glycoproteomics is undergoing exciting developments and expansions, to the point that many researchers are reporting site-specific glycosylation for complex glycoproteins, including the spike protein of SARS-CoV-2 [1,2,3,4,5]. Glycoproteomics data will inform understanding of virus infection mechanisms and their evolution over time; however, as more investigators employ glycoproteomics measurements, it is increasingly important to have a firm consensus of best practices for acquiring and analyzing glycoproteomics data. 

To quantify glycoprotein glycosylation and reach confident conclusions regarding the changes that occur in a biological system, all experimental assumptions that influence the calculation of FDRs must be made clear. Glycoproteomic FDR methods are largely developed from those used in proteomics, specifically target decoy analysis (TDA) [6]. TDA is based on scoring a spectrum against a database of potential peptides and decoys, typically derived from the reversed protein sequences; these scores are then ranked, a threshold set, and the number of false positives is correlated to the proportion of decoys to targets scoring above the threshold. 

This process relies on three assumptions: (1) there is a set of reliably false decoys, (2) these false decoys largely mimic scoring of targets for the unidentifiable region of the scoring distribution, and (3) the decoy database is large enough to avoid sampling bias. To date, different glycoproteomic softwares have taken divergent approaches to calculate TDA with the result that the meaning of FDR depends on the assumptions behind each algorithm.

The four software’s tested here (Byonic, GlycReSoft, MSFragger-Glyco, and pGlyco2) use TDA-based methods for calculating FDR. Each has potential benefits and detractors to be considered.

Byonic [7] uses a reverse protein decoy model but does not employ a glycan decoy. In the reverse protein decoy model, peptides are well controlled, but the attached glycans are largely score-dependent features with little direct false positive control. 

GlycReSoft [8], as used here, uses a reverse protein decoy model with mass shifted glycans. Mass shifted glycans have had their mass shifted arbitrarily by 1 to 30 Da; this creates randomness to affect scoring but is not ideal. GlycReSoft performs better using its multi-part search and permuted glycan method, but these methods were not used here due to a data compatibility issue. In the mass-shifted glycan method, both peptide and glycans are controlled for in the FDR method, but glycans are less reliably accounted for than the peptides. 

MSFragger-Glyco [9] uses the reverse protein decoy model and the extended mass model from Peptide-Prophet [10] to treat glycans as PTMs. The Peptide-Prophet extended mass model is a filtering step that calculates the probability of a match based on the difference between the precursor mass and the calculated match mass. Glycans are not explicitly controlled in this system, but peptides are highly controlled.

pGlyco2 [11] uses an aggregate system of FDRs from permuted glycans and reversed peptide searches contributing to the glycopeptide FDR. It tabulates a glycan FDR, a peptide FDR, and then a glycopeptide FDR, which informatively uses the glycan and peptide FDR. This makes both portions of the glycopeptide well-controlled for in FDR. Still, it does make data with poorer fragmentation of either glycan or peptide more susceptible to false-negative results.

These software’s differ in several other significant ways, but each software should find the same glycopeptides as the others in a perfect system. This is false as different scoring algorithms and different FDR methods will lead to divergent findings. But there should be a common ground that all software’s can agree upon. At the least, there should be a strong degree of overlap between their outputs as the most prevalent and clearest glycopeptides rise above the rest in each system.

## 2. Results

Assignment of site-specific glycosylation in SARS-CoV-2 spike protein requires careful consideration of the effects of glycopeptide search space selection, post-translational modification inclusion, protein recombination vectors, software selection, and mass spectral tolerances. We sought to address whether, in the absence of consensus FDR standards for quantitative glycopeptidomic data, the different software programs reach similar glycoproteomics assignments. To better understand the differences in glycoproteomics assignment approaches, we re-analyzed two publicly available datasets: one from the Crispin group at the University of Southampton and the other from the Yang group of Sichuan University. The Crispin dataset was produced from a genetic construct corresponding to the complete spike protein; this data is herein referred to as the Watanabe et al. data. The Yang group analyzed a recombinant S1 subunit of the spike protein; this data is referred to as the Zhang et al. data. Both groups used HEK-293 cell lines for protein expression. 

We compared results for these data sets using Byonic, GlycReSoft, MSFragger-Glyco, and pGlyco2. The original intent of this study was to use the exact same glycopeptide search space for each software with the same PTMs at the same FDR with the same error tolerances. The original intended search-space was a combinatorial search space of viable human glycans with the possibility of sulfated and phosphorylated glycans; sulfated glycans were to be included after their observed presence in a more stringent reanalysis of prior studies and confirmation by another group [2,12].

Unfortunately, the softwares are unable to all use the same glycopeptide search space. Some of the softwares did not allow for custom libraries, and others performed too erroneously in expanded libraries to produce intelligible results even when not considering sulfated glycans. While pGlyco2 has a robust and capable scoring algorithm, it does not allow for sulfated glycans and lacks a library generation system. MSFragger-Glyco can be easily set up to search for sulfated glycans. Still, these searches ran into a problem of interpretation wherein the mass offset system could not distinguish between sulfated glycans and similarly massed glycans on mass alone. Mass offset fails because a glycan with sulfation attached to a HexNAc is less than a twentieth of a Dalton from the same glycan with three Hex instead [13].

Instead of creating an unbalanced comparison where one software prevailed over the others, each software was allowed to use its recommended protocol and search space to assume that these would be the most optimal conditions. The protocols used were those found on the respective softwares’ papers and user manuals with the following adjustments. We assumed the protein search space to consist only of the mutated spike proteins as presented in the repositories; we set error tolerances to 10 ppm for the precursor match and 20 ppm for the fragment match where possible. Oxidation, carboxymethylation, and deamidation were used as PTMs in all searches.

The following results are not meant to show that any software finds more glycopeptides than another, nor is it intended to provide insight into the best software to use. They are presented as a case study in the differing results that arise following the recommended protocols for each software.

### 2.1. Watanabe et al. Reanalysis

In “Site-specific glycan analysis of the SARS-CoV-2 spike” [1], Watanabe et al. used long liquid chromatography gradients (over four hours) compared to most quantitative glycoproteomics experiments (1.5–2 h) and three different proteases in separate experiments, each with a single replicate. They used an automated search and a degree of manual curation to identify glycans at all 22 *N*-glycosylation sequons on the spike protein. The 109 glycans identified were split up by degree of processing into three groups: Oligomannose, Hybrid, and Complex. Oligomannose was composed of glycans with only two HexNAc and four to nine Hexose; hybrid consisted of 8 glycans with low numbers of HexNAc and relatively minimal processing of the Hex branches; complex composed the remaining most processed 95 glycans. They reported that 14 of the sites were predominantly highly processed complex glycans, and eight sites were predominantly minimally processed oligomannose glycans. They contrast this with the oligomannose shielding observed in Lassa, HIV, Influenza, and MERS, and conclude that the SARS-CoV-2 spike glycoprotein is less shielding than in viruses, including HIV and Lassa. They propose that evolutionary pressure drives the formation of glycan shields against host antibodies and find the weak glycan shield observed for SARS-CoV-2 to be fairly consistent with other coronavirus glycosylation.

For the Watanabe et al. data, we used default glycan search spaces for each program, and the peptide search spaces were all given the same fasta file consisting of the SARS-CoV-2 mutated spike protein. This produced a wide variety of search space sizes: Byonic’s 182 human *N*-glycan space with 165 glycans; GlycReSoft’s combinatorial search space with sulfation and phosphorylated glycans for 3888 glycans; MSFragger’s default glycan list for 182 glycans; pGlyco2’s comprehensive default database of 1670 glycans. The proteomic search spaces all used their respective digestion enzymes of trypsin, chymotrypsin, and alpha-lytic protease. 

Due to several glycans not existing within the Watanabe classification chart, we used the system of Zhang et al. [5], which is classified by the number of HexNAc, where those with two are Simple, three are Hybrid, and four or more are Complex. Glycans with less than two HexNAc (included by Byonic, MSFragger, pGlyco2) or glycans that have been sulfated or phosphorylated (included using GlycReSoft) are classified as Other (see Figure S2). Compared to the Watanabe system, this creates a higher number of Simple and Hybrid glycans. A comparison of the proportion of the softwares’ search spaces by classification shows that all softwares have a majority of Complex glycopeptides, except GlycReSoft. Due to the inclusion of sulfated and phosphorylated glycans, GlycReSoft has a much larger proportion of Other-type glycans. Still, if these other-type glycans were reclassified using only the HexNAc criterion, it would also be a majority Complex-type. A graphical depiction can be found in the supplemental information (Figure S2). 

**Figure 1:**
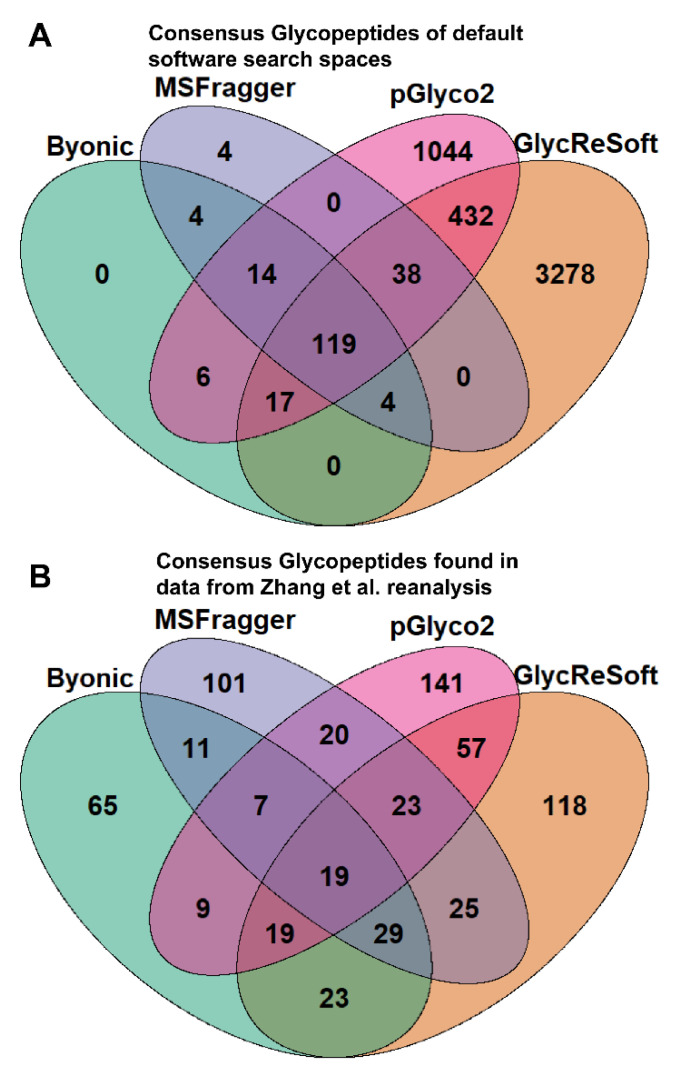
Venn diagrams of consensus between softwares. (**A**) Comparison of the distribution of glycan classes in the glycomics search space used for each glycoproteomics software program tested. (**B**) Consensus glycopeptides found in data from reanalysis of Zhang et al. data.

To better illustrate the overlap between software search spaces, we generated Venn diagrams that show the consensus between glycopeptides in Figure 1A. MSFragger-Glyco and Byonic have few glycopeptides not searched for in other software, but pGlyco2 and GlycReSoft both have a high degree of unique glycans, largely stemming from their inclusion of NeuGc and of sulfated or phosphorylated glycans, respectively. 

We used the result generated by each software without subjective interpretation. Each digest’s results were combined for each software to identify as many glycopeptides as possible (Figure S3b). Byonic observed 14 of the 22 sites (Figure S5a), but there were three pairs of sites where at least one site was indistinguishable from the other due to proximity and peptide overlap for a total of 9 identifiable glycosylation sites. GlycReSoft saw 17 of the 22 sites (Figure S5b) but also had three pairs of overlapping sites for a total of 14 identifiable glycosylation sites. MSFragger observed 16 of the 22 sites (Figure S5c). pGlyco2 was ultimately excluded from the Watanabe et al. reanalysis presentation as it only identified one site, which we suspect is due to the limited search space and FDR method of pGlyco2.

**Figure 2:**
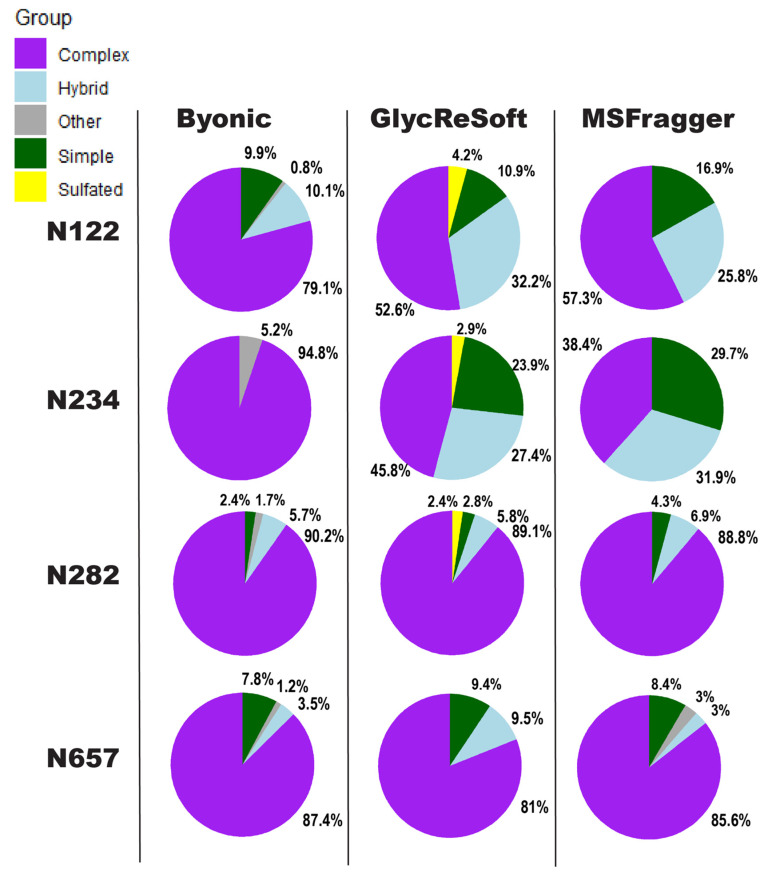
Comparison of results for searching the Watanabe et al. 2020 data using Byonic, GlycReSoft, and MSFragger. Searches were conducted using the recommended settings for each software. The pie charts show the degree of *N*-glycan biosynthetic processing, with sulfated glycans included as “other”. The sum of relative log abundance determines the area. Green corresponds to Simple, light blue to Hybrid, purple to Complex, yellow to sulfated or phosphorylated, and grey to unglycosylated peptides and other edge cases identified by the softwares. Percentages of a pie chart are placed in clockwise order around it, starting from the twelve-o’clock position; they appear in the following order, skipping fully absent categories: Sulfated, Simple, Other, Hybrid, Complex.

We show in Figure 2 a visualization of the degrees of *N*-glycan biosynthetic processing observed by each of the three softwares described above. These pie charts show the totals of the logged abundance for each site; we only show this for four of the sites on the S1 subunit here, but more can be found in Supplemental Figure S5. Figure 2 begins to show our difficulties in comparing results. The four selected sites of Figure 2 were chosen for the shared observance across all programs in both datasets and to contrast the results to a significant finding of Watanabe et al. They found a high mannose distribution of glycopeptides on site N234 and how this may be a shielding mechanism for binding sites. None of the searches performed here found this site to be predominantly unprocessed, high mannose glycans. Byonic found it to be wholly complex glycans or unglycosylated, GlycReSoft found a mixture with no predominating type, and MSFragger found a similar result to GlycReSoft. 

In the selected four sites, the closest sites to a consensus are that of N657, which found a predominantly complex site with a low number of simple and hybrid glycans, and N282, which saw largely complex glycans but with phosphorylation appearing in GlycReSoft. Differences arise at N122, showing GlycReSoft and MSFragger agree over complex glycan occupancy, but GlycReSoft and Byonic have a closer consensus over simple glycan occupancy. This should not be taken as a refutation of the Watanabe et al. results, but instead lead us to question: why can three different softwares, given the same error tolerances, the same PTMs, and the same data, find contrasting results from just the change in software functionality and the change in search space?

### 2.2. Zhang et al. Reanalysis

In “Site-specific *N*-glycosylation Characterization of Recombinant SARS-CoV-2 Spike Proteins” by Zhang et al. [5], the classification of the glycans differs from Watanabe et al. with high-mannose being defined as glycans with only two HexNAc, hybrid are those with three, and complex are those with more. Overall, this does not greatly impact the results of either experiment. Of the 13 glycosites observed, only one could be considered to have close to a majority high mannose, with the other 12 having predominantly complex-type glycans. Three of those complex sites were oligomannose in Watanabe et al. The most significant of those is site N234, a very dense oligomannose site and was proposed to be shielded by the protein itself the receptor-binding domain. While there is not a structure available for the S1 unit on its own, this could be viewed as confirmation that the lack of complexity in this site is due to shielding by the protein. More S1 glycopeptides were identified in this experiment than in the Watanabe experiment, which used a construct with S1 and S2 domains; the glycopeptides identified are not necessarily those of the whole protein due to the large difference in the construct.

Figure 1B shows the consensus of glycopeptides found by each software in the Zhang et al. reanalysis; these results run counter to the glycan proportion. Each software has a plurality or majority of their results unique to themselves. We can see that search space is not the predominant determinant in the differences between these results. If one were to filter post search to the glycans found only in all searches, these overlaps do not experience any large shifts in proportion. This can be seen in Figure S3c. 

**Figure 3:**
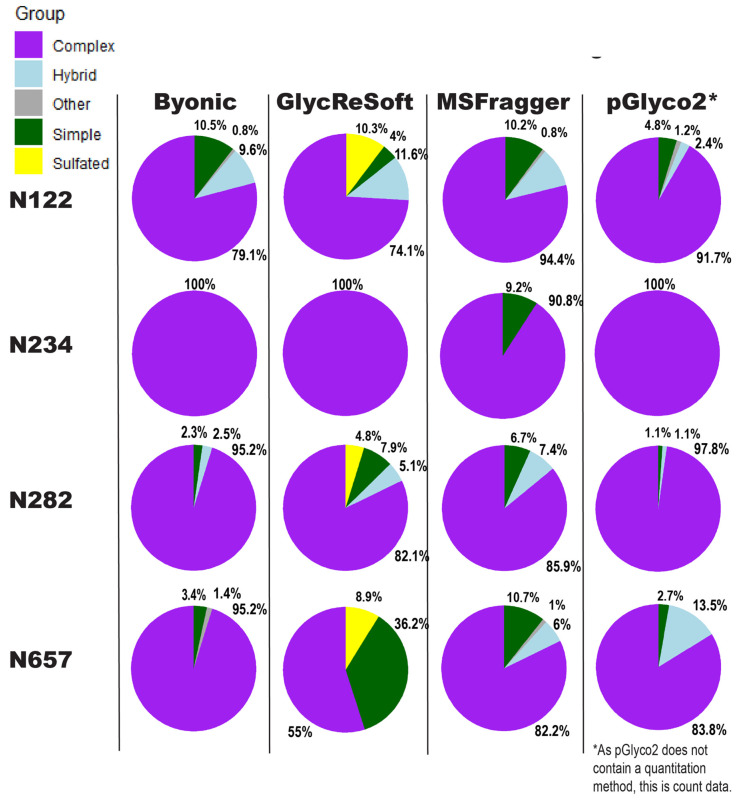
Comparison of results for searching the Zhang et al. 2021 data using Byonic, GlycReSoft, and MSFragger. Searches were conducted using the recommended settings for each software. The pie charts show the degree of *N*-glycan biosynthetic processing, with sulfated glycans included as “other”. The sum of relative log abundance determines the area. Green corresponds to Simple, light blue to Hybrid, purple to Complex, yellow to sulfated or phosphorylated, and grey to unglycosylated peptides and other edge cases identified by the softwares. Percentages of a pie chart are placed in clockwise order around it, starting from the twelve-o’clock position; they appear in the following order, skipping fully absent categories: Sulfated, Simple, Other, Hybrid, Complex.

Our comparison of glycoproteomics software performance showed a large degree of variability, as seen in Figure 3. Byonic found that of the 13 sites, five were complex, and one was evenly split between complex, hybrid, and high mannose (Figure S4a). GlycReSoft found seven were complex, and none were high mannose (Figure S4b). MSFragger-Glyco found six were complex, and none were high mannose (Figure S4c). pGlyco2 found 7 were complex (Figure S4d). As an overview, these seem predominantly consistent, but looking closer, we see varying degrees of correlation between site behavior; when looking at the individual glycopeptide compositions of a given site, there are additional dissimilarities in performance between softwares.

In Figure 3, the sites show different consensus behaviors compared to the Watanabe et al. data reanalysis. With the assumption that pGlyco2’s results are skewed by comparing spectrum counts to log abundances, N122 appears to be a fairly good consensus, with GlycReSoft differing due to both sulfation and phosphorylation. N234 would be a consensus of wholly complex glycans were it not for MSFragger’s discovery of simple glycans. N282 has differing results; all softwares agreed it was overwhelmingly complex, but GlycReSoft and MSFragger found 2–4 times the amount of simple and hybrid glycans that Byonic and pGlyco2 found, along with some phosphorylation from GlycReSoft. The most differing site is that of N657 wherein Byonic found few simple glycans among the complex. GlycReSoft found over a third of the occupancy as simple glycans, and a further tenth were phosphorylated glycans. MSFragger-Glyco found that a tenth of the signal came from simple glycans and observed hybrid glycans; pGlyco2 observed even more hybrid glycans than MSFragger.

The conclusions drawn above are affected by our inability to discern bias from physical causes, innate biology, experimental procedures, and various computational causes. For example, the oligomannose glycopeptides may be more likely to co-elute, lead to a confounding error in signal strength and cause a shorter LC gradient to result in a seemingly more complex set of glycosites. It may be that there was a sample preparation error in the single replicate runs, introducing noise and diluting the ability to identify more simple glycopeptides. It may be that the searches were unduly biased against simple glycosylations by the search space or scoring system used.

In order to address these possibilities, it is important to consider the quality of the FDR estimation. Because there remains no consensus method for building glycopeptide decoys, many of the searches may violate the law of large numbers assumptions of TDA-based FDR methods. The size of the theoretical search space is a few thousand glycopeptides, which is not very large given the assumptions of TDA [6]. Consider that the original spike protein glycoproteomics publications for the data used here found just a few hundred glycopeptides in total; at an FDR of 1%, one expects at least one false positive per hundred, but this also means that in a normal TDA setup, only one decoy was detected per hundred identifications. This means that the number of decoy glycopeptides is too few for accurate estimation of FDR. TDA has an inherently limited shelf life; as experimental quality and scoring algorithms improve, targets are more likely to outperform decoys as spectra become clearer and scoring algorithms can pick out individual points; this is especially true for glycopeptide systems wherein scores are often multifactorial, depending on the glycan and peptide components. Better scoring algorithms necessitate better decoys, but defining a better glycopeptide decoy without erasing the real gains of progress is difficult to achieve.

## 3. Discussion

For the two published data sets [1,5], we find that it is not possible to distinguish biological from computational sources for observed differences in glycoproteomics search results. We do not know which software has produced correct results, and we lack a systematic way to determine correct results. Further, we do not have a systematic way to determine reasons for the differing search engine results. The differences arise from the scope of the search space or from the probabilistic shifts in search space. They could easily come from the change in FDR calculation or the priorities of the scoring systems. They could also come from the decoy generation method. 

While efforts in standardization of output are ongoing, and various attempts at improving decoy generation and false discovery rate exist [11,14,15,16], there is not presently a way to discern their validity as methods beyond low complexity examples, and even these are not guaranteed to be accurate. The manual examination is likely to produce false negatives and can produce false positives depending on the examiner’s interpretation.

MS1 examination can help improve the reliability of the MS2 identification and quantitation tools, but they experience their own foibles and have to work with the ambiguous output of the MS2 based methodologies. MS1 identification tools such as GlycopeptideGraphMS [17] and GlycoMod [18] can be used to confirm results, but MS1 identifications are likely to be more error-prone than MS2, based informational availability, and could potentially generate false-negatives or confirm false-positives. They can serve as an invaluable check but ultimately do not eliminate the problem of certainty. Some methodologies use them to help identify and perform manual examinations, but manual examination can be variant between researchers and is impractical for larger datasets.

Other MS1 tools can be used for quantifying and qualifying datasets, such as LaCyTools [19] and SkyLine [20], but even they are not silver bullets. If the initial search results are spurious or missing options, LaCyTools and SkyLine will miss these results.

In order to accurately compare the results among softwares and the results between experiments, we must overcome the known issues. In reporting experiments, we need to define our search spaces clearly and justify the most appropriate glycan search space for a biological system. If the search space is too broad, it will include biosynthetically unreasonable glycopeptides; if too narrow, it will ignore viable and potentially significant results. It should be possible to determine a consensus, biosynthetically-reasonable and encompassing, search space for humans and model systems. While some softwares include default search spaces for humans, none of them meet the encompassing requirement. 

Reporting in studies needs to include the background proteins and other PTMs considered in a search space in order to properly assess if the law of large numbers assumption of TDA is being met; without this assumption interpreting the output of TDA-based FDR becomes increasingly error-prone. Therefore, in addition to standardizing the search space, we need to reduce the uncertainty produced by different scoring systems and FDR methods.

As it currently stands, we do not know if our decoys will suffice for our extant scoring systems. A decoy should mimic the average false score of a target and be a provably false result; glycopeptide decoys face the great difficulty of needing to mimic both peptide and glycan. Suppose the decoys fail to mimic one but not the other. In that case, there is a higher risk of undetected false-positive results since one can spuriously drive the score of decoys lower than targets since target scores will be higher if only one portion of their result matches the spectra in question which is impossible for a decoy to do by definition. This may create a diversion between the targets and decoys earlier than the assumptions of TDA provide for, which assumes two largely equivalent distributions with a heavy-tailed target distribution. This can be solved with improved decoy systems, which can be helped in development by scoring assessment tools that test the validity of TDA methods.

It is a possibility that glycoproteomics may need to shift away from TDA-based methods sooner rather than later. TDA was not designed with multimodal scoring distributions in mind, nor was it designed with filtering in mind; these can actually serve to break the assumptions of the method. Scores that simultaneously consider glycans and peptides are bound to generate multimodal scoring distributions depending on the combinations of correct and incorrect parts of potential matches. Scores which do not simultaneously consider both parts of a glycopeptide are likely to generate more false positives. And the better that a scoring system performs in identifying everything that comes out of a mass spectrometer, the less applicable TDA-based FDR becomes; decoys can only perform so well in a perfect scoring system.

These difficulties need to be addressed before we can truly discern firm conclusions on spike protein glycosylation and its evolution.

## 4. Materials and Methods

Glycopeptide searches were performed according to the recommended protocol of each software. All searches were performed on default glycan search spaces and with only the CoV-SARS2 spike protein as proteomic input; they were all performed with a 10 ppm precursor tolerance, a 20 ppm product ion tolerance, and a 1% FDR threshold or equivalent therein. Results were grouped by glycosite rather than the specific peptides acquired. 

Byonic used the 182 human *n*-glycan glycan database; GlycReSoft used a combinatorial sulfated-phosphorylated database and did not receive glycomic or proteomic input to focus the search space; MSFragger-Glyco used its standard glycan offsets; pGlyco2 used its standard glycan structure library. MSFragger-Glyco glycan offsets were labeled by glycan mass. They all produced their own quantitation’s except pGlyco2, which was quantified by the authors by spectrum count. Glycopeptide quantitations were joined in the Zhang et al datasets by averaging.

All charts were produced in RStudio using the ggplot2 and VennDiagram libraries. Venn diagrams show consensus glycans of the softwares and matching identifications of glycopeptides in the Zhang et al. dataset. Watanabe et al. are not shown here due to a low number of identifications by one of the softwares. Pie charts are made from a sum of the log abundance of the identifications by categorization as defined above. 

## 5. Conclusions

We aimed to discover the sources of the different performance observed among the glycoproteomics algorithms tested. One possibility was that the search engines matched the same MS/MS scans to different glycopeptides, suggesting that algorithm performance differences arise from the glycopeptide scoring model. Another was that that the initial glycopeptide-to-spectrum matches reported by search engines remained comparable, but different TDA methods removed different subsets of PSMs. We conclude that the results as shown do, in part, come from the search engines identifying the same MS/MS scans to different glycopeptides. But, it is currently impossible to check what glycopeptides were removed by the FDR systems due to the black-box nature of several of the scoring engines. Our view is that the FDR systems could be introducing their own biases in addition to the scoring systems, as we tried to elaborate in this narrative. These sources of bias are separate from the scoring algorithms themselves. Due to the black-box nature of several softwares and the lack of metrics and tools, it is impossible to determine the source of bias. There could be a false positive from an incorrectly highly scored glycopeptide, or there could be a false positive from a poorly performing decoy system. Without more information than is typically available, we cannot know which is the case, illustrating the need for an effort by the glycoproteomics software community to solve these problems.

## Data Availability

The Mass Spectrometry data used in this study is publicly available and can be found in the MassIVE database under MassIVE MSV000085202 for the Watanabe et al. results and in the PRIDE database under PXD018506 for the Zhang et al. results. Glycan database definitions are freely available in the case of GlycReSoft, MSFragger-Glyco, and pGlyco-2, however Byonic glycan database definitions are available with the software as metadata, which can be obtained through the free trial. Calculated abundances and specific identification lists are available upon request from the author, though a summary of these is supplied in the Supplementary information via the site-specific figures.

## Supplementary Materials

The following are available online, Figure S1a,b: Alternative Categorization for Selected Pie Charts; Figure S2: Search Space Proportion; Figure S3: Venn Diagram of Glycopeptide Identifications by Softwares; Figure S4: Tables of Log Abundance of Glycopeptides by Glycosite for Watanabe et al. reanalysis for: (a) Byonic, (b) GlycReSoft, (c) MSFragger-Glyco, (d) pGlyco2.; Figure S5: Tables of Log Abundance of Glycopeptides by Glycosite for Zhang et al. Reanalysis for: (a) Byonic, (b) GlycReSoft, (c) MSFragger-Glyco, (d) pGlyco2.

## Sample Availability

Samples of the compounds would need to be requested from the original studies’ authors.
