# Supplementary material for: The Need for Community Standards to Enable Accurate Comparison of Glycoproteomics Algorithm Performance
Source: Molecules. 2021 Aug 6;26(16):4757. doi: 10.3390/molecules26164757 (PMC8398183; doi:10.3390/molecules26164757)
Supplement: Supplementary file 1 [file molecules-26-04757-s001.zip › molecules-1179825-supplementary.pdf]

The need for community standards to enable accurate comparison of glycoproteomics algorithm performance

WE Hackett, J Zaia

Supplementary Information
Table of Contents

Methods 2
1. Search Space Definition 2
2. Search Protocols 2
3. Data Visualization and Comparison 2

Figures 4
1. Pie Charts With Sulfated/Phosphorylated not other 4
2. Search Space Proportion by Categorization 6
3. Venn Diagrams 7
    a. Glycan Search Spaces 7
    b. Watanabe et al Glycopeptide Overlap 8
    c. Zhang et al Subsetted Glycopeptide Overlap 9
4. Watanabe et al Log Abundance Tables 10
    a. Byonic 10
    b. GlycReSoft 17
    c. MSFragger-Glyco 27
5. Zhang et al Log Abundance Tables 38
    a. Byonic 38
    b. GlycReSoft 46
    c. MSFragger-Glyco 51
    d. pGlyco2 58

Supplementary Methods

Glycopeptide Search Space Definition

The glycopeptide search space for each software was defined by the fasta file given by the source paper and only used the spike protein or its subunit. The glycan were taken from default glycan search spaces for each software. The Human 182 was used for Byonic searches, and a sulfated and phosphorylated combinatorial search space was used for GlycReSoft. All searches or spaces had fixed carbamidomethylation and variable oxidation.

Glycopeptide Searches

All searches were done with a precursor tolerance of 10ppm and a product ion tolerance of 20ppm. All softwares were specified to search at 1% FDR or the equivalent therein.
Due to the differing proteases of the Watanabe et al data, the results were searched separately for each software and then merged afterwards manually.

Instead of grouping by glycopeptide and specific peptide backbone, results were grouped by the glycosites covered. Certain sites were indistinguishable due to proximity and could have two sets of results: glycosylations that could be identified as specific to a site and ambiguous glycosylations that could have come from either site.

Byonic: Beyond the changes mentioned above the recommended procedure for glycopeptide analysis in the Byonic user manual was performed using the Byologic software set for quantitative analysis. A score threshold of 100 was used.
GlycReSoft: GlycReSoft search parameters were left at default, but did not use the permuted glycan option or the newer multipart search due to the incompatibility of the data available.
MSFragger-Glyco: MSFragger-Glyco followed the recommended protocol for glycopeptides.
https://msfragger.nesvilab.org/tutorial_glyco-fragger.html
pGlyco2: pGlyco2 followed the recommended protocol. As pGlyco2 lacks quantitative options, spectrum counts were used instead.

The data quantitation, while not reported upon in the article, showed consistent abundance ranges for Byonic, GlycReSoft, and MSFragger-Glyco.

Data Visualization and Comparison

Glycopeptide grouping- Glycopeptides were grouped by glycosite rather than peptide backbone. Glycopeptides with PTMs were treated as equivalent to the unmodified glycopeptides Oxidated glycopeptides were considered equivalent to un-oxidated glycopeptides.

Glycopeptide categorization- Glycopeptides were classified as Simple, Hybrid, Complex, and Other. Simple have only two HexNAc; Hybrid gave three; Complex have more than four. Other, as shown in the main paper, are sulfated and phosphorylated glycans as well as glycans with less than two HexNAc.

Glycopeptide Consensus Venn Diagrams- Venn diagrams are used to show the overlap of the glycan search space of the softwares as well as the glycopeptides observed by each software.

The above glycopeptide grouping is used to define these glycopeptides for intersoftware comparison. While these venn diagrams are not presented here, the overlaps of individual sites that were checked showed similar behaviors overall in that the search space overlap did not seem to have any bearing on the proportion of observed glycopeptides.

Pie Charts Category Summaries- Pie charts of the total of the relative log abundances of the glycopeptides are presented. Relative log abundance is used to create a more conservative presentation of the differences between glycopeptides as abundance data that has not been logged is highly susceptible to scale issues and false perceptions of differentiation.

Glycopeptide Barplots- Barplots of the log abundances of the glycopeptides are presented below by software for each site or pair of sites. For reference of the log scale: 11.5 is roughly equivalent to 1E5, 16.1 is roughly equivalent to 1E7, and 20.7 is roughly equivalent to 1E9. The log scale can give an impression of false uniformity compared to the more familiar relative abundance plots, but it does prevent a false impression of differentiation. Certain glycosites were too populated to be presented as one image and so were split.

Supplementary Figures

Comparison Figures if Sulfated and Phosphorylated Glycans are not classified as Other

Supplementary Figure S1a: Here we see the results of the abundance summations if the sulfated groups were not considered other by default. We see that the proportion of 'other' does not consistently go to one classification compared to the original delineation.

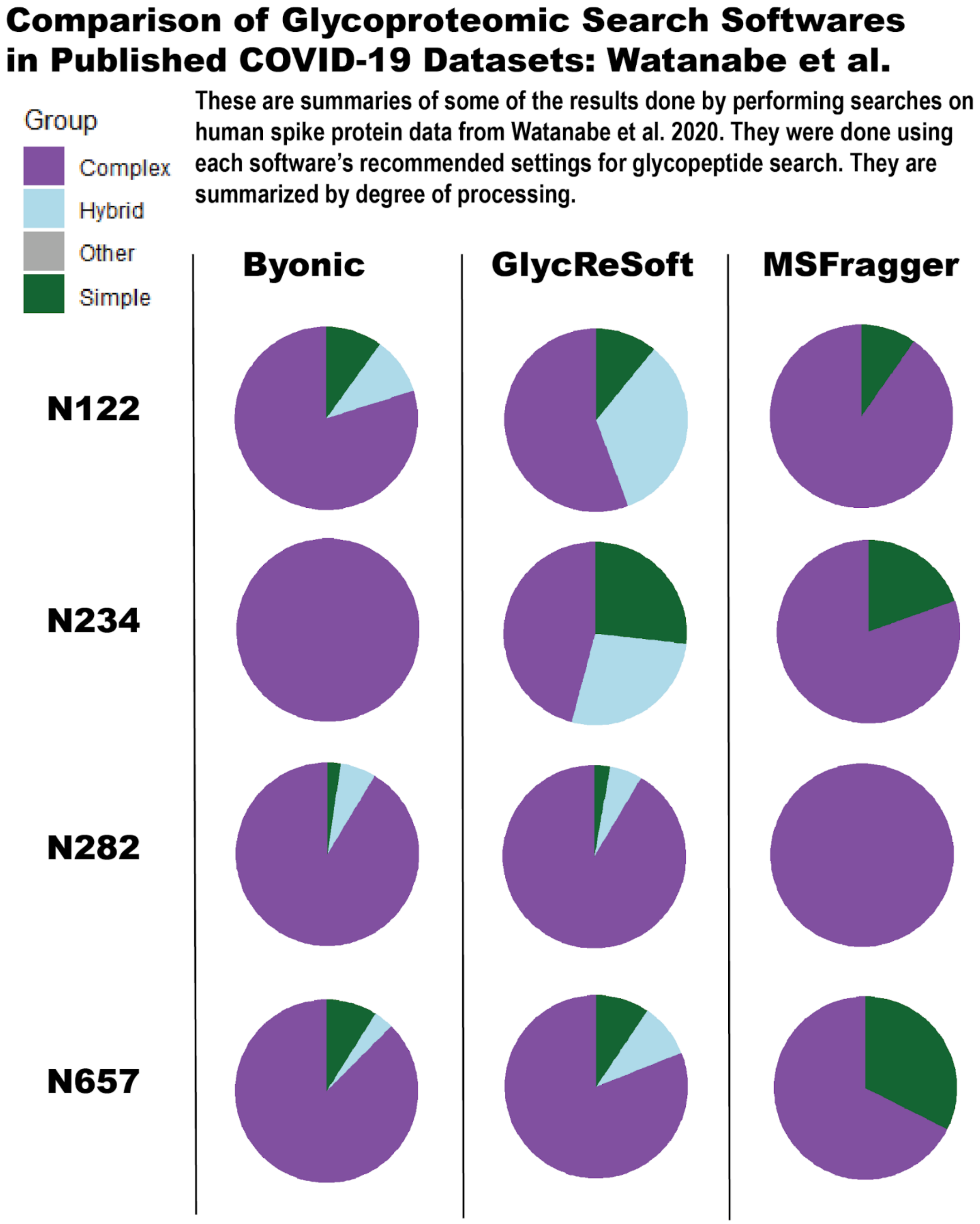

Supplementary Figure S1b

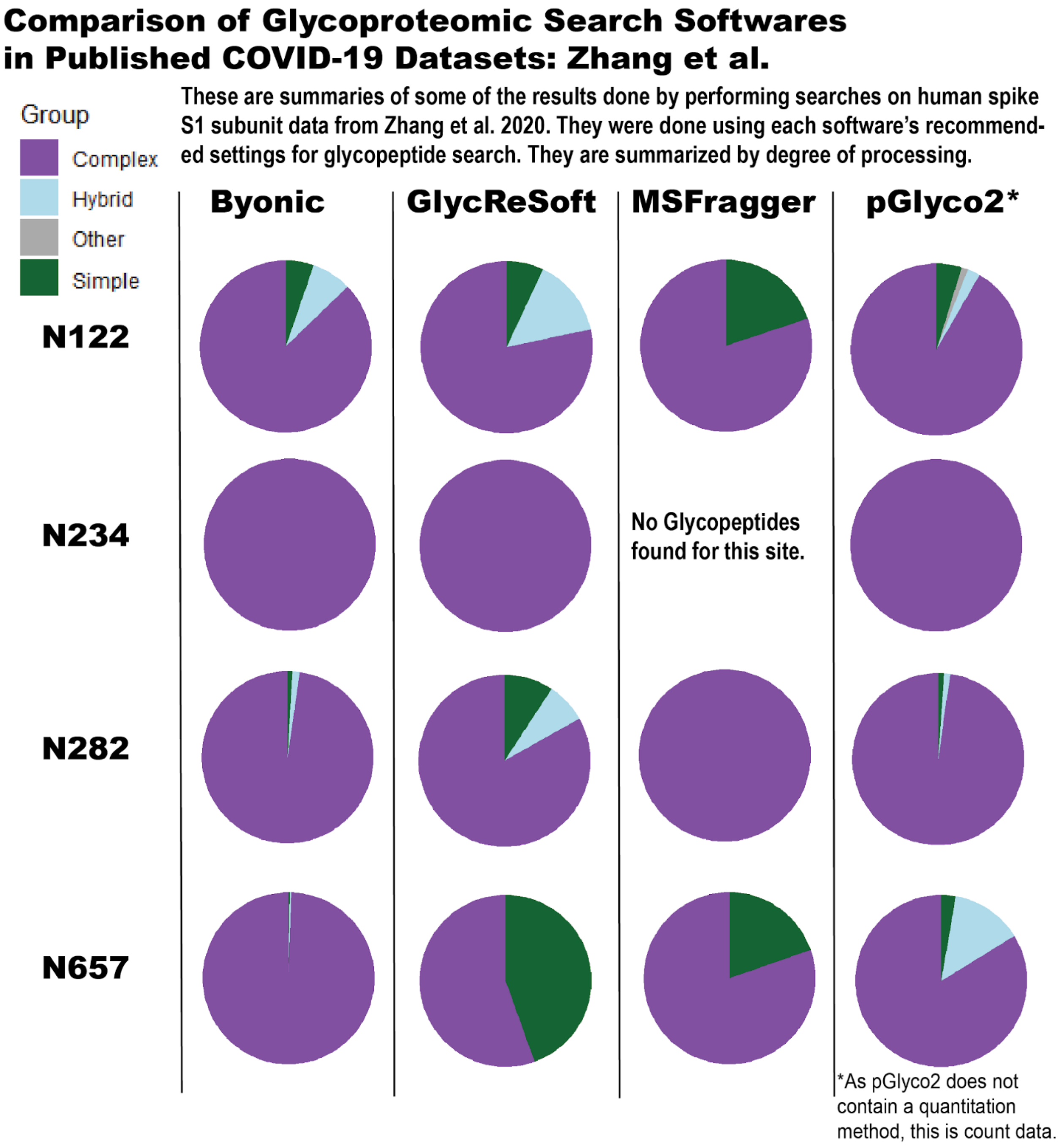

Supplementary Figure S2
This is a graph of the search space broken by glycan type by software. The only search space that does not contain overwhelmingly complex type glycans is GlycReSoft, which is disproportionately other type. The Joint column represents the intersection of all search spaces, and has a slightly higher proportion of Hybrid type glycans than any individual software but is still overwhelmingly complex.

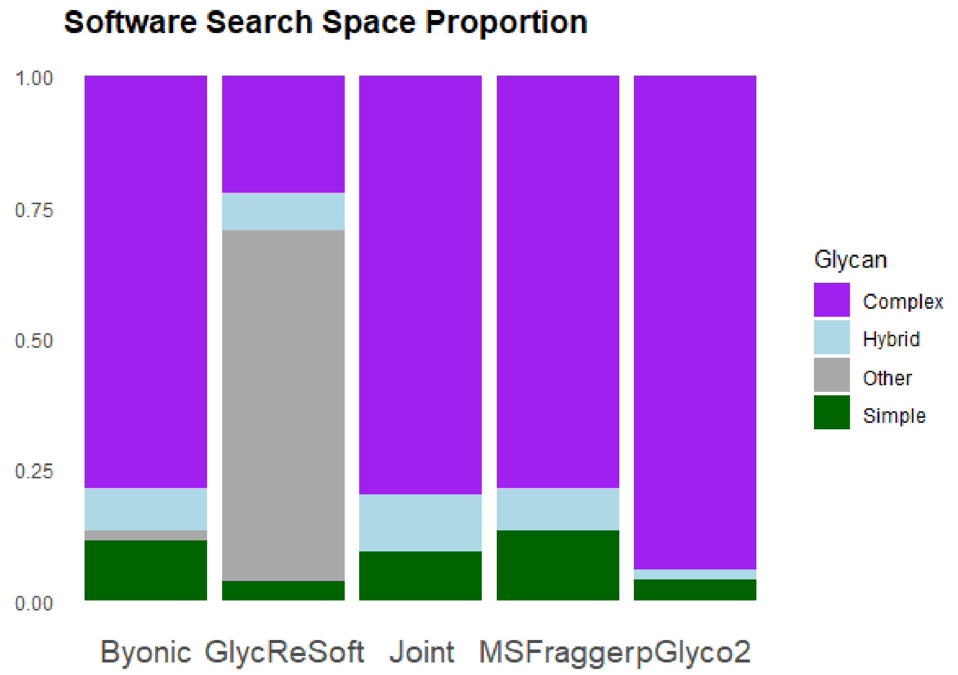

This is a graph of the search space proportion as broken down by glycan type. This graph does not take into account sulfation or phosphorylation. As can be seen, all softwares are predominantly complex type glycans in this classification schema.

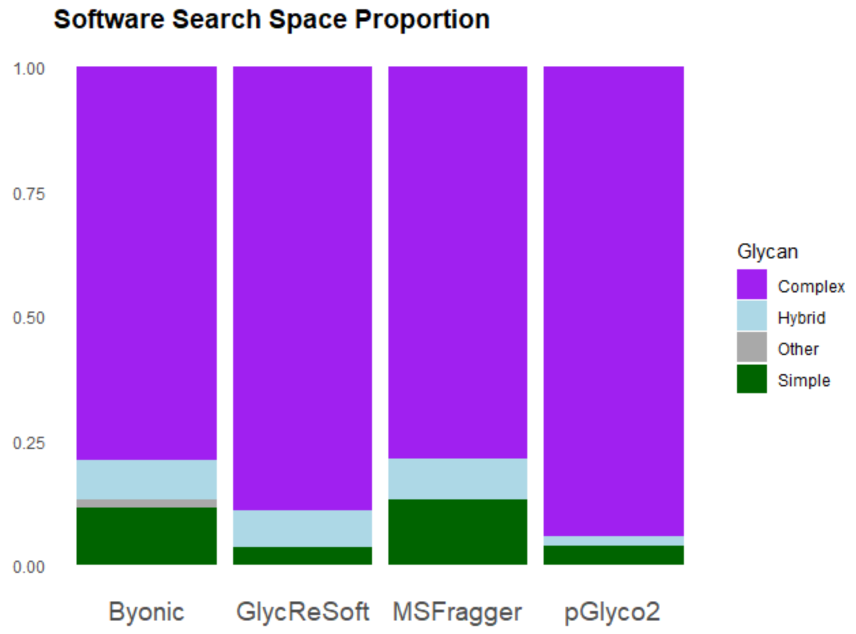

Figure S3a. Venn Diagram of Software Glycan Searchspaces
The following venn diagram represents the search space overlap.

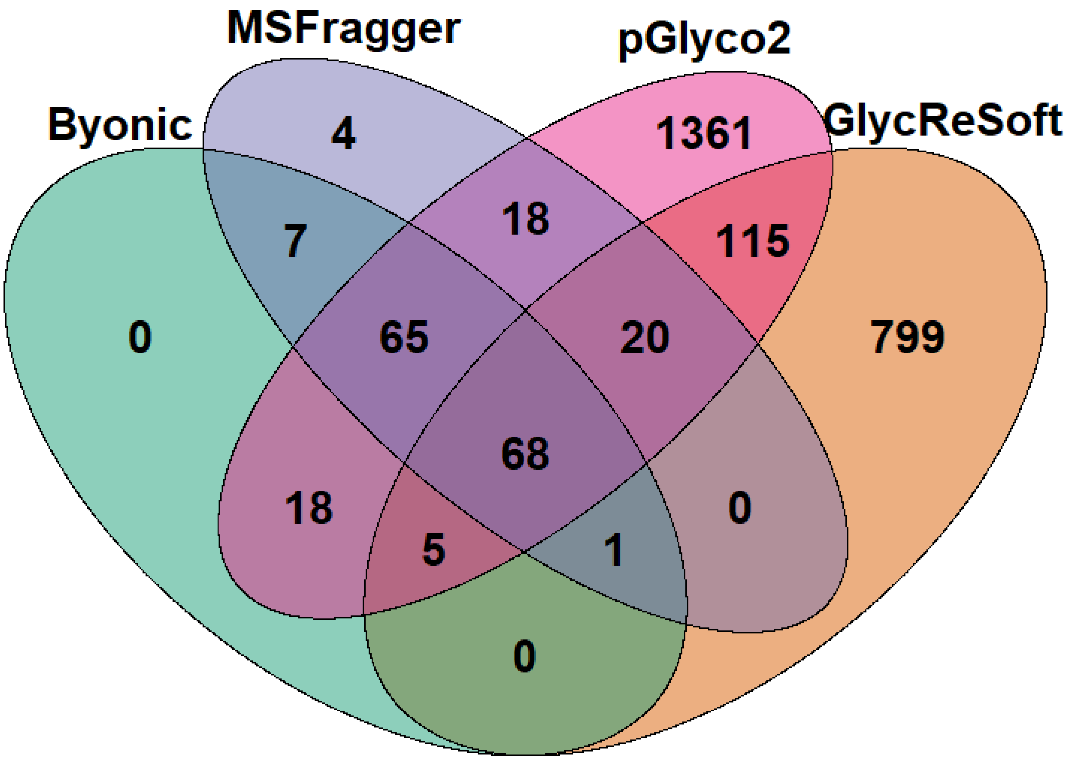

Figure S3b. Venn Diagram of Glycopeptide Searches of the Watanabe et al data
A venn diagram of the three softwares successfully applied to the Watanabe et al data. It shows a high proportion of overlap between GlycReSoft and MSFragger-Glyco.

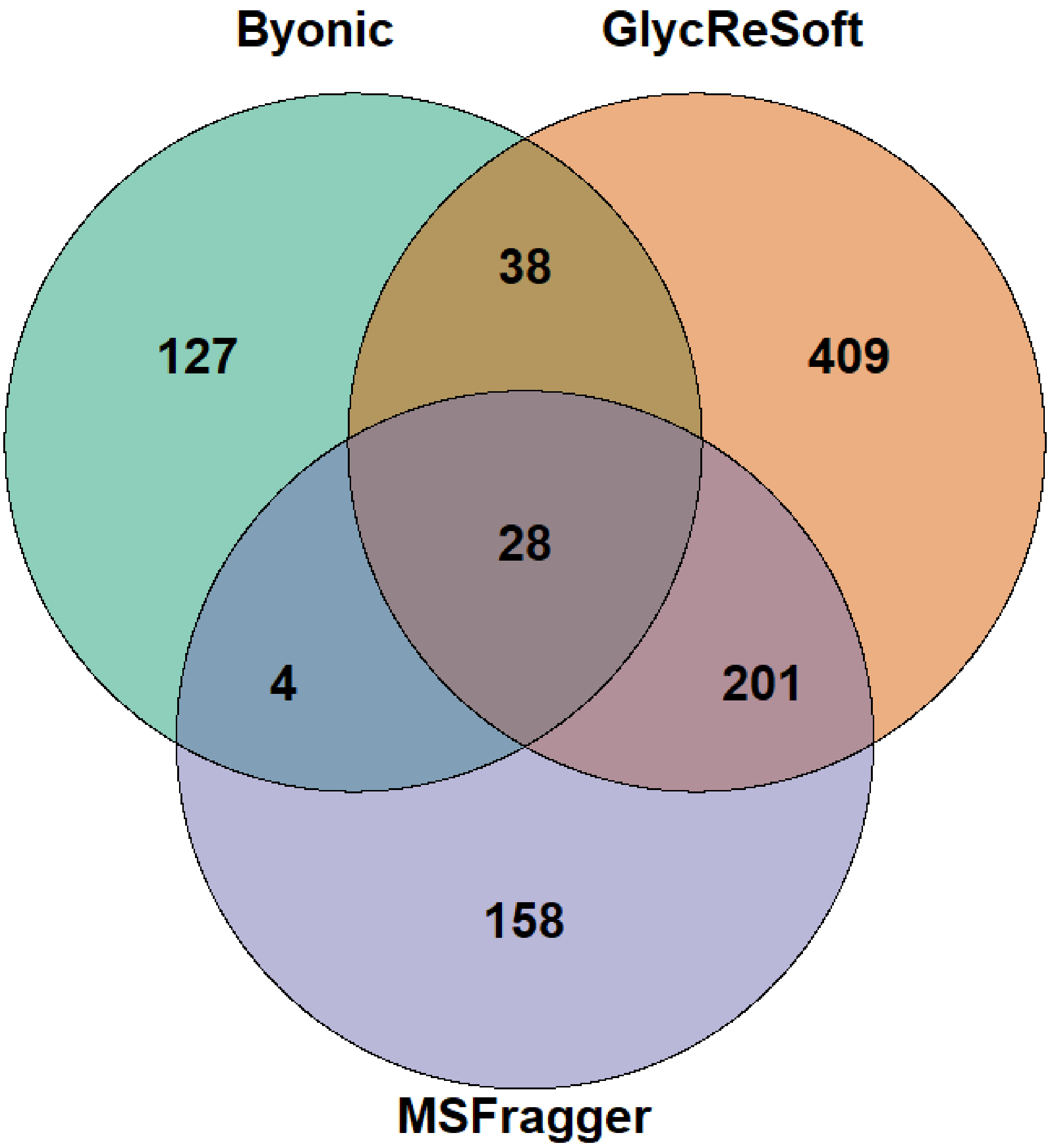

Figure S3c. Zhang et al Venn diagram of results.
On the left is presented the results when subset down to the 119 common glycans. The right is the original Zhang et al results. There is little shift in the relative proportion of overlapped results, but pGlyco2 and GlycReSoft experience a drop in unique observations, which follows from the correlation to their larger and more unique search spaces.

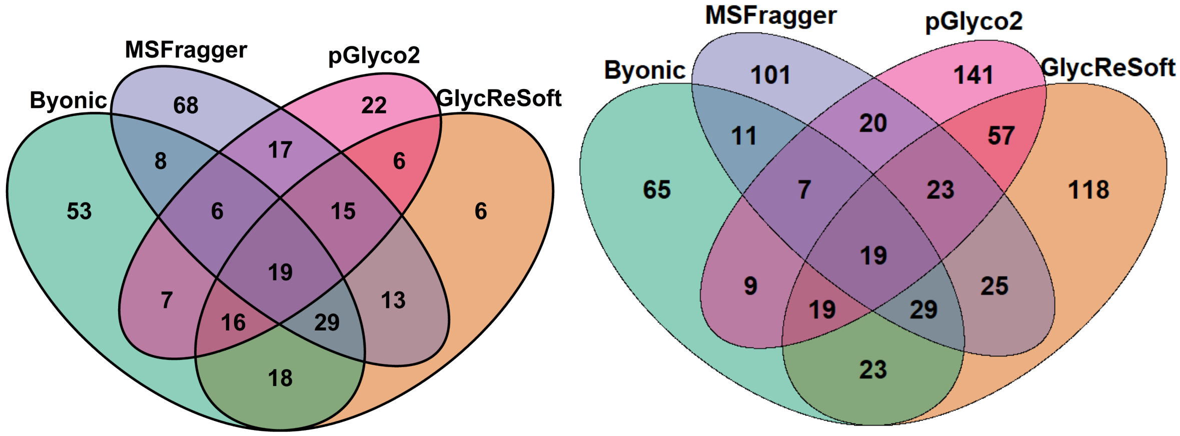

Figure S4. Watanabe et al Searches by Site

Glycopeptide Format: [Hex;HexNAc;NeuAc;Fuc;Phosphate/Sulfate*]

Phosphate/Sulfate is an indicator variable instead of a quantifier: 0 indicates neither, 1 indicates a phosphorylated glycan, 2 indicates a sulfated glycan.

Figure S4a. Watanabe et al search using Byonic

Site	AUC	Glycan
17	1.59E+09	[5;6;3;0;1;0]
17	22800000	[7;5;2;0;1;0]

Site	AUC	Glycan
74	2065293125	[]
61,74	85100000	[10;6;1;0;1;0]
61,74	277400000	[11;11;1;0;0;0]
61,74	30300000	[3;6;2;0;1;0]
61,74	44400000	[5;6;2;0;1;0]
61,74	39400000	[9;6;2;0;1;0]

Site	AUC	Glycan
122	841394117.6	[]
122	155130769.2	[0;1;0;0;0;0]
122	16001666.67	[0;1;0;0;1;0]
122	51885000	[1;2;0;0;0;0]
122	346550000	[10;2;0;0;0;0]
122	1.03E+08	[10;6;1;0;1;0]
122	68300000	[10;9;0;0;0;0]
122	20500000	[2;2;0;0;0;0]
122	105954545.5	[3;2;0;0;0;0]
122	72457500	[3;3;0;0;0;0]
122	100865357.1	[3;3;0;0;1;0]
122	591066666.7	[3;4;0;0;0;0]
122	1475429167	[3;4;0;0;1;0]
122	33500000	[3;4;1;0;0;0]
122	647095454.5	[3;5;0;0;0;0]
122	1876280556	[3;5;0;0;1;0]
122	256022222.2	[3;5;1;0;1;0]
122	1178888214	[3;6;0;0;0;0]
122	974465000	[3;6;0;0;1;0]
122	164750000	[3;6;1;0;1;0]
122	207150000	[3;7;0;0;0;0]
122	224507142.9	[3;7;0;0;1;0]
122	24600000	[3;8;0;0;1;0]
122	198616842.1	[4;2;0;0;0;0]
122	47030000	[4;2;0;0;1;0]
122	419464285.7	[4;3;0;0;0;0]
122	289732758.6	[4;3;0;0;1;0]
122	42723750	[4;3;1;0;0;0]
122	114040909.1	[4;3;1;0;1;0]
122	669157142.9	[4;4;0;0;0;0]
122	1292309524	[4;4;0;0;1;0]
122	705704166.7	[4;4;1;0;0;0]
122	1248335000	[4;4;1;0;1;0]
122	1261296667	[4;5;0;0;0;0]
122	3478695238	[4;5;0;0;1;0]
122	2999539706	[4;5;1;0;0;0]
122	2962625000	[4;5;1;0;1;0]
122	541550000	[4;5;2;0;0;0]
122	382470000	[4;5;2;0;1;0]
122	716169230.8	[4;6;0;0;0;0]
122	1629396667	[4;6;0;0;1;0]
122	464983333.3	[4;6;1;0;0;0]
122	1.60E+08	[4;7;0;0;0;0]

122	169275000	[4;7;0;0;1;0]
122	28945000	[4;8;0;0;0;0]
122	3032018519	[5;2;0;0;0;0]
122	1058360714	[5;3;0;0;0;0]
122	958741538.5	[5;3;0;0;1;0]
122	745931250	[5;3;1;0;0;0]
122	1673157895	[5;4;0;0;0;0]
122	5654701111	[5;4;0;0;1;0]
122	3253392000	[5;4;1;0;0;0]
122	18328386250	[5;4;1;0;1;0]
122	547159230.8	[5;4;2;0;0;0]
122	4768033333	[5;4;2;0;1;0]
122	2481240741	[5;5;0;0;0;0]
122	12113870238	[5;5;0;0;1;0]
122	4665992221	[5;5;1;0;0;0]
122	7650920455	[5;5;1;0;1;0]
122	956220138.9	[5;5;2;0;0;0]
122	335180666.7	[5;5;2;0;1;0]
122	2407017727	[5;6;0;0;0;0]
122	1260386842	[5;6;0;0;1;0]
122	732041833.3	[5;6;1;0;1;0]
122	133299090.9	[5;6;2;0;1;0]
122	49300000	[5;8;0;0;0;0]
122	35200000	[5;8;0;0;1;0]
122	1934615385	[6;2;0;0;0;0]
122	1550507692	[6;3;0;0;0;0]
122	1110479412	[6;3;0;0;1;0]
122	1403808333	[6;3;1;0;0;0]
122	431463636.4	[6;3;1;0;1;0]
122	1245388889	[6;4;0;0;0;0]
122	1825033333	[6;4;0;0;1;0]
122	2488293750	[6;4;1;0;0;0]
122	863790000	[6;4;1;0;1;0]
122	3939323077	[6;5;0;0;0;0]
122	2221125000	[6;5;0;0;1;0]
122	2403305556	[6;5;1;0;0;0]
122	3530026923	[6;5;1;0;1;0]
122	802207142.9	[6;5;2;0;0;0]
122	1383254054	[6;5;2;0;1;0]
122	159308750	[6;5;3;0;0;0]
122	193890909.1	[6;5;3;0;1;0]
122	801938235.3	[6;6;0;0;0;0]
122	2994816667	[6;6;0;0;1;0]
122	1227878125	[6;6;1;0;0;0]
122	1663772727	[6;6;1;0;1;0]
122	424318750	[6;6;2;0;0;0]
122	503353333.3	[6;6;2;0;1;0]
122	523533333.3	[6;7;0;0;0;0]
122	346228437.5	[6;7;0;0;1;0]
122	1996777778	[7;2;0;0;0;0]
122	79013000	[7;4;0;0;0;0]
122	427350000	[7;5;0;0;1;0]
122	326779166.7	[7;5;1;0;1;0]
122	79997142.86	[7;5;2;0;1;0]
122	893516666.7	[7;6;0;0;0;0]
122	654242307.7	[7;6;0;0;1;0]
122	501110000	[7;6;1;0;0;0]
122	672682857.1	[7;6;1;0;1;0]
122	225058823.5	[7;6;2;0;0;0]
122	560217187.5	[7;6;2;0;1;0]
122	101153500	[7;6;3;0;0;0]
122	217205384.6	[7;6;3;0;1;0]
122	2.20E+07	[7;6;4;0;0;0]
122	132074500	[7;7;0;0;0;0]
122	611347058.8	[7;7;0;0;1;0]
122	433047696.4	[7;7;2;0;1;0]
122	103299166.7	[7;7;3;0;1;0]
122	100320000	[7;8;0;0;0;0]
122	1721558333	[8;2;0;0;0;0]
122	190984000	[8;6;1;0;0;0]

122	109480000	[8;6;1;0;1;0]
122	156331111.1	[8;7;0;0;0;0]
122	124653076.9	[8;7;0;0;1;0]
122	167146750	[8;7;1;0;0;0]
122	121935312.5	[8;7;1;0;1;0]
122	84413888.89	[8;7;2;0;1;0]
122	21544833.33	[8;7;3;0;1;0]
122	88891250	[8;8;0;0;0;0]
122	37021500	[8;8;0;0;1;0]
122	1432330000	[9;2;0;0;0;0]
122	24800000	[9;6;2;0;1;0]
122	15400000	[9;8;0;0;0;0]
122	79246250	[9;8;0;0;1;0]
122	8510000	[9;8;3;0;1;0]
122	5640000	[9;9;0;0;1;0]

Site	AUC	Glycan
149	10100000	[6;5;2;0;1;0]
149	16311833.33	[6;5;3;0;1;0]
149	11600000	[6;6;1;0;1;0]
149	18600000	[6;6;2;0;1;0]
149	15294166.67	[7;6;4;0;1;0]
149	12700000	[7;7;2;0;1;0]

Site	AUC	Glycan
165	2.48E+08	[3;5;0;0;0;0]
165	3.35E+08	[3;6;0;0;0;0]
165	2.39E+08	[3;7;0;0;0;0]
165	19885000	[4;5;1;0;0;0]
165	286225000	[4;5;2;0;1;0]
165	254066666.7	[4;6;0;0;0;0]
165	2.91E+08	[4;6;0;0;1;0]
165	175666666.7	[4;6;1;0;0;0]
165	2.11E+08	[5;4;0;0;0;0]
165	26800000	[5;4;0;0;1;0]
165	32400000	[5;4;2;0;1;0]
165	515471428.6	[5;5;0;0;0;0]
165	693230156.3	[5;5;1;0;0;0]
165	90200000	[5;5;1;0;1;0]
165	1.88E+08	[5;5;2;0;0;0]
165	4.07E+08	[5;6;0;0;0;0]
165	70200000	[6;4;1;0;0;0]
165	1.10E+08	[6;6;1;0;0;0]
165	64100000	[6;6;1;0;1;0]
165	84600000	[6;6;2;0;0;0]
165	37300000	[7;6;2;0;1;0]
165	1.68E+08	[7;6;4;0;1;0]
165	3.30E+07	[7;7;0;0;1;0]
165	39100000	[8;7;1;0;0;0]
165	2.45E+08	[8;7;3;0;1;0]
165	64200000	[9;8;0;0;0;0]

Site	AUC	Glycan
234	290808333.3	[]
234	9820000	[4;4;1;0;1;0]
234	51166666.67	[5;5;1;0;0;0]
234	5470000	[5;5;2;0;0;0]
234	37400000	[5;6;1;0;1;0]
234	18300000	[5;6;3;0;1;0]
234	16900000	[6;4;1;0;1;0]
234	34900000	[6;5;3;0;0;0]
234	11329666.67	[6;5;3;0;1;0]
234	40500000	[6;6;2;0;0;0]
234	9441250	[6;7;4;0;1;0]
234	195333333.3	[7;6;2;0;1;0]
234	8476333.333	[7;6;3;0;1;0]
234	12646428.57	[7;6;4;0;0;0]
234	169938076.9	[7;6;4;0;1;0]
234	10915500	[7;7;4;0;1;0]
234	3540000	[8;7;0;0;0;0]

234	59672500	[8;7;1;0;0;0]
234	11300000	[8;7;2;0;1;0]
234	44169000	[8;7;3;0;1;0]
234	34579545.45	[8;7;4;0;1;0]
234	1.50E+07	[9;8;4;0;1;0]

Site	AUC	Glycan
282	1229153846	[]
282	32400000	[]
282	1.78E+08	[10;9;0;0;0;0]
282	93290000	[10;9;0;0;1;0]
282	638158333.3	[3;5;0;0;1;0]
282	1.16E+08	[3;5;1;0;1;0]
282	401092857.1	[3;6;0;0;0;0]
282	570560714.3	[3;6;0;0;1;0]
282	509305000	[3;6;1;0;1;0]
282	204416666.7	[3;7;0;0;0;0]
282	334093750	[3;7;0;0;1;0]
282	56596666.67	[3;8;0;0;0;0]
282	249966666.7	[3;9;0;0;0;0]
282	10300000	[4;2;0;0;0;0]
282	21900000	[4;3;0;0;1;0]
282	23528333.33	[4;3;1;0;1;0]
282	3.00E+08	[4;4;0;0;0;0]
282	2.44E+08	[4;4;0;0;1;0]
282	306383333.3	[4;4;1;0;0;0]
282	171264444.4	[4;4;1;0;1;0]
282	529810000	[4;5;0;0;0;0]
282	849135227.3	[4;5;0;0;1;0]
282	933484375	[4;5;1;0;0;0]
282	567577727.3	[4;5;1;0;1;0]
282	1149275000	[4;5;2;0;0;0]
282	116553636.4	[4;5;2;0;1;0]
282	282268750	[4;6;0;0;0;0]
282	564782142.9	[4;6;0;0;1;0]
282	346740357.1	[4;6;1;0;0;0]
282	161407142.9	[4;7;0;0;0;0]
282	119767222.2	[4;7;0;0;1;0]
282	2760000	[4;8;0;0;0;0]
282	118794285.7	[5;3;1;0;0;0]
282	714142272.7	[5;4;0;0;0;0]
282	995527868.4	[5;4;0;0;1;0]
282	831935333.3	[5;4;1;0;0;0]
282	1677268421	[5;4;1;0;1;0]
282	453460000	[5;4;2;0;0;0]
282	605268571.4	[5;4;2;0;1;0]
282	705188461.5	[5;5;0;0;0;0]
282	1659087500	[5;5;0;0;1;0]
282	695501851.9	[5;5;1;0;0;0]
282	976132000	[5;5;1;0;1;0]
282	162024000	[5;5;2;0;0;0]
282	55183888.89	[5;5;2;0;1;0]
282	700542307.7	[5;6;0;0;0;0]
282	413484375	[5;6;0;0;1;0]
282	486455555.6	[5;6;1;0;1;0]
282	141108181.8	[5;6;2;0;1;0]
282	308356250	[5;6;3;0;1;0]
282	247708333.3	[6;3;0;0;0;0]
282	146180000	[6;3;0;0;1;0]
282	143320000	[6;3;1;0;0;0]
282	56458333.33	[6;3;1;0;1;0]
282	216201071.4	[6;4;0;0;0;0]
282	4.80E+08	[6;4;0;0;1;0]
282	638549615.4	[6;4;1;0;0;0]
282	328641666.7	[6;4;1;0;1;0]
282	331828333.3	[6;5;0;0;0;0]
282	626807357.1	[6;5;0;0;1;0]
282	311900000	[6;5;1;0;0;0]
282	947795526.3	[6;5;1;0;1;0]
282	479062500	[6;5;2;0;0;0]

282	499303421.1	[6;5;2;0;1;0]
282	229258000	[6;5;3;0;0;0]
282	326279166.7	[6;5;3;0;1;0]
282	332305555.6	[6;6;0;0;0;0]
282	452058740	[6;6;0;0;1;0]
282	408999032.3	[6;6;1;0;0;0]
282	613195972.2	[6;6;1;0;1;0]
282	468526470.6	[6;6;2;0;0;0]
282	137030714.3	[6;6;2;0;1;0]
282	1.62E+08	[6;7;0;0;0;0]
282	162769285.7	[6;7;0;0;1;0]
282	40100000	[6;7;4;0;1;0]
282	30100000	[6;8;0;0;0;0]
282	18400000	[7;4;0;0;0;0]
282	59200000	[7;4;0;0;1;0]
282	19179000	[7;4;1;0;0;0]
282	430975000	[7;5;0;0;0;0]
282	388410500	[7;5;0;0;1;0]
282	163515833.3	[7;5;1;0;1;0]
282	1.63E+08	[7;5;2;0;1;0]
282	263416666.7	[7;6;0;0;0;0]
282	428880000	[7;6;0;0;1;0]
282	638426190.5	[7;6;1;0;0;0]
282	677296153.8	[7;6;1;0;1;0]
282	550485250	[7;6;2;0;0;0]
282	853678437.5	[7;6;2;0;1;0]
282	560212500	[7;6;3;0;0;0]
282	228628214.3	[7;6;3;0;1;0]
282	295781250	[7;6;4;0;0;0]
282	169565681.8	[7;6;4;0;1;0]
282	149535000	[7;7;0;0;0;0]
282	273323125	[7;7;0;0;1;0]
282	726266562.5	[7;7;2;0;1;0]
282	405038888.9	[7;7;3;0;1;0]
282	197900000	[7;7;4;0;1;0]
282	45063750	[7;8;0;0;0;0]
282	228916666.7	[8;2;0;0;0;0]
282	368820357.1	[8;6;1;0;0;0]
282	407966666.7	[8;6;1;0;1;0]
282	226346153.8	[8;7;0;0;0;0]
282	237750000	[8;7;0;0;1;0]
282	508216807.7	[8;7;1;0;0;0]
282	500779629.6	[8;7;1;0;1;0]
282	641582142.9	[8;7;2;0;1;0]
282	2.06E+08	[8;7;3;0;1;0]
282	86293333.33	[8;7;4;0;1;0]
282	128272000	[8;8;0;0;0;0]
282	228027777.8	[8;8;0;0;1;0]
282	124331250	[9;2;0;0;0;0]
282	298400000	[9;6;2;0;1;0]
282	4220000	[9;8;0;0;0;0]
282	86500000	[9;8;0;0;1;0]
282	61498000	[9;9;0;0;1;0]

Site	AUC	Glycan
331	4.70E+07	[]
343	2402850000	[]
331,343	173500000	[11;11;1;0;0;0]
331,343	53300000	[3;6;2;0;1;0]
331,343	118800000	[5;6;2;0;1;0]
331,343	1.13E+08	[5;6;3;0;1;0]
331,343	69100000	[6;6;2;0;0;0]
331,343	57300000	[6;6;2;0;1;0]
331,343	87400000	[6;6;3;0;1;0]
331,343	1.20E+08	[6;9;0;0;1;0]
331,343	90650000	[7;6;2;0;0;0]
331,343	31400000	[7;6;3;0;1;0]
331,343	87288333.33	[7;7;3;0;1;0]
331,343	4.92E+08	[7;7;4;0;1;0]
331,343	52150000	[8;7;0;0;1;0]

331,343	67100000	[8;7;3;0;1;0]
331,343	112750000	[8;8;0;0;1;0]
331,343	76700000	[9;6;2;0;1;0]

Site	AUC	Glycan
603,616	40800000	[3;6;2;0;1;0]
603,616	4650000	[8;6;1;0;0;0]

Site	AUC	Glycan
657	2.25E+08	[]
657	34600000	[10;2;0;0;0;0]
657	1.73E+08	[10;9;0;0;0;0]
657	4.40E+07	[10;9;0;0;1;0]
657	11600000	[11;2;0;0;0;0]
657	37800000	[2;2;0;0;0;0]
657	8970000	[3;4;0;0;0;0]
657	52600000	[3;4;0;0;1;0]
657	47400000	[3;5;0;0;0;0]
657	28450000	[3;6;0;0;1;0]
657	219853125	[3;6;1;0;1;0]
657	42338333.33	[3;7;0;0;1;0]
657	68701428.57	[3;8;0;0;1;0]
657	88700000	[3;9;0;0;1;0]
657	82200000	[4;2;0;0;0;0]
657	145610000	[4;3;1;0;0;0]
657	56200000	[4;4;0;0;1;0]
657	43300000	[4;4;1;0;0;0]
657	8152500	[4;5;0;0;0;0]
657	257440000	[4;5;0;0;1;0]
657	86180000	[4;5;1;0;0;0]
657	70400000	[4;5;1;0;1;0]
657	4.70E+08	[4;5;2;0;0;0]
657	36400000	[4;5;2;0;1;0]
657	112234166.7	[4;6;0;0;1;0]
657	115278750	[4;6;1;0;0;0]

657	36200000	[4;7;0;0;0;0]
657	12005833.33	[4;8;0;0;0;0]
657	11800000	[4;9;0;0;1;0]
657	68700000	[5;2;0;0;0;0]
657	60500000	[5;4;0;0;1;0]
657	72296666.67	[5;4;1;0;0;0]
657	174202500	[5;4;1;0;1;0]
657	44300000	[5;4;2;0;0;0]
657	2.64E+08	[5;5;0;0;0;0]
657	204300000	[5;5;0;0;1;0]
657	236130000	[5;5;1;0;0;0]
657	361854000	[5;5;1;0;1;0]
657	8640000	[5;5;2;0;0;0]
657	9.60E+07	[5;5;2;0;1;0]
657	24400000	[5;6;0;0;0;0]
657	38800000	[5;6;0;0;1;0]
657	31075000	[5;6;2;0;1;0]
657	31800000	[5;6;3;0;1;0]
657	51100000	[6;2;0;0;0;0]
657	48340000	[6;3;1;0;0;0]
657	89600000	[6;3;1;0;1;0]
657	33300000	[6;4;0;0;0;0]
657	15600000	[6;4;0;0;1;0]
657	86800000	[6;4;1;0;0;0]
657	10900000	[6;5;0;0;0;0]
657	133347500	[6;5;1;0;1;0]
657	91936666.67	[6;5;2;0;0;0]
657	1.99E+08	[6;5;2;0;1;0]
657	3.90E+07	[6;5;3;0;0;0]
657	1.37E+08	[6;6;0;0;0;0]
657	179883333.3	[6;6;0;0;1;0]
657	81850000	[6;6;1;0;0;0]
657	2.23E+08	[6;6;1;0;1;0]
657	47550000	[6;6;2;0;0;0]
657	49500000	[6;6;2;0;1;0]

657	87250000	[6;6;3;0;1;0]
657	48100000	[6;7;0;0;1;0]
657	52951666.67	[7;4;0;0;0;0]
657	85700000	[7;5;0;0;1;0]
657	4130000	[7;5;1;0;1;0]
657	31800000	[7;5;2;0;1;0]
657	63100000	[7;6;1;0;0;0]
657	46060000	[7;6;1;0;1;0]
657	58953333.33	[7;6;2;0;0;0]
657	233391250	[7;6;2;0;1;0]
657	68300000	[7;6;3;0;0;0]
657	233100000	[7;6;3;0;1;0]
657	121483333.3	[7;6;4;0;0;0]
657	59595000	[7;6;4;0;1;0]
657	244800000	[7;7;2;0;1;0]
657	38200000	[7;7;3;0;1;0]
657	27100000	[8;2;0;0;0;0]
657	34300000	[8;6;1;0;0;0]
657	7.30E+07	[8;6;1;0;1;0]
657	16600000	[8;7;0;0;1;0]
657	13500000	[8;7;1;0;0;0]
657	42994166.67	[8;7;1;0;1;0]
657	1.56E+08	[8;7;3;0;1;0]
657	48300000	[8;7;4;0;1;0]
657	18450000	[9;6;2;0;1;0]
657	75948333.33	[9;8;3;0;1;0]

Site	AUC	Glycan
1074	26800000	[]

Figure S4b. Watanabe et al search using GlycReSoft

Site	Intensity	Glycan
17	1204629739	[3;5;1;0;0;0]
17	34202708	[3;6;0;0;2;0]
17	1149527687	[3;6;1;0;0;0]
17	4689395.671	[4;6;1;0;0;0]
17	2072018.833	[4;7;1;0;0;0]
17	94509355.23	[5;3;0;0;1;0]
17	69625825.33	[5;3;0;0;2;0]
17	42698765.77	[5;3;1;0;1;0]
17	7703931475	[8;2;0;0;0;0]
17	59635611.5	[8;2;0;0;0;1]

Watanabe et al GlycReSoft Search

Site	Intensity	Glycan
61,74	98284785.17	[10;4;0;0;0;0]
61,74	121873989	[5;3;0;0;1;0]
61,74	512602771.1	[6;8;0;0;1;0]
61,74	445639779.8	[7;8;0;0;2;0]
61,74	164768477	[8;5;0;0;1;0]
61,74	2479513313	[8;6;0;0;1;0]
61,74	172263103.5	[8;6;0;0;2;0]
61,74	6983703238	[8;7;0;0;1;0]
61,74	2807755564	[8;7;0;0;2;0]
61,74	1143866875	[8;7;1;0;1;0]
61,74	1548581312	[8;8;0;0;1;0]
61,74	1684061118	[8;8;0;0;2;0]
61,74	450830029	[8;8;0;0;3;0]
61,74	878160978.8	[9;6;0;0;1;0]
61,74	574931047.2	[9;6;0;0;2;0]
61,74	492603578.8	[9;6;1;0;1;0]
61,74	2035172156	[9;7;0;0;1;0]
61,74	685685994.3	[9;7;0;0;1;1]
61,74	2216319102	[9;7;0;0;2;0]
61,74	240947871.2	[9;7;0;0;2;1]
61,74	711485978.8	[9;7;0;0;3;0]
61,74	1736889908	[9;7;1;0;1;0]
61,74	1038713028	[9;7;1;0;1;1]
61,74	1704791247	[9;7;1;0;2;0]
61,74	1319220261	[9;7;2;0;1;0]

Watanabe et al GlycReSoft Search

Site	Intensity	Glycan
74	120042455.8	[3;2;0;0;1;0]
74	2882240687	[3;3;0;0;1;0]
74	29339255.83	[3;3;0;0;3;1]
74	16662368374	[3;4;0;0;1;0]
74	487561740.7	[3;4;0;0;1;2]
74	1214098533	[3;4;0;0;2;0]
74	132907253.1	[3;4;1;0;1;0]
74	8489039.542	[3;4;2;0;4;0]
74	29599010485	[3;5;0;0;1;0]
74	8571661720	[3;5;0;0;1;2]
74	21188971337	[3;5;0;0;2;0]
74	14155546.54	[3;5;0;0;2;2]
74	7989720.5	[3;5;0;0;3;0]
74	6548700752	[3;5;1;0;1;0]
74	5403490.333	[3;6;0;0;0;0]
74	9300739023	[3;6;0;0;1;0]
74	8716746601	[3;6;0;0;1;2]
74	14336961020	[3;6;0;0;2;0]
74	3302393455	[3;6;0;0;2;2]
74	3798913202	[3;6;0;0;3;0]
74	10067520.96	[3;6;0;0;4;0]
74	6434456.229	[3;6;1;0;0;1]
74	3320086007	[3;6;1;0;1;0]
74	751040434.6	[3;6;1;0;1;2]
74	1542531293	[3;6;1;0;2;0]
74	3185511003	[3;7;0;0;1;0]
74	618753002.7	[3;7;0;0;1;2]
74	1339536426	[3;7;0;0;2;0]
74	515558012	[3;7;0;0;2;2]
74	102118893.7	[3;7;0;0;3;0]
74	696922755	[3;7;1;0;1;0]
74	164743110.5	[3;7;1;0;1;2]
74	1270510141	[3;8;0;0;1;0]
74	436851335.8	[3;8;0;0;1;2]
74	717496363.8	[3;8;0;0;2;0]

74	276171194.2	[3;8;0;0;2;2]
74	94716723.1	[3;8;0;0;3;0]
74	239400778.7	[3;8;1;0;1;0]
74	16572968.33	[4;2;0;0;1;0]
74	797369846.4	[4;3;0;0;1;0]
74	102088685.1	[4;3;0;0;2;0]
74	228655398.6	[4;3;1;0;1;0]
74	3146749156	[4;4;0;0;1;0]
74	433777594.2	[4;4;0;0;1;2]
74	2650108785	[4;4;0;0;2;0]
74	38381833.03	[4;4;0;0;3;0]
74	1971835352	[4;4;1;0;1;0]
74	23186027.21	[4;4;1;0;1;1]
74	14677454367	[4;5;0;0;1;0]
74	4881298287	[4;5;0;0;1;2]
74	24811786893	[4;5;0;0;2;0]
74	2365650892	[4;5;0;0;2;2]
74	6015059799	[4;5;0;0;3;0]
74	202056245.2	[4;5;0;0;3;2]
74	365792482.8	[4;5;0;0;4;0]
74	11369871339	[4;5;1;0;1;0]
74	12948185128	[4;5;1;0;1;2]
74	20020879976	[4;5;1;0;2;0]
74	70732998.1	[4;5;1;0;2;2]
74	131346866.6	[4;5;1;0;3;0]
74	7592247144	[4;5;2;0;1;0]
74	16284228.08	[4;6;0;0;0;0]
74	2317096088	[4;6;0;0;1;0]
74	1072836334	[4;6;0;0;2;0]
74	147512438.2	[4;6;0;0;3;0]
74	1431486878	[4;6;1;0;1;0]
74	322056697.4	[4;6;1;0;1;2]
74	454908696.1	[4;6;1;0;2;0]
74	406047538.8	[4;6;2;0;1;0]
74	2541616363	[4;7;0;0;1;0]
74	637579217.3	[4;7;0;0;1;2]
74	2130447315	[4;7;0;0;2;0]
74	359062560.8	[4;7;0;0;2;1]
74	614843890.8	[4;7;0;0;3;0]

74	2034053897	[4;7;1;0;1;0]
74	1413537266	[4;7;1;0;1;2]
74	1663598965	[4;7;1;0;2;0]
74	689222804.5	[4;7;1;0;2;2]
74	141320146.3	[4;7;1;0;3;0]
74	878545185.3	[4;7;2;0;1;0]
74	372400085.3	[4;7;2;0;1;2]
74	34623827.17	[4;8;0;0;1;0]
74	217036935.5	[5;2;0;0;0;0]
74	6611242.021	[5;2;0;0;0;2]
74	9173313.24	[5;2;0;0;1;0]
74	219236213.4	[5;2;0;0;3;0]
74	99966622.17	[5;3;0;0;3;0]
74	830648213.5	[5;4;0;0;1;0]
74	147776032.5	[5;4;0;0;1;1]
74	1030659174	[5;4;0;0;2;0]
74	174026470.9	[5;4;0;0;3;0]
74	2021613505	[5;4;1;0;1;0]
74	13326338.67	[5;4;1;0;1;1]
74	454547067.9	[5;4;1;0;2;0]
74	2233669613	[5;4;2;0;1;0]
74	49449829.04	[5;4;2;0;1;2]
74	3286321244	[5;5;0;0;1;0]
74	1413100947	[5;5;0;0;2;0]
74	168274951.5	[5;5;0;0;3;0]
74	1831998509	[5;5;1;0;1;0]
74	295371615.1	[5;5;1;0;2;0]
74	592596777.8	[5;5;2;0;1;0]
74	1392713783	[5;6;0;0;1;0]
74	94828401.29	[5;6;0;0;1;2]
74	1488264074	[5;6;0;0;2;0]
74	430451533.6	[5;6;0;0;3;0]
74	2198003463	[5;6;1;0;1;0]
74	846823539.1	[5;6;1;0;1;2]
74	1398270672	[5;6;1;0;2;0]
74	337172495.2	[5;6;1;0;3;0]
74	13378461.33	[5;6;1;0;4;0]
74	2108655497	[5;6;2;0;1;0]
74	1050114037	[5;6;2;0;1;2]

74	956822795.6	[5;6;2;0;2;0]
74	1485643622	[5;6;3;0;1;0]
74	41980028.61	[5;7;0;0;2;0]
74	348967285	[5;7;1;0;1;0]
74	204204049.3	[5;8;1;0;1;0]
74	154138721	[5;8;2;0;1;0]
74	190199814.3	[6;2;0;0;0;1]
74	294412175.8	[6;2;0;0;4;0]
74	144069863.8	[6;3;0;0;0;1]
74	15269386.24	[6;3;0;0;1;1]
74	107116092.1	[6;5;0;0;2;0]
74	732340028.1	[6;5;1;0;1;0]
74	267054827	[6;5;1;0;2;0]
74	596323937.5	[6;5;2;0;1;0]
74	1422229988	[6;5;3;0;1;0]
74	296709614.8	[6;6;0;0;1;0]
74	63352146.48	[6;6;0;0;2;0]
74	513180967.5	[6;6;1;0;1;0]
74	52523865.21	[6;6;1;0;2;0]
74	350574139.3	[6;6;2;0;1;0]
74	129255633.3	[6;7;0;0;1;0]
74	395778449.3	[6;7;1;0;1;0]
74	1322260279	[6;7;2;0;1;0]
74	22290705.33	[6;7;2;0;3;2]
74	542692255.2	[6;7;3;0;1;0]
74	62082975.67	[7;6;0;0;1;0]
74	326449208.1	[7;6;2;0;1;0]
74	619542963.1	[7;6;3;0;1;0]
74	51865988	[7;6;4;0;1;0]
74	209157742	[7;7;1;0;1;0]
74	49221604.71	[7;7;1;0;2;0]
74	70006806.88	[7;7;2;0;1;0]
74	9803628.365	[8;2;0;0;0;0]
74	165543748.2	[8;4;0;0;1;0]

Watanabe et al GlycReSoft Search

Site	Intensity	Glycan
122	9551546760	[3;3;0;0;0;0]
122	5886958730	[3;3;0;0;1;0]
122	6939061483	[3;4;0;0;0;0]
122	11561720289	[3;4;0;0;1;0]
122	3027862007	[3;5;0;0;0;0]
122	4441783721	[3;5;0;0;1;0]
122	602544346.1	[3;5;0;0;2;0]
122	373605916.7	[3;5;1;0;1;0]
122	38526125.63	[3;6;0;0;0;0]
122	589638416	[4;2;0;0;0;0]
122	14019078382	[4;3;0;0;0;0]
122	9327033140	[4;3;0;0;1;0]
122	457038268.4	[4;3;0;0;2;0]
122	2432760257	[4;3;1;0;0;0]
122	3033961593	[4;3;1;0;1;0]
122	8911464963	[4;4;0;0;0;0]
122	7754483379	[4;4;0;0;1;0]
122	20354124.29	[4;4;0;0;1;2]
122	1298518612	[4;4;0;0;2;0]
122	43535066.17	[4;4;0;0;3;0]
122	6626787264	[4;4;1;0;0;0]
122	14693497362	[4;4;1;0;1;0]
122	1645496440	[4;5;0;0;0;0]
122	1872309191	[4;5;0;0;1;0]
122	73969622.58	[4;5;0;0;1;2]
122	853067221.7	[4;5;0;0;2;0]
122	146938855.8	[4;5;1;0;0;0]
122	659398570.3	[4;5;1;0;1;0]
122	225415633.4	[4;7;0;0;5;0]
122	57042294658	[5;2;0;0;0;0]
122	27181432884	[5;3;0;0;0;0]
122	588087.0104	[5;3;0;0;0;2]
122	5259395453	[5;3;0;0;1;0]
122	723158365.7	[5;3;0;0;2;0]
122	7003885.333	[5;3;0;0;3;0]
122	12887978536	[5;3;1;0;0;0]
122	5964391573	[5;3;1;0;1;0]
122	2609743226	[5;4;0;0;0;0]
122	1854773522	[5;4;0;0;1;0]
122	699975646.7	[5;4;0;0;2;0]
122	2541062216	[5;4;1;0;0;0]
122	4795849783	[5;4;1;0;1;0]

122	221208990	[5;5;0;0;0;0]
122	293776897.9	[5;5;0;0;1;0]
122	105545981.9	[5;5;1;0;1;0]
122	2417876249	[6;2;0;0;0;0]
122	6017540225	[6;3;0;0;0;0]
122	1820174739	[6;3;0;0;1;0]
122	78902090.17	[6;3;0;0;2;0]
122	5455564854	[6;3;1;0;0;0]
122	90212074.48	[6;4;0;0;0;0]
122	175259514.3	[6;5;1;0;1;0]
122	2948923798	[7;2;0;0;0;0]
122	194593187.7	[8;2;0;0;0;0]
122	21368107.33	[9;2;0;0;0;0]

Watanabe et al GlycReSoft Search

Site	Intensity	Glycan
149	3000839634	[3;3;0;0;1;0]
149	19397745193	[3;4;0;0;1;0]
149	305028746.2	[3;4;0;0;2;0]
149	12543436551	[3;5;0;0;1;0]
149	18886434708	[3;5;0;0;2;0]
149	222036711.2	[3;5;0;0;5;0]
149	2901273949	[3;5;1;0;1;0]
149	1477210746	[3;6;0;0;1;0]
149	634333153.9	[3;6;0;0;2;0]
149	509032078.2	[3;6;1;0;1;0]
149	6945544419	[4;4;0;0;1;0]
149	2334065294	[4;4;0;0;2;0]
149	2327442575	[4;4;1;0;1;0]
149	18877731411	[4;5;0;0;1;0]
149	6733094646	[4;5;0;0;2;0]
149	431378178.7	[4;5;0;0;3;0]
149	2962693688	[4;5;1;0;1;0]
149	679572490.7	[4;5;1;0;2;0]
149	699733944.3	[4;5;2;0;1;0]
149	975750262	[5;4;0;0;2;0]
149	4354370532	[5;4;1;0;1;0]
149	1815616187	[5;5;0;0;1;0]

Watanabe et al GlycReSoft Search

Site	Intensity	Glycan
165	289940840.5	[3;3;0;0;0;0]
165	133837149.8	[3;3;0;0;1;0]
165	869783700.8	[3;4;0;0;0;0]
165	1585162127	[3;4;0;0;1;0]
165	901769566.1	[3;5;0;0;0;0]
165	2018047876	[3;5;0;0;1;0]
165	101931118.6	[3;5;0;0;2;0]
165	232793152	[3;6;0;0;1;0]
165	106700817.6	[4;2;0;0;0;0]
165	219421469	[4;3;0;0;0;0]
165	113225479	[4;3;0;0;1;0]
165	226539984.5	[4;3;1;0;0;0]
165	360251341.2	[4;4;0;0;0;0]
165	851619809.6	[4;4;0;0;1;0]
165	165117906.5	[4;4;0;0;2;0]
165	91443214.35	[4;4;1;0;0;0]
165	259706061.6	[4;4;1;0;1;0]
165	1052684571	[4;5;0;0;0;0]
165	2928165910	[4;5;0;0;1;0]
165	491160885.3	[4;5;0;0;2;0]
165	314411887.6	[4;5;1;0;1;0]
165	24139866.67	[4;6;0;0;0;0]
165	211215238.7	[4;6;0;0;1;0]
165	9243574614	[5;2;0;0;0;0]
165	57473916	[5;3;0;0;0;0]
165	72593366.21	[5;3;0;0;1;0]
165	172071476.5	[5;3;1;0;0;0]
165	655434821.4	[5;4;0;0;0;0]
165	1431983412	[5;4;0;0;1;0]
165	668029454.5	[5;4;0;0;2;0]
165	138453687.1	[5;4;0;0;3;0]
165	878234052.2	[5;4;1;0;0;0]
165	2082977826	[5;4;1;0;1;0]
165	240007063.9	[5;4;1;0;2;0]
165	471217692.6	[5;4;2;0;0;0]
165	1337838523	[5;4;2;0;1;0]
165	310270627.1	[5;5;0;0;0;0]

165	857041718.5	[5;5;0;0;1;0]
165	141897054.2	[5;5;0;0;2;0]
165	311889062.2	[5;6;0;0;1;0]
165	374085021.5	[6;2;0;0;0;0]
165	215125161.8	[6;3;0;0;0;0]
165	160247302.6	[6;3;1;0;0;0]
165	77534719.88	[6;4;0;0;2;0]
165	132668660.2	[6;5;0;0;0;0]
165	521764123.6	[6;5;0;0;1;0]
165	188866403.9	[6;5;0;0;2;0]
165	1035809964	[6;5;1;0;1;0]
165	809569737.2	[6;5;2;0;1;0]
165	41930066.21	[6;5;3;0;0;0]

Watanabe et al GlycReSoft Search

Site	Intensity	Glycan
234	564066979.7	[3;3;0;0;0;0]
234	396155902.3	[3;3;0;0;1;0]
234	1070183453	[3;4;0;0;0;0]
234	2734414216	[3;4;0;0;1;0]
234	98009182.5	[3;4;0;0;4;0]
234	3888051428	[3;4;1;0;2;0]
234	800199493.3	[3;5;0;0;1;0]
234	595873513.6	[3;6;0;0;2;0]
234	2493711.875	[3;7;0;0;5;0]
234	2702101	[4;2;0;0;4;1]
234	1117383883	[4;3;0;0;0;0]
234	135481397.1	[4;3;0;0;1;0]
234	118040591.2	[4;4;0;0;1;0]
234	122749284.4	[4;5;0;0;1;0]
234	12368494010	[5;2;0;0;0;0]
234	2487629050	[5;3;0;0;0;0]
234	79195209.92	[5;3;0;0;1;0]
234	328540664.9	[5;4;0;0;4;0]
234	11353265851	[6;2;0;0;0;0]
234	427414278.7	[6;3;0;0;0;0]
234	20899599.17	[6;7;0;0;1;0]
234	41098807143	[7;2;0;0;0;0]
234	111535673876	[8;2;0;0;0;0]
234	86270311128	[9;2;0;0;0;0]
234	1259913492	[9;6;1;0;3;0]

Watanabe et al GlycReSoft Search

Site	Intensity	Glycan
282	1730719858	[3;3;0;0;0;0]
282	1113738060	[3;4;0;0;0;0]
282	1333514735	[3;4;0;0;1;0]
282	2134818907	[3;5;0;0;0;0]
282	12144322859	[3;5;0;0;1;0]
282	533449526.8	[3;6;0;0;0;0]
282	748537074.3	[3;6;0;0;1;0]
282	9293188.333	[3;7;1;0;3;1]
282	75171250	[4;3;0;0;0;0]
282	300301277	[4;4;0;0;0;0]
282	1750756714	[4;4;0;0;1;0]
282	124207147.3	[4;4;1;0;1;0]
282	1033343473	[4;5;0;0;0;0]
282	5490647750	[4;5;0;0;1;0]
282	250868173.7	[4;5;1;0;0;0]
282	330607633.4	[4;5;1;0;1;0]
282	1045661401	[4;6;0;0;1;0]
282	165575584.8	[5;2;0;0;0;0]
282	2165245062	[5;4;0;0;1;0]
282	149167003.5	[5;4;0;0;2;0]
282	243043470.1	[5;4;1;0;1;0]
282	535499388.8	[5;5;0;0;0;0]
282	2527908159	[5;5;0;0;1;0]
282	201654019.6	[5;5;1;0;0;0]
282	533694405.8	[5;5;1;0;1;0]
282	145358121	[5;6;0;0;0;0]
282	883179659.8	[5;6;0;0;1;0]
282	628328716.1	[6;5;0;0;0;0]
282	5133185330	[6;5;0;0;1;0]
282	164644066.3	[6;5;0;0;2;0]
282	277029344.2	[6;5;1;0;0;0]
282	929178441.9	[6;5;1;0;1;0]
282	89980875.06	[6;5;2;0;0;0]
282	127611059.2	[7;6;0;0;1;0]

Watanabe et al GlycReSoft Search

Site	Intensity	Glycan

331	5656012096	[3;3;0;0;1;0]
331	87292889340	[3;4;0;0;1;0]
331	80439631599	[3;5;0;0;1;0]
331	395673919.9	[3;5;0;0;2;0]
331	1288918652	[3;5;1;0;1;0]
331	1117264155	[3;6;0;0;1;0]
331	29105254751	[4;4;0;0;1;0]
331	2624421370	[4;4;0;0;2;0]
331	61091518672	[4;4;1;0;1;0]
331	45173793988	[4;5;0;0;1;0]
331	983649032	[4;5;0;0;2;0]
331	21052118126	[4;5;1;0;1;0]
331	3133254971	[5;2;0;0;0;0]
331	18709208352	[5;4;0;0;1;0]
331	1910033045	[5;4;0;0;2;0]
331	72065986911	[5;4;1;0;1;0]
331	3398350398	[5;4;2;0;1;0]
331	13970584481	[5;5;0;0;1;0]
331	3630054180	[5;5;1;0;1;0]
331	559620718.4	[5;6;0;0;1;0]
331	709347271.6	[6;5;0;0;1;0]
331	12220771941	[6;5;1;0;1;0]
331	880201635.8	[6;5;2;0;1;0]

Watanabe et al GlycReSoft Search

Site	Intensity	Glycan
331,343	77496574.17	[10;6;0;0;1;0]
331,343	187492475.9	[10;6;1;0;1;0]
331,343	708165616.2	[6;7;0;0;2;0]
331,343	3220302393	[6;8;0;0;2;0]
331,343	274566250	[7;7;0;0;2;0]
331,343	922554585.1	[7;8;0;0;2;0]
331,343	1297272334	[7;8;1;0;2;0]
331,343	278172764.4	[8;5;0;0;1;0]
331,343	1123163816	[8;6;0;0;1;0]
331,343	361478135	[8;7;0;0;1;0]
331,343	540697293.3	[8;8;0;0;2;0]
331,343	1637144417	[8;8;1;0;2;0]
331,343	267588365	[9;6;0;0;1;0]
331,343	263846293	[9;6;1;0;1;0]

Site	Intensity	Glycan
343	1715446040	[3;4;0;0;1;0]
343	1753206654	[3;5;0;0;1;0]
343	206828363.8	[4;3;0;0;1;0]
343	1282489004	[4;4;0;0;1;0]
343	529411055.8	[4;4;1;0;1;0]
343	2628668749	[4;5;0;0;1;0]
343	669393652.8	[5;2;0;0;0;0]
343	267416083.1	[5;3;0;0;1;0]
343	3897038379	[5;4;0;0;1;0]
343	218083434	[5;4;0;0;2;0]
343	1008474886	[5;4;1;0;1;0]
343	815369533.4	[5;5;0;0;1;0]

Watanabe et al GlycReSoft Search

Site	Intensity	Glycan
657	1599479286	[3;3;0;0;1;0]
657	14036775048	[3;4;0;0;1;0]
657	11126391156	[3;5;0;0;1;0]
657	2659112173	[4;4;0;0;1;0]
657	4769183285	[4;4;1;0;1;0]
657	3751842235	[4;5;0;0;1;0]
657	1218421973	[5;2;0;0;0;0]
657	4124111576	[5;4;0;0;1;0]
657	8539770458	[5;4;1;0;1;0]
657	5141382728	[5;5;0;0;1;0]

Watanabe et al GlycReSoft Search

Site	Intensity	Glycan
709,717	4886071956	[10;4;0;0;0;0]
709,717	899811810.7	[10;4;0;0;5;1]
709,717	19150364.75	[10;5;0;0;1;0]
709,717	4642096880	[10;6;0;0;0;1]
709,717	434695110.4	[10;6;0;0;1;0]
709,717	11004295785	[8;6;0;0;0;1]
709,717	2023007891	[8;7;0;0;0;1]
709,717	139234559.3	[8;7;0;0;1;0]
709,717	2320950059	[9;4;0;0;5;1]
709,717	301230898.3	[9;5;0;0;0;0]
709,717	4376755125	[9;6;0;0;0;1]
709,717	101777876.3	[9;6;0;0;1;0]

709,717	705225001.8	[9;7;0;0;0;1]
709,717	238801196.3	[9;7;0;0;1;0]

Watanabe et al GlycReSoft Search

Site	Intensity	Glycan
801	1578285855	[3;2;0;0;0;0]
801	51244539.33	[3;2;0;0;4;1]
801	1896037591	[3;3;0;0;0;0]
801	305427877.2	[3;3;0;0;1;0]
801	5686501841	[3;4;0;0;0;0]
801	9799764008	[3;4;0;0;1;0]
801	2950372207	[3;5;0;0;0;0]
801	5172585844	[3;5;0;0;1;0]
801	722270326.6	[3;5;0;0;3;0]
801	6974386645	[4;2;0;0;0;0]
801	11422356598	[4;3;0;0;0;0]
801	389455585.9	[4;3;0;0;1;0]
801	536030096	[4;3;1;0;0;0]
801	2085453864	[4;4;0;0;0;0]
801	4524321705	[4;4;0;0;1;0]
801	275958142.8	[4;4;0;0;2;0]
801	3471867623	[4;4;1;0;0;0]
801	14981222911	[4;4;1;0;1;0]
801	124206748.3	[4;5;0;0;0;0]
801	1595450658	[4;5;0;0;1;0]
801	371524754.2	[4;5;0;0;3;0]
801	96470745.38	[4;5;1;0;1;0]
801	10724168401	[4;8;1;0;0;0]
801	291401167616	[5;2;0;0;0;0]
801	20579034730	[5;3;0;0;0;0]
801	907158581.2	[5;3;0;0;1;0]
801	7639943797	[5;3;0;0;2;0]
801	9925104502	[5;3;1;0;0;0]
801	1538564343	[5;3;1;0;1;0]
801	1702271430	[5;4;0;0;0;0]
801	2162734130	[5;4;0;0;1;0]
801	172444283.7	[5;4;0;0;2;0]
801	2224110334	[5;4;1;0;0;0]
801	4397807552	[5;4;1;0;1;0]
801	161679337.1	[5;4;2;0;1;0]
801	71462024.23	[5;5;0;0;1;0]
801	39835435.38	[5;5;1;0;1;0]
801	19758001568	[6;2;0;0;0;0]
801	15690448921	[6;3;0;0;0;0]
801	457546207.1	[6;3;0;0;1;0]
801	10560649046	[6;3;1;0;0;0]
801	6255290.333	[6;4;0;0;0;0]
801	27151940835	[7;2;0;0;0;0]
801	68496161.99	[7;3;0;0;0;1]
801	4830314503	[8;2;0;0;0;0]
801	123426832.3	[9;4;0;0;2;0]
801	92391038.58	[9;6;0;0;5;1]

Watanabe et al GlycReSoft Search

Site	Intensity	Glycan
1074	566613579.9	[3;2;0;0;0;0]
1074	2904386787	[3;3;0;0;0;0]
1074	21326710301	[3;3;0;0;1;0]
1074	315684.9427	[3;3;0;0;4;2]
1074	1988822447	[3;4;0;0;0;0]
1074	62573676194	[3;4;0;0;1;0]
1074	962386561	[3;5;0;0;0;0]
1074	74068677853	[3;5;0;0;1;0]
1074	10179849817	[3;6;0;0;1;0]
1074	311495478.2	[3;7;0;0;1;0]
1074	2123169457	[4;2;0;0;0;0]
1074	10408890331	[4;3;0;0;0;0]
1074	18706454592	[4;3;0;0;1;0]
1074	3510819.24	[4;3;0;0;1;1]
1074	99682711.47	[4;3;0;0;2;0]
1074	200125261.2	[4;3;1;0;0;0]
1074	1880776433	[4;3;1;0;1;0]
1074	1498425377	[4;4;0;0;0;0]
1074	34047117200	[4;4;0;0;1;0]
1074	92618815.22	[4;4;0;0;2;0]
1074	173829557	[4;4;1;0;0;0]
1074	4682840958	[4;4;1;0;1;0]
1074	176414.8594	[4;4;2;0;4;0]
1074	415752984.2	[4;5;0;0;0;0]
1074	24258032057	[4;5;0;0;1;0]

1074	1714113016	[4;5;1;0;1;0]
1074	2301025068	[4;6;0;0;1;0]
1074	670094396.2	[4;6;1;0;1;0]
1074	37795526.92	[4;7;1;0;1;0]
1074	154307726182	[5;2;0;0;0;0]
1074	21121515993	[5;3;0;0;0;0]
1074	17713134508	[5;3;0;0;1;0]
1074	82722883.5	[5;3;0;0;2;0]
1074	243222334.6	[5;3;0;0;3;0]
1074	1274257258	[5;3;1;0;0;0]
1074	2860423088	[5;3;1;0;1;0]
1074	1926439475	[5;4;0;0;0;0]
1074	30733770345	[5;4;0;0;1;0]
1074	213806008.4	[5;4;0;0;2;0]
1074	304025619.6	[5;4;1;0;0;0]
1074	5933752733	[5;4;1;0;1;0]
1074	24318809.79	[5;4;1;0;2;0]
1074	222850363.5	[5;4;2;0;1;0]
1074	190180258.9	[5;5;0;0;0;0]
1074	11867165529	[5;5;0;0;1;0]
1074	2160860413	[5;5;1;0;1;0]
1074	134140425.3	[5;5;2;0;1;0]
1074	1674146636	[5;6;0;0;1;0]
1074	2698389.917	[5;6;0;0;2;0]
1074	747963900.5	[5;6;1;0;1;0]
1074	72682503.6	[5;6;2;0;1;0]
1074	110086756.3	[5;7;0;0;1;0]
1074	20528234.18	[5;7;1;0;1;0]
1074	2612789101	[6;2;0;0;0;0]
1074	3918478519	[6;3;0;0;0;0]
1074	1815110398	[6;3;0;0;1;0]
1074	2572756521	[6;3;1;0;0;0]
1074	277156165.3	[6;3;1;0;1;0]
1074	29552373.9	[6;4;0;0;0;0]
1074	21777014.43	[6;4;0;0;1;0]
1074	185628440	[6;5;0;0;0;0]
1074	7559610434	[6;5;0;0;1;0]
1074	151309522.1	[6;5;0;0;2;0]
1074	42102319.4	[6;5;1;0;0;0]
1074	2649622940	[6;5;1;0;1;0]
1074	179571519.3	[6;5;2;0;1;0]
1074	1203515200	[6;6;0;0;1;0]
1074	737184486.6	[6;6;1;0;1;0]
1074	24295471.42	[6;6;2;0;1;0]
1074	171819981.4	[6;7;0;0;1;0]
1074	778626343.9	[7;2;0;0;0;0]
1074	1327777.734	[7;2;0;0;2;0]
1074	125757924.1	[7;4;0;0;4;0]
1074	1074559893	[7;6;0;0;1;0]
1074	40311329.52	[7;6;0;0;2;0]
1074	1483969100	[7;6;1;0;1;0]
1074	402451764.5	[7;6;2;0;1;0]
1074	75211505.97	[7;7;0;0;1;0]
1074	506746880.7	[8;2;0;0;0;0]
1074	50957688.18	[9;2;0;0;0;0]

Watanabe et al GlycReSoft Search

Site	Intensity	Glycan
1098	4173275.5	[3;2;0;0;2;1]
1098	106591916.8	[3;2;0;0;3;0]
1098	92587762.67	[3;2;0;0;4;0]
1098	5106480495	[3;3;0;0;0;0]
1098	3098420059	[3;3;0;0;1;0]
1098	7777355225	[3;4;0;0;0;0]
1098	10930097342	[3;4;0;0;1;0]
1098	6432307.625	[3;4;0;0;1;2]
1098	4160314719	[3;5;0;0;0;0]
1098	3344478547	[3;5;0;0;1;0]
1098	2083693756	[3;6;0;0;0;0]
1098	1417889611	[3;6;0;0;1;0]
1098	19773899.23	[3;7;0;0;0;0]
1098	20295238.75	[4;2;0;0;0;0]
1098	6701972240	[4;3;0;0;0;0]
1098	7548577365	[4;3;0;0;1;0]
1098	48217070.65	[4;3;0;0;1;1]
1098	1494836706	[4;3;1;0;0;0]
1098	1039077467	[4;3;1;0;1;0]
1098	14422925.13	[4;3;1;0;3;0]
1098	3780587081	[4;4;0;0;0;0]

1098	2659008415	[4;4;0;0;1;0]
1098	74943536.42	[4;4;0;0;2;0]
1098	3393794.167	[4;4;0;0;3;0]
1098	7338083379	[4;4;1;0;0;0]
1098	10996540621	[4;4;1;0;1;0]
1098	1275693111	[4;5;0;0;0;0]
1098	759949903.9	[4;5;0;0;1;0]
1098	2397172141	[4;5;1;0;0;0]
1098	3809337004	[4;5;1;0;1;0]
1098	559471595.4	[4;6;0;0;0;0]
1098	485849580.5	[4;6;0;0;1;0]
1098	2432592355	[4;6;1;0;0;0]
1098	3830970498	[4;6;1;0;1;0]
1098	4077951956	[5;2;0;0;0;0]
1098	20435505210	[5;3;0;0;0;0]
1098	5746357957	[5;3;0;0;1;0]
1098	71465565.67	[5;3;0;0;2;0]
1098	3666242863	[5;3;1;0;0;0]
1098	3823578768	[5;3;1;0;1;0]
1098	907950109.1	[5;4;0;0;0;0]
1098	406334476	[5;4;0;0;1;0]
1098	8464510064	[5;4;1;0;0;0]
1098	9614112442	[5;4;1;0;1;0]
1098	237327520.1	[5;4;2;0;0;0]
1098	222069061.3	[5;4;2;0;1;0]
1098	434650756.7	[5;5;0;0;0;0]
1098	243668412.5	[5;5;0;0;1;0]
1098	12406866.67	[5;5;0;0;3;0]
1098	3095854609	[5;5;1;0;0;0]
1098	3107295063	[5;5;1;0;1;0]
1098	1103739490	[5;5;2;0;0;0]
1098	439839994.9	[5;5;2;0;1;0]
1098	309700257	[5;6;0;0;0;0]
1098	256214396.2	[5;6;0;0;1;0]
1098	2272780168	[5;6;1;0;0;0]
1098	2057546912	[5;6;1;0;1;0]
1098	526002147.7	[5;6;2;0;0;0]
1098	78910981.67	[5;6;2;0;1;0]
1098	1002015253	[6;2;0;0;0;0]
1098	5450969848	[6;3;0;0;0;0]
1098	640400333.4	[6;3;0;0;1;0]
1098	9631900.875	[6;3;0;0;2;0]
1098	4383758601	[6;3;1;0;0;0]
1098	1066417428	[6;3;1;0;1;0]
1098	145794259.7	[6;4;0;0;0;0]
1098	28130130.25	[6;4;0;0;1;0]
1098	140486358.9	[6;4;1;0;0;0]
1098	78469150.13	[6;4;1;0;1;0]
1098	387310352.2	[6;5;0;0;0;0]
1098	668948230.1	[6;5;0;0;1;0]
1098	3005588764	[6;5;1;0;0;0]
1098	3284396051	[6;5;1;0;1;0]
1098	6502162	[6;5;1;0;2;0]
1098	1106881735	[6;5;2;0;0;0]
1098	351897569.4	[6;5;2;0;1;0]
1098	475333998.1	[6;6;0;0;0;0]
1098	110033308.1	[6;6;0;0;1;0]
1098	2029715777	[6;6;1;0;0;0]
1098	1837115959	[6;6;1;0;1;0]
1098	104268622	[6;6;1;0;2;0]
1098	99788409.08	[6;6;2;0;0;0]
1098	11892127	[6;6;2;0;1;0]
1098	1095816758	[7;2;0;0;0;0]
1098	446250296.8	[7;3;0;0;0;0]
1098	391621873.4	[7;3;1;0;0;0]
1098	15906050.08	[7;4;1;0;0;0]
1098	540739631.9	[7;6;0;0;0;0]
1098	1928289318	[7;6;1;0;0;0]
1098	2296453677	[7;6;1;0;1;0]
1098	521451386.2	[7;6;2;0;0;0]
1098	399743822.4	[7;6;2;0;1;0]
1098	47143958.17	[7;6;3;0;0;0]
1098	114776897.1	[8;2;0;0;0;0]
1098	14481292.42	[9;2;0;0;0;0]

Watanabe et al GlycReSoft Search

Site	Intensity	Glycan
1194	57692395	[3;4;2;0;4;0]
1194	284098197.4	[3;6;0;0;1;0]

1194	166668954	[4;6;0;0;1;0]
1194	177029134	[4;6;1;0;1;0]
1194	95913610.22	[5;2;0;0;0;0]
1194	392656.375	[5;6;1;0;1;0]
1194	178554983.4	[5;6;2;0;1;0]
1194	40679258.83	[5;6;2;0;1;2]
1194	109203897.9	[6;6;2;0;1;0]
1194	226528852.4	[6;6;3;0;1;0]
1194	104818270.9	[6;7;3;0;1;0]
1194	47699842	[7;6;2;0;1;0]
1194	70674686.6	[7;6;3;0;1;0]

Figure S4c. Watanabe et al search using MSFragger-Glyco

Site	AUC	Glycan
17	2170451710	[6;2;2;0;1;0]

Watanabe et al MSFragger-Glyco Search

Site	AUC	Glycan
61	5621988400	[3;3;0;0;0;0]
61	4714395415	[3;4;0;0;0;0]
61	413759264	[3;4;0;0;1;0]
61	1920567793	[3;5;0;0;0;0]
61	438399648	[3;5;0;0;1;0]
61	906116670	[3;6;0;0;0;0]
61	416076416	[4;2;0;0;0;0]
61	6666367500	[4;3;0;0;0;0]
61	1178490584	[4;3;0;0;1;0]
61	230590120	[4;3;1;0;0;0]
61	2707488710	[4;4;0;0;0;0]
61	548666166.3	[4;4;0;0;1;0]
61	967901695	[4;4;1;0;0;0]
61	2549925880	[4;5;0;0;0;0]
61	51597720	[4;5;1;0;0;0]
61	604105260	[4;6;0;0;0;0]
61	93734093000	[5;2;0;0;0;0]
61	3631273973	[5;3;0;0;0;0]
61	782715647	[5;3;0;0;1;0]
61	349597944	[5;3;1;0;0;0]
61	3000104544	[5;4;0;0;0;0]
61	1236197082	[5;4;0;0;1;0]
61	1197079300	[5;4;0;0;2;0]
61	3383111170	[5;4;1;0;0;0]
61	777912106.7	[5;4;1;0;1;0]
61	1411182080	[5;4;1;0;2;0]
61	1506733570	[5;4;2;0;0;0]
61	3269671680	[5;5;0;0;0;0]
61	111860560	[5;5;0;0;1;0]
61	68938048	[5;5;1;0;0;0]
61	503389312	[5;6;0;0;0;0]
61	2415220220	[6;2;0;0;0;0]
61	1539158763	[6;3;0;0;0;0]
61	44362960	[6;3;0;0;1;0]
61	298356080	[6;3;1;0;0;0]
61	68562304	[6;4;0;0;0;0]
61	35432692	[6;4;1;0;1;0]
61	732331135	[6;5;0;0;0;0]
61	744387805	[6;5;2;0;0;0]
61	1015347460	[6;6;0;0;0;0]
61	269558762.7	[7;2;0;0;0;0]

Watanabe et al MSFragger-Glyco Search

Site	AUC	Glycan
74	214144035.2	[0;1;0;0;1;0]
74	2002166563	[0;2;0;0;0;0]
74	290284704	[2;2;0;0;1;0]
74	129583313.6	[3;2;0;0;1;0]
74	1166072953	[3;3;0;0;1;0]
74	2636096206	[3;4;0;0;1;0]
74	323015104	[3;4;1;0;0;0]
74	20653400	[3;5;0;0;0;0]
74	5233447472	[3;5;0;0;1;0]
74	5141918779	[3;5;0;0;2;0]
74	2220758957	[3;5;1;0;1;0]
74	8614040	[3;6;0;0;0;0]
74	2191772163	[3;6;0;0;1;0]
74	2867636336	[3;6;0;0;2;0]
74	2056421741	[3;6;1;0;1;0]
74	127884832	[3;6;2;0;1;0]
74	1253898554	[3;7;0;0;1;0]
74	36037824	[3;8;0;0;0;0]
74	391424407.1	[3;8;0;0;1;0]
74	23708068	[3;9;0;0;1;0]
74	51959816	[4;2;0;0;1;0]
74	645759711.1	[4;3;0;0;1;0]
74	64108534	[4;3;0;0;2;0]
74	583148030	[4;3;1;0;1;0]
74	944349508.9	[4;4;0;0;1;0]
74	994608446.9	[4;4;0;0;2;0]
74	23111975	[4;4;1;0;0;0]
74	684117392.9	[4;4;1;0;1;0]

74	17390492	[4;5;0;0;0;0]
74	2037390029	[4;5;0;0;1;0]
74	5375617798	[4;5;0;0;2;0]
74	2936605014	[4;5;1;0;0;0]
74	2403594753	[4;5;1;0;1;0]
74	203306464	[4;5;2;0;0;0]
74	2091291649	[4;5;2;0;1;0]
74	8259899	[4;6;0;0;0;0]
74	1154903105	[4;6;0;0;1;0]
74	271091730.3	[4;6;0;0;2;0]
74	73658890.67	[4;6;1;0;0;0]
74	982557578.9	[4;7;0;0;1;0]
74	454895520	[5;2;0;0;0;0]
74	35480176	[5;2;0;0;1;0]
74	14636628	[5;3;0;0;1;0]
74	281500390	[5;4;0;0;1;0]
74	544770908	[5;4;0;0;2;0]
74	311175200	[5;4;1;0;0;0]
74	720324001.9	[5;4;1;0;1;0]
74	260283585.8	[5;4;1;0;2;0]
74	74202058	[5;4;1;0;3;0]
74	702439308.5	[5;4;2;0;1;0]
74	683070450.3	[5;5;0;0;1;0]
74	519486334	[5;5;0;0;2;0]
74	158820202.8	[5;5;0;0;3;0]
74	34993640	[5;5;1;0;0;0]
74	568294401.8	[5;5;1;0;1;0]
74	148636461.8	[5;5;1;0;2;0]
74	29546655	[5;5;2;0;0;0]
74	175514297.3	[5;5;2;0;1;0]
74	581163143.1	[5;6;0;0;1;0]
74	348272413.8	[5;6;0;0;2;0]
74	279236431.1	[5;6;0;0;3;0]
74	670048058	[5;6;1;0;1;0]
74	683832983.7	[5;6;1;0;2;0]
74	1248535771	[5;6;2;0;1;0]
74	727977092	[5;6;3;0;1;0]
74	657372136.7	[5;8;0;0;0;0]
74	53457536	[5;8;0;0;1;0]
74	47924040	[6;2;0;0;0;0]
74	556496122.7	[6;2;2;0;1;0]
74	18321189.5	[6;4;0;0;1;0]
74	20107419	[6;4;1;0;1;0]
74	35754416	[6;5;0;0;1;0]
74	67520506.5	[6;5;0;0;2;0]
74	44109833.64	[6;5;0;0;3;0]
74	593846293.5	[6;5;1;0;1;0]
74	202251659.8	[6;5;1;0;2;0]
74	37574369	[6;5;1;0;3;0]
74	79507836.8	[6;5;2;0;1;0]
74	797553357.6	[6;5;3;0;1;0]
74	199631874.3	[6;6;0;0;1;0]
74	42953009.31	[6;6;0;0;2;0]
74	228164094	[6;6;1;0;1;0]
74	62031542	[6;6;1;0;2;0]
74	88843184	[6;6;2;0;0;0]
74	78027320	[6;6;2;0;2;0]
74	2348074370	[6;7;0;0;0;0]
74	71013706	[6;7;0;0;1;0]
74	628954495	[6;9;0;0;0;0]
74	110973328	[7;2;0;0;0;0]
74	22099464	[7;4;0;0;1;0]
74	64906520	[7;5;2;0;1;0]
74	19667540	[7;6;0;0;0;0]
74	33564184	[7;6;0;0;3;0]
74	159867187	[7;6;1;0;1;0]
74	70315746	[7;6;1;0;2;0]
74	69238556	[7;6;1;0;3;0]
74	291641446.7	[7;6;2;0;1;0]
74	73323072	[7;7;0;0;1;0]
74	124613608	[7;7;3;0;1;0]
74	532480064	[7;8;0;0;0;0]
74	34482580	[8;2;0;0;0;0]
74	11127646	[8;7;0;0;0;0]
74	869209660	[8;7;1;0;0;0]

Watanabe et al MSFragger-Glyco Search

Site	AUC	Glycan
122	105972980	[0;1;0;0;0;0]

122	28200800	[3;2;0;0;0;0]
122	21982034	[3;2;0;0;1;0]
122	3480023530	[3;3;0;0;0;0]
122	4400846907	[3;3;0;0;1;0]
122	4918888071	[3;4;0;0;0;0]
122	6942582426	[3;4;0;0;1;0]
122	215316832	[3;4;1;0;0;0]
122	2760125554	[3;5;0;0;0;0]
122	3920176821	[3;5;0;0;1;0]
122	1549131968	[3;5;0;0;2;0]
122	386068959.3	[3;5;1;0;1;0]
122	174486272	[3;6;0;0;0;0]
122	325400768	[3;6;0;0;1;0]
122	136426284	[4;2;0;0;0;0]
122	10689602600	[4;3;0;0;0;0]
122	5739310794	[4;3;0;0;1;0]
122	628405953.7	[4;3;0;0;2;0]
122	1315886491	[4;3;1;0;0;0]
122	1824876974	[4;3;1;0;1;0]
122	298135008	[4;3;1;0;2;0]
122	4924866503	[4;4;0;0;0;0]
122	10648246812	[4;4;0;0;1;0]
122	3339719002	[4;4;0;0;2;0]
122	1759705145	[4;4;1;0;0;0]
122	3515513926	[4;4;1;0;1;0]
122	1376939318	[4;5;0;0;0;0]
122	1808726245	[4;5;0;0;1;0]
122	759662571.5	[4;5;0;0;2;0]
122	197525446.4	[4;5;1;0;0;0]
122	344485964	[4;5;1;0;1;0]
122	155014400	[4;6;0;0;1;0]
122	23863062610	[5;2;0;0;0;0]
122	1594635308	[5;2;1;0;1;0]
122	13375995027	[5;3;0;0;0;0]
122	4910782716	[5;3;0;0;1;0]
122	2937849634	[5;3;1;0;0;0]
122	2417912069	[5;4;0;0;0;0]
122	2555829356	[5;4;0;0;1;0]
122	450560705.3	[5;4;0;0;2;0]
122	1505076105	[5;4;1;0;0;0]
122	1905373463	[5;4;1;0;1;0]
122	537559178	[5;5;0;0;0;0]
122	687909846	[5;5;0;0;1;0]
122	208894504	[5;5;1;0;1;0]
122	245970672	[5;6;0;0;3;0]
122	2787794807	[6;2;0;0;0;0]
122	6431716726	[6;3;0;0;0;0]
122	2268023574	[6;3;0;0;1;0]
122	1787012979	[6;3;1;0;0;0]
122	346455760	[6;4;0;0;0;0]
122	57838544	[6;5;1;0;0;0]
122	158653120	[6;5;1;0;1;0]
122	4427646726	[7;2;0;0;0;0]
122	19171208	[7;4;1;0;0;0]
122	399792693	[8;2;0;0;0;0]
122	172591696	[9;2;0;0;0;0]

Watanabe et al MSFragger-Glyco Search

Site	AUC	Glycan
149	110282856	[0;1;0;0;1;0]
149	241574330.7	[3;2;0;0;1;0]
149	1037948941	[3;3;0;0;1;0]
149	7691622635	[3;4;0;0;1;0]
149	154887760	[3;5;0;0;0;0]
149	4663129540	[3;5;0;0;1;0]
149	4653221192	[3;5;0;0;2;0]
149	4542416234	[3;5;1;0;1;0]
149	137509568	[3;6;0;0;0;0]
149	960784046.9	[3;6;0;0;1;0]
149	1180550705	[3;6;0;0;2;0]
149	1171633768	[3;6;1;0;1;0]
149	6015364600	[3;6;2;0;1;0]
149	350119024	[3;7;0;0;1;0]
149	1648893823	[4;2;0;0;1;0]
149	443992005.4	[4;3;0;0;1;0]
149	57259464	[4;3;0;0;2;0]
149	44652828	[4;4;0;0;0;0]
149	3284235093	[4;4;0;0;1;0]
149	1287059126	[4;4;0;0;2;0]

149	1382527528	[4;4;1;0;1;0]
149	3332431139	[4;5;0;0;1;0]
149	3385035699	[4;5;0;0;2;0]
149	2178630178	[4;5;1;0;1;0]
149	1408999425	[4;5;2;0;1;0]
149	311374336	[4;6;0;0;1;0]
149	560633280	[4;6;0;0;2;0]
149	218861504	[4;7;0;0;1;0]
149	1803448705	[5;2;0;0;0;0]
149	155188992	[5;3;0;0;1;0]
149	1179375317	[5;4;0;0;1;0]
149	877706377	[5;4;0;0;2;0]
149	1756251187	[5;4;1;0;1;0]
149	212723200	[5;4;1;0;3;0]
149	497539248	[5;4;2;0;1;0]
149	1775740160	[5;5;0;0;1;0]
149	316056480	[5;5;0;0;2;0]
149	448537024	[5;5;1;0;1;0]
149	457059776	[5;5;1;0;2;0]
149	249210176	[5;5;2;0;1;0]
149	524769664.7	[5;6;1;0;1;0]
149	508638336	[5;6;1;0;2;0]
149	423312192	[6;5;2;0;1;0]
149	260352336	[6;6;1;0;1;0]

Watanabe et al MSFragger-Glyco Search

Site	AUC	Glycan
165	303664832	[0;1;0;0;0;0]
165	600365554	[3;3;0;0;0;0]
165	304547746.7	[3;3;0;0;1;0]
165	934787971.7	[3;4;0;0;0;0]
165	1905633364	[3;4;0;0;1;0]
165	758037054	[3;5;0;0;0;0]
165	1947158399	[3;5;0;0;1;0]
165	257316256	[3;5;0;0;2;0]
165	554792031	[3;6;0;0;1;0]
165	84577944	[3;7;0;0;1;0]
165	415183104	[4;2;0;0;0;0]
165	315989438.4	[4;3;0;0;0;0]
165	249509060	[4;3;0;0;1;0]
165	360857504	[4;3;1;0;0;0]
165	82981440	[4;3;1;0;1;0]
165	541403881.3	[4;4;0;0;0;0]
165	1152616219	[4;4;0;0;1;0]
165	197151360	[4;4;0;0;2;0]
165	114681480	[4;4;1;0;0;0]
165	354995935.3	[4;4;1;0;1;0]
165	1813962592	[4;5;0;0;0;0]
165	2431113681	[4;5;0;0;1;0]
165	484547328	[4;5;0;0;2;0]
165	149141600	[4;5;1;0;0;0]
165	399637637	[4;5;1;0;1;0]
165	160858304	[4;5;2;0;1;0]
165	180597632	[4;6;0;0;0;0]
165	759901315	[4;6;0;0;1;0]
165	4975694763	[5;2;0;0;0;0]
165	200109568	[5;3;0;0;0;0]
165	243848512	[5;3;0;0;1;0]
165	306550872	[5;3;1;0;0;0]
165	899620363.6	[5;4;0;0;0;0]
165	2362312900	[5;4;0;0;1;0]
165	865236436	[5;4;0;0;2;0]
165	988280780	[5;4;1;0;0;0]
165	2255122345	[5;4;1;0;1;0]
165	795447870.7	[5;4;2;0;0;0]
165	1412102510	[5;4;2;0;1;0]
165	894711466.7	[5;5;0;0;0;0]
165	926137078.5	[5;5;0;0;1;0]
165	138264200	[5;5;1;0;0;0]
165	189194608	[5;5;2;0;1;0]
165	122362528	[5;6;0;0;0;0]
165	933220960	[5;6;0;0;1;0]
165	123014592	[5;6;0;0;2;0]
165	284476960	[5;6;1;0;1;0]
165	505481056.5	[6;2;0;0;0;0]
165	360126592	[6;3;0;0;0;0]
165	391581568	[6;3;1;0;0;0]
165	165876288	[6;4;0;0;0;0]
165	83836024	[6;4;0;0;1;0]

165	210811040	[6;4;1;0;0;0]
165	374810944	[6;5;0;0;0;0]
165	1610413760	[6;5;0;0;1;0]
165	1236739971	[6;5;1;0;1;0]
165	780492100	[6;5;2;0;1;0]
165	133614400	[6;5;3;0;0;0]

Watanabe et al MSFragger-Glyco Search

Site	AUC	Glycan
234	693213150.4	[0;1;0;0;0;0]
234	1280674449	[3;3;0;0;0;0]
234	1067708160	[3;3;0;0;1;0]
234	1225854539	[3;4;0;0;0;0]
234	1648556898	[3;4;0;0;1;0]
234	473868292.8	[3;5;0;0;1;0]
234	255227360	[3;6;0;0;1;0]
234	1274717178	[4;3;0;0;0;0]
234	511998476	[4;3;0;0;1;0]
234	83941220	[4;4;0;0;0;0]
234	308470932.7	[4;4;0;0;1;0]
234	25830932	[4;4;1;0;1;0]
234	307244360	[4;5;0;0;1;0]
234	5891069560	[5;2;0;0;0;0]
234	2781591891	[5;3;0;0;0;0]
234	543988346	[5;3;0;0;1;0]
234	227842166	[5;4;0;0;1;0]
234	4154298546	[6;2;0;0;0;0]
234	938251395	[6;3;0;0;0;0]
234	12635134413	[7;2;0;0;0;0]
234	15660429639	[8;2;0;0;0;0]
234	19665432219	[9;2;0;0;0;0]

Watanabe et al MSFragger-Glyco Search

Site	AUC	Glycan
282	21574000	[0;1;0;0;1;0]
282	1009535623	[3;3;0;0;0;0]
282	1254121600	[3;4;0;0;0;0]
282	1912940318	[3;4;0;0;1;0]
282	3054273754	[3;5;0;0;0;0]
282	7800354050	[3;5;0;0;1;0]
282	367744464	[3;6;0;0;0;0]
282	351907270.7	[3;6;0;0;1;0]
282	61171014	[4;3;0;0;1;0]
282	355354741	[4;4;0;0;0;0]
282	700854864.4	[4;4;0;0;1;0]
282	211442448	[4;4;1;0;1;0]
282	314613856	[4;5;0;0;0;0]
282	843930503.3	[4;5;0;0;1;0]
282	468285262.5	[4;5;1;0;0;0]
282	887828290	[4;5;1;0;1;0]
282	1034561239	[4;6;0;0;1;0]
282	141866592	[5;2;0;0;0;0]
282	256689526	[5;3;0;0;0;0]
282	165709344	[5;4;0;0;0;0]
282	1262343019	[5;4;0;0;1;0]
282	187563680	[5;4;1;0;0;0]
282	653560580	[5;4;1;0;1;0]
282	53929472	[5;4;2;0;1;0]
282	426835640	[5;5;0;0;0;0]
282	3114817867	[5;5;0;0;1;0]
282	559537150	[5;5;1;0;0;0]
282	1204909033	[5;5;1;0;1;0]
282	141568464	[5;5;2;0;1;0]
282	161159184	[5;6;0;0;0;0]
282	1804115580	[5;6;0;0;1;0]
282	284918936	[5;6;1;0;1;0]
282	1211216445	[6;5;0;0;0;0]
282	4331421164	[6;5;0;0;1;0]
282	984988540	[6;5;1;0;0;0]
282	2999834620	[6;5;1;0;1;0]
282	242990176	[6;5;2;0;0;0]
282	141186352	[6;5;3;0;1;0]
282	77167316	[6;6;0;0;0;0]
282	131333256	[6;6;0;0;1;0]
282	244596920	[6;6;1;0;1;0]
282	108989024	[7;6;1;0;1;0]

Watanabe et al MSFragger-Glyco Search

Site	AUC	Glycan
331	80745526	[0;1;0;0;0;0]
331	213997824	[0;1;0;0;1;0]

331	59847160	[12;2;0;0;0;0]
331	46161008	[3;2;0;0;1;0]
331	290983210.7	[3;3;0;0;0;0]
331	1782269249	[3;3;0;0;1;0]
331	13532834570	[3;4;0;0;1;0]
331	14937376744	[3;5;0;0;1;0]
331	669633880.8	[3;5;0;0;2;0]
331	1042192437	[3;5;1;0;1;0]
331	2378963710	[3;6;0;0;1;0]
331	127559112	[4;3;0;0;0;0]
331	567750461.3	[4;3;0;0;1;0]
331	119187376	[4;3;1;0;1;0]
331	6472161772	[4;4;0;0;1;0]
331	8166257423	[4;4;1;0;1;0]
331	8197381000	[4;5;0;0;1;0]
331	1852020775	[4;5;1;0;1;0]
331	332474192	[4;5;2;0;1;0]
331	1316400510	[4;6;0;0;1;0]
331	1359453852	[5;2;0;0;0;0]
331	3413293479	[5;4;0;0;1;0]
331	1298942720	[5;4;0;0;2;0]
331	8505297055	[5;4;1;0;1;0]
331	768464260	[5;4;1;0;2;0]
331	1062101742	[5;4;1;0;3;0]
331	1791171070	[5;4;2;0;1;0]
331	2706771168	[5;5;0;0;1;0]
331	279667795.5	[5;5;0;0;3;0]
331	814660757.1	[5;5;1;0;1;0]
331	549471620	[5;5;2;0;1;0]
331	1505269760	[5;6;0;0;1;0]
331	166346832	[5;6;0;0;3;0]
331	808939200	[5;6;1;0;1;0]
331	621597310	[5;6;2;0;1;0]
331	77468896	[6;2;0;0;0;0]
331	1787711490	[6;5;0;0;1;0]
331	160695976	[6;5;0;0;3;0]
331	2693758780	[6;5;1;0;1;0]
331	1180461888	[6;5;2;0;1;0]
331	909682560	[6;5;3;0;1;0]
331	123541304	[7;2;0;0;0;0]
331	1953141500	[7;4;0;0;0;0]
331	172691056	[7;6;2;0;1;0]
331	614013440	[9;6;0;0;0;0]

Watanabe et al MSFragger-Glyco Search

Site	AUC	Glycan
343	1067361287	[3;3;0;0;1;0]
343	3711370037	[3;4;0;0;1;0]
343	4501770500	[3;5;0;0;1;0]
343	576404286	[4;3;0;0;1;0]
343	1289584955	[4;4;0;0;1;0]
343	292617584	[4;4;0;0;2;0]
343	409233608	[4;4;1;0;1;0]
343	5060993550	[4;5;0;0;1;0]
343	717813950	[4;7;0;0;0;0]
343	1864031740	[5;2;0;0;0;0]
343	201758310.4	[5;3;0;0;1;0]
343	3206518693	[5;4;0;0;1;0]
343	611702660	[5;4;0;0;2;0]
343	1495317635	[5;4;1;0;1;0]
343	541090300	[5;4;2;0;1;0]
343	1420672770	[5;5;0;0;1;0]
343	230178560	[6;5;0;0;1;0]

Watanabe et al MSFragger-Glyco Search

Site	AUC	Glycan
603	990853120	[0;1;0;0;0;0]
603	1629028100	[3;3;0;0;0;0]
603	2924893700	[3;3;0;0;1;0]
603	10200696350	[3;4;0;0;1;0]
603	1674281310	[3;5;0;0;1;0]
603	2384129340	[4;3;0;0;0;0]
603	2479254913	[4;3;0;0;1;0]
603	8868219604	[4;4;0;0;1;0]
603	1249515255	[4;5;0;0;1;0]
603	22677842154	[5;2;0;0;0;0]
603	6189342420	[5;3;0;0;0;0]
603	1783634740	[5;3;0;0;1;0]
603	8272907286	[5;4;0;0;1;0]
603	1011580486	[5;4;1;0;1;0]

603	4535749600	[5;5;0;0;1;0]
603	2082480649	[6;2;0;0;0;0]
603	2163377407	[6;3;0;0;0;0]
603	563456450	[6;3;0;0;1;0]
603	3058849660	[6;5;0;0;1;0]
603	2994268883	[7;2;0;0;0;0]
603	480081183	[8;2;0;0;0;0]

Watanabe et al MSFragger-Glyco Search

Site	AUC	Glycan
616	208693632	[0;1;0;0;0;0]
616	2323934312	[3;3;0;0;0;0]
616	857830182	[3;3;0;0;1;0]
616	1674256308	[3;4;0;0;0;0]
616	3310565165	[3;4;0;0;1;0]
616	1125112534	[3;5;0;0;0;0]
616	611665472	[3;5;0;0;1;0]
616	172493856	[3;6;0;0;0;0]
616	417909088	[3;6;0;0;1;0]
616	1594131619	[4;3;0;0;0;0]
616	957545724	[4;3;0;0;1;0]
616	868969981.5	[4;4;0;0;0;0]
616	2562042511	[4;4;0;0;1;0]
616	85009512	[4;4;1;0;0;0]
616	759138465	[4;4;1;0;1;0]
616	1071863040	[4;5;0;0;0;0]
616	678239026	[4;5;0;0;1;0]
616	202338592	[4;5;1;0;0;0]
616	496205760	[4;5;1;0;1;0]
616	2285253553	[5;2;0;0;0;0]
616	77218768	[5;2;1;0;1;0]
616	1007341439	[5;3;0;0;0;0]
616	395896472.8	[5;3;0;0;1;0]
616	233274904	[5;3;1;0;0;0]
616	1468932984	[5;4;0;0;0;0]
616	1948914164	[5;4;0;0;1;0]
616	294262670.7	[5;4;1;0;0;0]
616	996874350.3	[5;4;1;0;1;0]
616	277540608	[5;4;1;0;3;0]
616	375312368.5	[5;5;0;0;0;0]
616	592848812.4	[5;5;0;0;1;0]
616	540724545.3	[5;5;1;0;0;0]
616	952705150	[5;5;1;0;1;0]
616	156771008	[5;6;0;0;1;0]
616	479852288	[6;2;0;0;0;0]
616	707608830	[6;3;0;0;0;0]
616	35898400	[6;4;0;0;2;0]
616	365510272	[6;5;0;0;0;0]
616	1807590421	[6;5;0;0;1;0]
616	346632706.7	[6;5;1;0;0;0]
616	332965991.5	[6;5;1;0;1;0]
616	326216576	[6;5;2;0;1;0]
616	445016128	[6;6;1;0;1;0]
616	256189676	[7;6;1;0;1;0]

Watanabe et al MSFragger-Glyco Search

Site	AUC	Glycan
657	120407272	[0;1;0;0;0;0]
657	214946938	[0;1;0;0;1;0]
657	1271248429	[3;3;0;0;1;0]
657	6223887019	[3;4;0;0;1;0]
657	370926016	[3;4;1;0;0;0]
657	4163783007	[3;5;0;0;1;0]
657	1511243912	[3;6;0;0;1;0]
657	713376656.7	[3;7;0;0;1;0]
657	7923713	[4;4;0;0;0;0]
657	922870022.6	[4;4;0;0;1;0]
657	164605984	[4;4;1;0;0;0]
657	2130781255	[4;4;1;0;1;0]
657	2836061205	[4;5;0;0;1;0]
657	1972094176	[4;5;1;0;1;0]
657	1978763733	[4;6;0;0;1;0]
657	1149324030	[5;2;0;0;0;0]
657	2705524992	[5;4;0;0;1;0]
657	2284332905	[5;4;1;0;1;0]
657	529404491.3	[5;4;2;0;1;0]
657	2872458146	[5;5;0;0;1;0]
657	205436496	[5;5;0;0;3;0]
657	937840145	[5;5;1;0;1;0]
657	291771232	[5;5;2;0;1;0]

657	1527187070	[5;6;0;0;1;0]
657	281983584	[5;6;1;0;1;0]
657	692749904	[5;6;2;0;1;0]
657	1399595648	[6;5;0;0;1;0]
657	1631294336	[6;5;1;0;1;0]
657	913563398	[6;5;2;0;1;0]
657	126526032	[7;6;0;0;0;0]
657	279033728	[7;6;1;0;1;0]
657	233758976	[7;6;1;0;3;0]
657	1265148331	[7;6;2;0;1;0]

Watanabe et al MSFragger-Glyco Search

Site	AUC	Glycan
801	1975662080	[0;1;0;0;0;0]
801	668551702.5	[3;2;0;0;0;0]
801	206205664	[3;2;0;0;1;0]
801	1667890643	[3;3;0;0;0;0]
801	956101117	[3;3;0;0;1;0]
801	2190021153	[3;4;0;0;0;0]
801	4860848935	[3;4;0;0;1;0]
801	991188516.9	[3;5;0;0;0;0]
801	2757675792	[3;5;0;0;1;0]
801	513043776	[3;9;0;0;1;0]
801	2403988656	[4;2;0;0;0;0]
801	3229050364	[4;3;0;0;0;0]
801	631410193.6	[4;3;0;0;1;0]
801	906734636.7	[4;3;1;0;0;0]
801	109200264	[4;3;1;0;1;0]
801	1040299850	[4;4;0;0;0;0]
801	2254047025	[4;4;0;0;1;0]
801	191427025.5	[4;4;0;0;2;0]
801	1590138861	[4;4;1;0;0;0]
801	3702178434	[4;4;1;0;1;0]
801	131719180	[4;5;0;0;0;0]
801	921592509.5	[4;5;0;0;1;0]
801	336688992	[4;5;1;0;1;0]
801	38276678	[4;7;0;0;0;0]
801	182868968	[4;7;0;0;1;0]
801	12555428044	[5;2;0;0;0;0]
801	821345826.4	[5;2;1;0;1;0]
801	3053842655	[5;3;0;0;0;0]
801	350829611.1	[5;3;0;0;1;0]
801	4114324447	[5;3;1;0;0;0]
801	493732650.9	[5;4;0;0;0;0]
801	933057348.2	[5;4;0;0;1;0]
801	330587648	[5;4;0;0;2;0]
801	1117903677	[5;4;1;0;0;0]
801	1582789810	[5;4;1;0;1;0]
801	466307968	[5;4;2;0;1;0]
801	63427446.67	[5;5;0;0;0;0]
801	175972288	[5;5;0;0;1;0]
801	94814434	[5;5;1;0;1;0]
801	3128928658	[6;2;0;0;0;0]
801	2366056624	[6;3;0;0;0;0]
801	457142485.5	[6;3;0;0;1;0]
801	2368922946	[6;3;1;0;0;0]
801	67311328	[6;3;1;0;1;0]
801	130287461.3	[6;4;0;0;0;0]
801	5881273360	[7;2;0;0;0;0]
801	1847602111	[8;2;0;0;0;0]
801	23036992	[9;2;0;0;0;0]

Watanabe et al MSFragger-Glyco Search

Site	AUC	Glycan
1074	484358664	[0;1;0;0;0;0]
1074	24751450	[0;1;0;0;1;0]
1074	319112544	[0;2;0;0;0;0]
1074	777491710	[1;2;0;0;0;0]
1074	47906640	[1;2;0;0;1;0]
1074	578948990	[2;2;0;0;0;0]
1074	1054393405	[3;2;0;0;0;0]
1074	223633968	[3;2;0;0;1;0]
1074	3023971069	[3;3;0;0;0;0]
1074	3325962273	[3;3;0;0;1;0]
1074	1309152623	[3;4;0;0;0;0]
1074	9111989733	[3;4;0;0;1;0]
1074	490533358.8	[3;5;0;0;0;0]
1074	6994104999	[3;5;0;0;1;0]
1074	2825535518	[3;6;0;0;1;0]
1074	182785680	[3;7;0;0;1;0]

1074	2961509626	[4;2;0;0;0;0]
1074	75078108.5	[4;2;0;0;1;0]
1074	4212167721	[4;3;0;0;0;0]
1074	4683065071	[4;3;0;0;1;0]
1074	54762174.67	[4;3;0;0;2;0]
1074	361437601.6	[4;3;1;0;0;0]
1074	900292967.6	[4;3;1;0;1;0]
1074	60071984	[4;3;1;0;2;0]
1074	652492973.6	[4;4;0;0;0;0]
1074	4336960471	[4;4;0;0;1;0]
1074	162770888	[4;4;0;0;2;0]
1074	210759657.7	[4;4;1;0;0;0]
1074	2214414668	[4;4;1;0;1;0]
1074	263867421.6	[4;5;0;0;0;0]
1074	3185494384	[4;5;0;0;1;0]
1074	63556352	[4;5;0;0;2;0]
1074	1125680228	[4;5;1;0;1;0]
1074	4971872	[4;5;2;0;0;0]
1074	53131864	[4;6;0;0;0;0]
1074	541662044.3	[4;6;0;0;1;0]
1074	123937880	[4;7;0;0;1;0]
1074	12742504886	[5;2;0;0;0;0]
1074	1813984343	[5;2;1;0;1;0]
1074	3247361495	[5;3;0;0;0;0]
1074	3460591482	[5;3;0;0;1;0]
1074	1046289684	[5;3;1;0;0;0]
1074	797116797.3	[5;4;0;0;0;0]
1074	3602023722	[5;4;0;0;1;0]
1074	362023876.7	[5;4;0;0;2;0]
1074	229551094.2	[5;4;1;0;0;0]
1074	1793768307	[5;4;1;0;1;0]
1074	34276441	[5;4;1;0;2;0]
1074	172869736	[5;4;1;0;3;0]
1074	330248784	[5;4;2;0;1;0]
1074	143032040	[5;5;0;0;0;0]
1074	2580768636	[5;5;0;0;1;0]
1074	8722936	[5;5;0;0;2;0]
1074	214838429	[5;5;0;0;3;0]
1074	34314048	[5;5;1;0;0;0]
1074	1565838877	[5;5;1;0;1;0]
1074	18489224	[5;5;1;0;2;0]
1074	129593275.2	[5;5;2;0;1;0]
1074	7845811.5	[5;6;0;0;0;0]
1074	804002625.2	[5;6;0;0;1;0]
1074	10114280	[5;6;0;0;2;0]
1074	321946734.9	[5;6;1;0;1;0]
1074	123826718	[5;6;2;0;1;0]
1074	1691818056	[6;2;0;0;0;0]
1074	3105513445	[6;3;0;0;0;0]
1074	401435265.2	[6;3;0;0;1;0]
1074	1453755682	[6;3;1;0;0;0]
1074	242486650.5	[6;3;1;0;1;0]
1074	75307208	[6;4;0;0;0;0]
1074	68632200	[6;4;0;0;1;0]
1074	156043987.9	[6;5;0;0;0;0]
1074	2175412051	[6;5;0;0;1;0]
1074	58555872	[6;5;0;0;2;0]
1074	1130461124	[6;5;0;0;3;0]
1074	30815181	[6;5;1;0;0;0]
1074	893794821.6	[6;5;1;0;1;0]
1074	210293552	[6;5;2;0;1;0]
1074	59686268	[6;5;2;0;3;0]
1074	47492388	[6;5;3;0;1;0]
1074	441714105.9	[6;6;0;0;1;0]
1074	41723800	[6;6;0;0;2;0]
1074	235197640.2	[6;6;1;0;1;0]
1074	130459280	[6;7;0;0;1;0]
1074	967345272.9	[7;2;0;0;0;0]
1074	62794504	[7;6;0;0;2;0]
1074	1041635200	[7;6;0;0;3;0]
1074	439764158.5	[7;6;1;0;1;0]
1074	374531584	[7;6;2;0;1;0]
1074	84730168	[7;7;0;0;1;0]
1074	529065905.3	[8;2;0;0;0;0]
1074	100960688	[8;8;0;0;0;0]
1074	114621392	[9;2;0;0;0;0]
1074	92613936	[9;8;0;0;0;0]

Watanabe et al MSFragger-Glyco Search

Site	AUC	Glycan
1098	234940104	[0;1;0;0;0;0]
1098	6778452614	[3;3;0;0;0;0]
1098	4171117713	[3;3;0;0;1;0]
1098	7032046586	[3;4;0;0;0;0]
1098	9061630276	[3;4;0;0;1;0]
1098	4088524940	[3;5;0;0;0;0]
1098	3834871964	[3;5;0;0;1;0]
1098	2301478773	[3;6;0;0;0;0]
1098	1514938101	[3;6;0;0;1;0]
1098	30889706	[3;7;0;0;0;0]
1098	68001600	[4;2;0;0;0;0]
1098	13640686	[4;2;0;0;1;0]
1098	9670244103	[4;3;0;0;0;0]
1098	6853203008	[4;3;0;0;1;0]
1098	1199612194	[4;3;1;0;0;0]
1098	1257165658	[4;3;1;0;1;0]
1098	4640975752	[4;4;0;0;0;0]
1098	1781657032	[4;4;0;0;1;0]
1098	710496380	[4;4;0;0;2;0]
1098	6168232280	[4;4;1;0;0;0]
1098	5315260118	[4;4;1;0;1;0]
1098	2031139897	[4;5;0;0;0;0]
1098	1203674355	[4;5;0;0;1;0]
1098	400276224	[4;5;0;0;2;0]
1098	1588012353	[4;5;1;0;0;0]
1098	2174735300	[4;5;1;0;1;0]
1098	820434200.3	[4;6;0;0;0;0]
1098	570766822.4	[4;6;0;0;1;0]
1098	185897808	[4;6;0;0;2;0]
1098	1655005684	[4;6;1;0;0;0]
1098	8505938200	[5;2;0;0;0;0]
1098	2735715825	[5;2;1;0;1;0]
1098	15252292526	[5;3;0;0;0;0]
1098	3773544041	[5;3;0;0;1;0]
1098	3129246829	[5;3;1;0;0;0]
1098	1016730387	[5;4;0;0;0;0]
1098	527627943	[5;4;0;0;1;0]
1098	3910667255	[5;4;1;0;0;0]
1098	4000384648	[5;4;1;0;1;0]
1098	177447217.7	[5;4;2;0;0;0]
1098	215557732	[5;4;2;0;1;0]
1098	605757703.5	[5;5;0;0;0;0]
1098	246323638	[5;5;0;0;1;0]
1098	323884992	[5;5;0;0;2;0]
1098	4352748000	[5;5;0;0;3;0]
1098	1827535119	[5;5;1;0;0;0]
1098	2176222954	[5;5;1;0;1;0]
1098	505822383.4	[5;5;2;0;0;0]
1098	428270176	[5;5;2;0;1;0]
1098	283499206	[5;6;0;0;0;0]
1098	354902592	[5;6;0;0;1;0]
1098	817013760	[5;6;1;0;1;0]
1098	74038944	[5;6;2;0;1;0]
1098	2418200267	[6;2;0;0;0;0]
1098	7613198991	[6;3;0;0;0;0]
1098	1145406491	[6;3;0;0;1;0]
1098	2630462485	[6;3;1;0;0;0]
1098	726470741.2	[6;3;1;0;1;0]
1098	330919964	[6;4;0;0;0;0]
1098	119328352	[6;4;0;0;1;0]
1098	152682720	[6;4;1;0;0;0]
1098	124340482.7	[6;4;1;0;1;0]
1098	560989633.3	[6;5;0;0;0;0]
1098	184925303.4	[6;5;0;0;1;0]
1098	2166494890	[6;5;1;0;0;0]
1098	1212008767	[6;5;1;0;1;0]
1098	548809660	[6;5;1;0;3;0]
1098	285830944.8	[6;5;2;0;0;0]
1098	428289024	[6;5;2;0;1;0]
1098	352998881	[6;6;0;0;0;0]
1098	42130746.67	[6;6;0;0;1;0]
1098	3251345920	[6;6;0;0;2;0]
1098	1235072040	[6;6;1;0;0;0]
1098	1407055107	[6;6;1;0;1;0]
1098	105973836	[6;6;2;0;0;0]
1098	1619242125	[7;2;0;0;0;0]
1098	152145568	[7;4;0;0;0;0]

1098	22147260	[7;4;1;0;0;0]
1098	58307933.33	[7;6;0;0;0;0]
1098	1119097233	[7;6;1;0;0;0]
1098	352797639.4	[7;6;1;0;1;0]
1098	662826922.7	[7;6;2;0;0;0]
1098	124268725.3	[7;6;2;0;1;0]
1098	134760816	[7;6;3;0;0;0]
1098	413249568	[8;2;0;0;0;0]
1098	104656080	[9;2;0;0;0;0]

Watanabe et al MSFragger-Glyco Search

Site	AUC	Glycan
1134	57890128	[0;1;0;0;1;0]
1134	620355710	[3;3;0;0;1;0]
1134	1808421791	[3;4;0;0;1;0]
1134	601103791	[3;5;0;0;1;0]
1134	2349633950	[3;5;0;0;2;0]
1134	430194559.3	[3;6;0;0;1;0]
1134	1641130497	[3;6;0;0;2;0]
1134	155044288	[4;3;0;0;0;0]
1134	750268420	[4;3;0;0;1;0]
1134	617059774.7	[4;4;0;0;1;0]
1134	938785410	[4;4;1;0;1;0]
1134	742039040	[4;5;0;0;1;0]
1134	288005632	[5;2;0;0;0;0]
1134	534473472	[5;4;0;0;1;0]
1134	330052730.7	[5;5;0;0;1;0]
1134	465851712	[5;5;1;0;1;0]
1134	363100160	[5;5;2;0;1;0]
1134	735356740	[5;6;0;0;2;0]
1134	191440544	[5;6;2;0;1;0]

Watanabe et al MSFragger-Glyco Search

Site	AUC	Glycan
1173	236620864	[4;9;0;0;1;0]

Watanabe et al MSFragger-Glyco Search

Site	AUC	Glycan
1194	193247056	[3;5;0;0;1;0]
1194	529456841	[3;6;0;0;1;0]
1194	87399216	[3;7;0;0;1;0]
1194	464220544	[4;6;0;0;1;0]
1194	42914824	[4;7;0;0;1;0]
1194	369294864	[5;2;0;0;0;0]
1194	84096200	[5;6;1;0;1;0]
1194	505239488	[5;6;2;0;1;0]
1194	177318880	[7;6;2;0;1;0]

Figure S5. Zhang et al Searches by Site

Glycopeptide Format: SITE#[Hex;HexNAc;NeuAc;Fuc;Phosphate/Sulfate*]

Phosphate/Sulfate is an indicator variable instead of a quantifier: 0 indicates neither, 1 indicates a phosphorylated glycan, 2 indicates a sulfated glycan.
These are the log abundance values of the quantitations provided by each software. They are presented here for reference purposes only.

Figure S5a. Zhang et al search using Byonic

Site	AUC	Glycan
17	23100000	[4;5;2;0;1;0]
17	30900000	[4;6;0;0;1;0]
17	80100000	[5;5;1;0;1;0]
17	9.18E+08	[5;6;2;0;1;0]
17	1.62E+09	[5;6;3;0;1;0]
17	2.62E+08	[6;7;4;0;1;0]

Zhang et al Byonic Search

Site	AUC	Glycan
122	1.57E+09	[0;0;0;0;0;0]
122	19500000	[0;1;0;0;0;0]
122	2.10E+07	[0;1;0;0;1;0]
122	10100000	[0;2;0;0;0;0]
122	9160000	[0;2;0;0;1;0]
122	53200000	[1;2;0;0;0;0]
122	37600000	[10;2;0;0;0;0]
122	3010000	[10;6;1;0;1;0]
122	1.37E+08	[10;9;0;0;0;0]
122	1.59E+08	[10;9;0;0;1;0]
122	40900000	[2;2;0;0;0;0]
122	2.05E+08	[3;2;0;0;0;0]
122	2.31E+08	[3;3;0;0;0;0]
122	2.65E+08	[3;3;0;0;1;0]
122	88200000	[3;4;0;0;0;0]
122	2.88E+09	[3;4;0;0;1;0]
122	99600000	[3;4;1;0;0;0]
122	1.11E+08	[3;5;0;0;0;0]
122	4.86E+09	[3;5;0;0;1;0]
122	5.42E+08	[3;5;1;0;1;0]
122	9.04E+08	[3;6;0;0;0;0]
122	1.22E+08	[3;6;0;0;1;0]
122	1.60E+08	[3;6;1;0;1;0]
122	1.59E+08	[3;7;0;0;0;0]
122	2.13E+08	[3;7;0;0;1;0]
122	5.29E+08	[3;8;0;0;1;0]
122	2.66E+08	[3;9;0;0;1;0]
122	7.31E+08	[4;2;0;0;0;0]
122	1.04E+08	[4;2;0;0;1;0]
122	64100000	[4;3;0;0;0;0]
122	6.13E+08	[4;3;0;0;1;0]
122	1.43E+08	[4;3;1;0;0;0]
122	1.74E+08	[4;3;1;0;1;0]
122	1.30E+09	[4;4;0;0;0;0]
122	2.53E+08	[4;4;0;0;1;0]
122	69100000	[4;4;1;0;0;0]
122	2.28E+09	[4;4;1;0;1;0]
122	2.22E+08	[4;5;0;0;0;0]
122	5.17E+08	[4;5;0;0;1;0]
122	6.26E+09	[4;5;1;0;0;0]
122	3.82E+09	[4;5;1;0;1;0]
122	1.27E+08	[4;5;2;0;0;0]
122	7.88E+08	[4;5;2;0;1;0]
122	1.34E+09	[4;6;0;0;0;0]
122	2.56E+08	[4;6;0;0;1;0]
122	8.95E+08	[4;6;1;0;0;0]
122	29200000	[4;7;0;0;0;0]
122	1.74E+08	[4;7;0;0;1;0]
122	1.33E+08	[4;8;0;0;0;0]
122	1.38E+08	[4;9;0;0;1;0]
122	8.68E+09	[5;2;0;0;0;0]

122	2.02E+08	[5;3;0;0;0;0]
122	1.70E+09	[5;3;0;0;1;0]
122	1.64E+09	[5;3;1;0;0;0]
122	3.45E+09	[5;4;0;0;0;0]
122	1.07E+10	[5;4;0;0;1;0]
122	6.60E+09	[5;4;1;0;0;0]
122	3.78E+10	[5;4;1;0;1;0]
122	1.53E+09	[5;4;2;0;0;0]
122	5.96E+08	[5;4;2;0;1;0]
122	3.59E+09	[5;5;0;0;0;0]
122	2.15E+09	[5;5;0;0;1;0]
122	3.53E+08	[5;5;1;0;0;0]
122	2.01E+09	[5;5;1;0;1;0]
122	21700000	[5;5;2;0;0;0]
122	2.57E+08	[5;5;2;0;1;0]
122	4.75E+09	[5;6;0;0;0;0]
122	66600000	[5;6;0;0;1;0]
122	1.31E+09	[5;6;1;0;1;0]
122	2.82E+08	[5;6;2;0;1;0]
122	32500000	[5;8;0;0;1;0]
122	3.55E+08	[6;2;0;0;0;0]
122	3.08E+09	[6;3;0;0;0;0]
122	1.76E+08	[6;3;0;0;1;0]
122	2.70E+09	[6;3;1;0;0;0]
122	8.25E+08	[6;3;1;0;1;0]
122	2.43E+09	[6;4;0;0;0;0]
122	1.70E+09	[6;4;0;0;1;0]
122	5.66E+09	[6;4;1;0;0;0]
122	1.08E+09	[6;4;1;0;1;0]
122	4.61E+08	[6;5;0;0;0;0]
122	3.07E+09	[6;5;0;0;1;0]
122	3.47E+09	[6;5;1;0;0;0]
122	1.19E+09	[6;5;1;0;1;0]
122	1.13E+09	[6;5;2;0;0;0]
122	2.98E+08	[6;5;2;0;1;0]
122	2.75E+08	[6;5;3;0;0;0]
122	3.21E+08	[6;5;3;0;1;0]
122	1.06E+09	[6;6;0;0;0;0]
122	5.08E+08	[6;6;0;0;1;0]

122	1.65E+09	[6;6;1;0;0;0]
122	1.22E+09	[6;6;1;0;1;0]
122	32700000	[6;6;2;0;0;0]
122	89800000	[6;6;2;0;1;0]
122	87900000	[6;6;3;0;1;0]
122	77300000	[6;7;0;0;0;0]
122	5.00E+07	[6;7;0;0;1;0]
122	4170000	[6;9;0;0;0;0]
122	3.52E+09	[7;2;0;0;0;0]
122	2.14E+08	[7;4;0;0;0;0]
122	63400000	[7;4;0;0;1;0]
122	86100000	[7;4;1;0;0;0]
122	1.34E+08	[7;5;0;0;0;0]
122	5.19E+08	[7;5;0;0;1;0]
122	6.47E+08	[7;5;1;0;1;0]
122	2.10E+08	[7;5;2;0;1;0]
122	1.32E+09	[7;6;0;0;0;0]
122	93800000	[7;6;0;0;1;0]
122	1.00E+09	[7;6;1;0;0;0]
122	1.26E+09	[7;6;1;0;1;0]
122	4.56E+08	[7;6;2;0;0;0]
122	1.04E+08	[7;6;2;0;1;0]
122	1.45E+08	[7;6;3;0;0;0]
122	8.40E+07	[7;6;3;0;1;0]
122	28400000	[7;6;4;0;0;0]
122	98500000	[7;6;4;0;1;0]
122	2.51E+08	[7;7;0;0;0;0]
122	7.11E+08	[7;7;0;0;1;0]
122	32600000	[7;7;2;0;1;0]
122	1.34E+08	[7;7;3;0;1;0]
122	98400000	[7;8;0;0;0;0]
122	2.94E+09	[8;2;0;0;0;0]
122	1.45E+08	[8;5;0;0;1;0]
122	4.01E+08	[8;6;1;0;0;0]
122	1.32E+08	[8;6;1;0;1;0]
122	4.25E+08	[8;7;0;0;0;0]
122	1.62E+08	[8;7;0;0;1;0]
122	2.25E+08	[8;7;1;0;0;0]
122	1.40E+08	[8;7;1;0;1;0]

122	1.58E+08	[8;7;2;0;1;0]
122	1.08E+08	[8;7;3;0;1;0]
122	25500000	[8;8;0;0;0;0]
122	53700000	[8;8;0;0;1;0]
122	3.56E+08	[9;2;0;0;0;0]
122	415000	[9;6;0;0;0;0]
122	73400000	[9;6;2;0;1;0]
122	49900000	[9;8;0;0;0;0]
122	65300000	[9;9;0;0;1;0]

Zhang et al Byonic Search

Site	AUC	Glycan
149	4.24E+08	[11;11;1;0;0;0]
149	8.20E+07	[3;6;1;0;1;0]
149	43900000	[4;4;0;0;1;0]
149	92100000	[4;4;1;0;0;0]
149	35100000	[4;5;0;0;1;0]
149	75200000	[4;5;1;0;0;0]
149	1.62E+08	[4;5;1;0;1;0]
149	92200000	[4;5;2;0;0;0]
149	17600000	[5;3;0;0;1;0]
149	1.52E+08	[5;4;0;0;1;0]
149	1.39E+08	[5;4;1;0;0;0]
149	3.47E+08	[5;4;1;0;1;0]
149	1.68E+08	[5;4;2;0;1;0]
149	2.20E+08	[5;5;0;0;1;0]
149	35800000	[5;5;1;0;0;0]
149	59600000	[5;5;1;0;1;0]
149	40900000	[5;6;2;0;1;0]
149	9310000	[6;3;0;0;0;0]
149	82100000	[6;5;1;0;1;0]
149	1.30E+08	[6;5;2;0;1;0]
149	86400000	[6;5;3;0;1;0]
149	47500000	[6;6;0;0;1;0]
149	6890000	[6;6;1;0;1;0]
149	2.80E+07	[6;6;2;0;0;0]
149	5.10E+07	[6;6;2;0;1;0]
149	9460000	[7;6;1;0;0;0]
149	1.39E+08	[7;6;2;0;1;0]
149	1.12E+08	[7;6;3;0;1;0]
149	50600000	[7;7;2;0;1;0]
149	6130000	[7;7;3;0;1;0]

Zhang et al Byonic Search

Site	AUC	Glycan
165	82100000	[0;0;0;0;0;0]
165	58500000	[10;9;0;0;0;0]
165	47100000	[3;2;0;0;0;0]
165	26900000	[3;5;0;0;1;0]
165	15300000	[3;6;1;0;1;0]
165	17800000	[3;6;2;0;1;0]
165	1.18E+08	[4;4;1;0;1;0]
165	1.36E+08	[4;5;0;0;0;0]
165	5.48E+08	[4;5;0;0;1;0]
165	68500000	[4;5;1;0;0;0]
165	8.40E+08	[4;5;2;0;0;0]
165	15300000	[4;5;2;0;1;0]
165	6.10E+07	[5;2;0;0;0;0]
165	57100000	[5;4;0;0;1;0]
165	1.97E+08	[5;4;1;0;0;0]
165	1.40E+08	[5;4;1;0;1;0]
165	5.16E+08	[5;4;2;0;0;0]
165	9.99E+08	[5;4;2;0;1;0]
165	24900000	[5;5;0;0;0;0]
165	68900000	[5;5;0;0;1;0]
165	8.34E+08	[5;5;1;0;0;0]
165	5.13E+08	[5;5;1;0;1;0]
165	1.79E+08	[5;5;2;0;0;0]
165	1.20E+07	[5;5;2;0;1;0]
165	1.18E+08	[5;6;0;0;1;0]
165	71600000	[5;6;1;0;1;0]
165	2.28E+08	[5;6;2;0;1;0]
165	92600000	[5;6;3;0;1;0]
165	28100000	[6;3;0;0;0;0]
165	14700000	[6;3;1;0;0;0]
165	7100000	[6;3;1;0;1;0]
165	33500000	[6;4;1;0;1;0]
165	1.21E+08	[6;5;0;0;1;0]
165	2.56E+08	[6;5;1;0;0;0]
165	1.63E+08	[6;5;1;0;1;0]

165	3.78E+08	[6;5;2;0;0;0]
165	1.34E+09	[6;5;2;0;1;0]
165	4.07E+08	[6;5;3;0;0;0]
165	8.34E+08	[6;5;3;0;1;0]
165	2.01E+08	[6;6;0;0;1;0]
165	47900000	[6;6;1;0;0;0]
165	3.02E+08	[6;6;1;0;1;0]
165	5.53E+08	[6;6;2;0;0;0]
165	5.66E+08	[6;6;2;0;1;0]
165	75900000	[6;6;3;0;1;0]
165	32200000	[7;2;0;0;0;0]
165	28400000	[7;4;0;0;1;0]
165	60500000	[7;5;2;0;1;0]
165	2.61E+08	[7;6;0;0;1;0]
165	6.20E+08	[7;6;1;0;0;0]
165	7.49E+08	[7;6;1;0;1;0]
165	33200000	[7;6;2;0;0;0]
165	1.22E+09	[7;6;2;0;1;0]
165	68700000	[7;6;3;0;0;0]
165	1.04E+09	[7;6;3;0;1;0]
165	1.11E+08	[7;6;4;0;0;0]
165	4.63E+08	[7;6;4;0;1;0]
165	1.32E+08	[7;7;0;0;0;0]
165	61100000	[7;7;0;0;1;0]
165	5.12E+08	[7;7;2;0;1;0]
165	1.30E+07	[7;7;3;0;1;0]
165	32800000	[8;6;1;0;0;0]
165	2.62E+08	[8;6;1;0;1;0]
165	85100000	[8;7;1;0;0;0]
165	72400000	[8;7;1;0;1;0]
165	1.03E+08	[8;7;2;0;1;0]
165	45900000	[9;6;2;0;1;0]

Zhang et al Byonic Search

Site	AUC	Glycan
234	1.73E+08	[3;8;0;0;0;0]
234	42800000	[3;8;0;0;1;0]
234	1.23E+08	[3;9;0;0;0;0]
234	3.30E+09	[3;9;0;0;1;0]
234	4.02E+08	[4;8;0;0;0;0]
234	1.20E+08	[5;5;1;0;0;0]
234	3560000	[5;6;0;0;1;0]
234	6.90E+07	[6;6;2;0;0;0]
234	78500000	[7;7;0;0;1;0]

Zhang et al Byonic Search

Site	AUC	Glycan
282	2.20E+07	[11;2;0;0;0;0]
282	3020000	[3;6;0;0;0;0]
282	52600000	[3;6;1;0;1;0]
282	1.28E+08	[3;6;2;0;1;0]
282	3860000	[3;7;0;0;1;0]
282	2.30E+07	[3;8;0;0;1;0]
282	85100000	[3;9;0;0;0;0]
282	33900000	[3;9;0;0;1;0]
282	1310000	[4;2;0;0;0;0]
282	3.06E+08	[4;4;1;0;0;0]
282	60400000	[4;4;1;0;1;0]
282	1.14E+08	[4;5;1;0;0;0]
282	4.28E+08	[4;5;1;0;1;0]
282	9.23E+08	[4;5;2;0;0;0]
282	3.20E+08	[4;5;2;0;1;0]
282	39400000	[4;6;1;0;0;0]
282	68100000	[4;7;0;0;1;0]
282	3.30E+07	[5;4;0;0;1;0]
282	63100000	[5;4;1;0;0;0]
282	3.04E+08	[5;4;1;0;1;0]
282	5.80E+07	[5;4;2;0;0;0]
282	82100000	[5;4;2;0;1;0]
282	85800000	[5;5;0;0;1;0]
282	3.34E+08	[5;5;1;0;0;0]
282	1.68E+09	[5;5;1;0;1;0]
282	1.60E+08	[5;5;2;0;0;0]
282	9680000	[5;5;2;0;1;0]
282	4.34E+08	[5;6;0;0;1;0]
282	4.32E+08	[5;6;1;0;1;0]
282	41800000	[5;6;2;0;1;0]
282	10500000	[5;8;0;0;1;0]
282	42900000	[6;3;1;0;0;0]
282	27900000	[6;3;1;0;1;0]

282	1.45E+08	[6;4;1;0;0;0]
282	43100000	[6;4;1;0;1;0]
282	90600000	[6;5;0;0;1;0]
282	35800000	[6;5;1;0;0;0]
282	1.33E+09	[6;5;1;0;1;0]
282	3.37E+08	[6;5;2;0;0;0]
282	4.57E+08	[6;5;2;0;1;0]
282	1.15E+08	[6;5;3;0;0;0]
282	2.33E+08	[6;5;3;0;1;0]
282	5.20E+08	[6;6;0;0;1;0]
282	6.62E+08	[6;6;1;0;0;0]
282	6.09E+08	[6;6;1;0;1;0]
282	74400000	[6;6;2;0;0;0]
282	1.53E+08	[6;6;2;0;1;0]
282	1.53E+08	[6;7;0;0;1;0]
282	8650000	[7;4;1;0;0;0]
282	30800000	[7;5;0;0;0;0]
282	78600000	[7;5;0;0;1;0]
282	2.59E+08	[7;5;1;0;1;0]
282	25500000	[7;5;2;0;1;0]
282	5.46E+08	[7;6;0;0;0;0]
282	3.84E+08	[7;6;0;0;1;0]
282	2.01E+08	[7;6;1;0;0;0]
282	3.64E+08	[7;6;1;0;1;0]
282	1.64E+08	[7;6;2;0;0;0]
282	9.40E+08	[7;6;2;0;1;0]
282	1.27E+08	[7;6;3;0;0;0]
282	9.45E+08	[7;6;3;0;1;0]
282	2.49E+08	[7;6;4;0;0;0]
282	83800000	[7;7;0;0;0;0]
282	18200000	[7;7;0;0;1;0]
282	4.26E+08	[7;7;2;0;1;0]
282	2.60E+08	[7;7;3;0;1;0]
282	2.86E+08	[8;6;1;0;0;0]
282	1.28E+08	[8;6;1;0;1;0]
282	2.06E+08	[8;7;0;0;0;0]
282	1.62E+08	[8;7;0;0;1;0]
282	13500000	[8;7;1;0;0;0]
282	3.31E+08	[8;7;1;0;1;0]
282	49700000	[8;7;2;0;1;0]
282	2.93E+08	[9;6;2;0;1;0]

Zhang et al Byonic Search

Site	AUC	Glycan
331,343	49900000	[10;2;0;0;0;0]
331	27800000	[3;5;0;0;1;0]
331	5.78E+08	[3;6;1;0;1;0]
331	65400000	[4;3;0;0;1;0]
331	40800000	[4;4;1;0;1;0]
331	1.39E+08	[4;5;0;0;1;0]
331	4.51E+08	[4;5;1;0;0;0]
331	97700000	[4;5;1;0;1;0]
331	3.82E+08	[4;5;2;0;0;0]
331	75200000	[4;6;0;0;0;0]
331	57900000	[4;6;1;0;0;0]
331	33700000	[5;3;1;0;0;0]
331	1.62E+08	[5;4;0;0;1;0]
331	1.60E+07	[5;4;1;0;0;0]
331	2.59E+09	[5;4;1;0;1;0]
331	1.45E+08	[5;4;2;0;0;0]
331	41300000	[5;4;2;0;1;0]
331	4.89E+08	[5;5;1;0;0;0]
331	3.87E+08	[5;5;1;0;1;0]
331	3.67E+08	[5;5;2;0;0;0]
331	1.69E+08	[5;6;0;0;0;0]
331	29200000	[5;6;1;0;1;0]
331	1.52E+08	[5;6;2;0;1;0]
331	40400000	[5;6;3;0;1;0]
331	1.57E+08	[6;3;0;0;0;0]
331	60600000	[6;3;1;0;1;0]
331	11800000	[6;4;1;0;0;0]
331	97300000	[6;5;1;0;0;0]
331	29500000	[6;5;1;0;1;0]
331	2.59E+08	[6;5;2;0;0;0]
331	3.40E+08	[6;5;2;0;1;0]
331	14900000	[6;5;3;0;0;0]
331	23200000	[6;5;3;0;1;0]
331	3.18E+08	[6;6;0;0;1;0]
331	1.18E+09	[6;6;1;0;0;0]

331	2.32E+09	[6;6;1;0;1;0]
331	1.94E+08	[6;6;2;0;0;0]
331	37300000	[6;6;2;0;1;0]
331	4.39E+08	[6;7;0;0;0;0]
331	2.78E+08	[6;7;0;0;1;0]
331	3330000	[7;2;0;0;0;0]
331	15500000	[7;5;2;0;1;0]
331	3.83E+08	[7;6;0;0;1;0]
331	2.48E+08	[7;6;1;0;1;0]
331	1.08E+08	[7;6;2;0;0;0]
331	2.90E+07	[7;6;2;0;1;0]
331	1.83E+08	[7;7;0;0;1;0]
331	59100000	[7;8;0;0;0;0]
331	1.07E+08	[8;6;1;0;1;0]
331	35200000	[8;7;0;0;0;0]
331,343	1.20E+08	[9;6;2;0;1;0]
331	25400000	[9;8;0;0;0;0]

Zhang et al Byonic Search

Site	AUC	Glycan
331,343	49900000	[10;2;0;0;0;0]
343	47200000	[10;2;0;0;0;0]
343	3.28E+08	[3;3;0;0;0;0]
343	1.35E+09	[3;3;0;0;1;0]
343	1.24E+09	[3;4;0;0;0;0]
343	8.42E+09	[3;4;0;0;1;0]
343	2.47E+09	[3;5;0;0;0;0]
343	7.38E+08	[3;5;0;0;1;0]
343	1.58E+08	[3;6;0;0;0;0]
343	1.51E+08	[3;6;0;0;1;0]
343	1.04E+08	[3;6;1;0;1;0]
343	47900000	[3;6;2;0;1;0]
343	8.06E+08	[3;7;0;0;1;0]
343	3.10E+08	[3;9;0;0;1;0]
343	5.90E+07	[4;2;0;0;1;0]
343	4.53E+08	[4;3;0;0;0;0]
343	2.38E+09	[4;3;0;0;1;0]
343	8.72E+08	[4;3;1;0;1;0]
343	1.10E+09	[4;4;0;0;0;0]
343	1.70E+09	[4;4;0;0;1;0]
343	1.61E+08	[4;4;1;0;0;0]
343	1.42E+09	[4;4;1;0;1;0]
343	3.75E+08	[4;5;0;0;0;0]
343	3.78E+09	[4;5;0;0;1;0]
343	3.56E+09	[4;5;1;0;0;0]
343	2.60E+09	[4;5;1;0;1;0]
343	2.88E+08	[4;5;2;0;1;0]
343	3.71E+08	[4;6;0;0;0;0]
343	2.83E+09	[4;6;0;0;1;0]
343	3.49E+08	[4;6;1;0;0;0]
343	25900000	[4;7;0;0;0;0]
343	1.04E+08	[4;7;0;0;1;0]
343	4.80E+07	[4;8;0;0;0;0]
343	3.57E+09	[5;2;0;0;0;0]
343	1.26E+09	[5;3;0;0;0;0]
343	2.02E+09	[5;3;0;0;1;0]
343	3.78E+08	[5;3;1;0;0;0]
343	2.64E+09	[5;4;0;0;0;0]
343	1.74E+10	[5;4;0;0;1;0]
343	5.87E+09	[5;4;1;0;0;0]
343	6.83E+09	[5;4;1;0;1;0]
343	45900000	[5;4;2;0;0;0]
343	3.35E+09	[5;4;2;0;1;0]
343	3.87E+08	[5;5;0;0;0;0]
343	1.74E+09	[5;5;0;0;1;0]
343	1.13E+08	[5;5;1;0;0;0]
343	1.60E+09	[5;5;1;0;1;0]
343	1.21E+09	[5;5;2;0;0;0]
343	3.42E+08	[5;5;2;0;1;0]
343	1.23E+09	[5;6;0;0;0;0]
343	1.14E+09	[5;6;0;0;1;0]
343	9.58E+08	[5;6;1;0;1;0]
343	2.94E+08	[5;6;2;0;1;0]
343	1040000	[5;6;3;0;1;0]
343	1.42E+09	[6;2;0;0;0;0]
343	2.40E+08	[6;3;0;0;0;0]
343	1.34E+09	[6;3;0;0;1;0]
343	2.83E+08	[6;3;1;0;0;0]
343	2.00E+08	[6;3;1;0;1;0]

343	48600000	[6;4;0;0;0;0]
343	5.43E+08	[6;4;0;0;1;0]
343	1.69E+08	[6;4;1;0;1;0]
343	26600000	[6;5;0;0;0;0]
343	1.96E+08	[6;5;0;0;1;0]
343	4.05E+08	[6;5;1;0;0;0]
343	1.39E+09	[6;5;1;0;1;0]
343	1.23E+08	[6;5;2;0;0;0]
343	1.57E+08	[6;5;2;0;1;0]
343	40900000	[6;5;3;0;0;0]
343	6.15E+08	[6;5;3;0;1;0]
343	3.14E+08	[6;6;0;0;0;0]
343	1.27E+09	[6;6;0;0;1;0]
343	2.08E+09	[6;6;1;0;0;0]
343	6.20E+08	[6;6;1;0;1;0]
343	3.01E+08	[6;6;2;0;0;0]
343	3.59E+08	[6;6;2;0;1;0]
343	38500000	[6;6;3;0;1;0]
343	1.51E+08	[6;7;0;0;0;0]
343	47200000	[6;7;0;0;1;0]
343	2.80E+09	[7;2;0;0;0;0]
343	2.05E+08	[7;4;1;0;0;0]
343	1.61E+08	[7;5;1;0;1;0]
343	1.00E+08	[7;5;2;0;1;0]
343	6.17E+08	[7;6;2;0;1;0]
343	4.60E+08	[7;6;3;0;1;0]
343	72700000	[7;6;4;0;0;0]
343	1.19E+08	[7;6;4;0;1;0]
343	1.56E+08	[7;7;0;0;1;0]
343	1.63E+08	[7;7;2;0;1;0]
343	54900000	[7;7;3;0;1;0]
343	3.03E+09	[8;2;0;0;0;0]
343	27100000	[8;6;1;0;0;0]
343	23700000	[8;7;2;0;1;0]
343	2.25E+08	[9;2;0;0;0;0]
331,343	1.20E+08	[9;6;2;0;1;0]

Zhang et al Byonic Search

Site	AUC	Glycan
603,616	7.10E+07	[10;6;1;0;1;0]
603,616	3500000	[3;3;0;0;1;0]
603,616	50100000	[5;4;2;0;1;0]
603,616	63300000	[5;5;2;0;1;0]
603,616	87700000	[5;6;2;0;1;0]
603,616	88400000	[5;6;3;0;1;0]
603,616	33400000	[6;5;1;0;0;0]
603,616	40400000	[6;5;2;0;0;0]
603,616	52100000	[6;5;3;0;0;0]
603,616	56100000	[6;6;1;0;0;0]
603,616	45900000	[6;6;2;0;1;0]
603,616	44800000	[6;9;0;0;1;0]
603,616	60400000	[7;10;0;0;0;0]
603,616	1.12E+08	[7;5;1;0;1;0]
603,616	59900000	[7;5;2;0;1;0]
603,616	1.23E+08	[7;6;0;0;1;0]
603,616	29700000	[7;6;1;0;0;0]
603,616	19800000	[7;7;3;0;1;0]
603,616	70900000	[7;8;0;0;0;0]
603,616	24100000	[8;6;1;0;1;0]
603,616	34900000	[9;6;0;0;0;0]

Zhang et al Byonic Search

Site	AUC	Glycan
657	1.32E+08	[0;0;0;0;0;0]
657	3930000	[3;2;0;0;0;0]
657	9390000	[3;4;0;0;1;0]
657	69700000	[3;5;0;0;1;0]
657	2.75E+08	[3;6;0;0;1;0]
657	1.20E+09	[3;6;1;0;1;0]
657	26600000	[3;6;2;0;1;0]
657	66200000	[3;7;0;0;1;0]
657	8450000	[3;9;0;0;1;0]
657	6080000	[4;4;0;0;1;0]
657	4.21E+08	[4;4;1;0;0;0]
657	1.30E+08	[4;4;1;0;1;0]
657	1.09E+08	[4;5;0;0;1;0]
657	20600000	[4;5;1;0;0;0]
657	1.05E+08	[4;5;1;0;1;0]
657	1.34E+08	[4;5;2;0;0;0]
657	14300000	[4;5;2;0;1;0]

657	29300000	[4;6;0;0;1;0]
657	8.13E+08	[4;6;1;0;0;0]
657	7710000	[5;2;0;0;0;0]
657	12500000	[5;4;0;0;1;0]
657	11800000	[5;4;1;0;0;0]
657	2.19E+08	[5;4;1;0;1;0]
657	1.62E+08	[5;4;2;0;0;0]
657	1.16E+08	[5;4;2;0;1;0]
657	2.78E+08	[5;5;0;0;1;0]
657	8.23E+08	[5;5;1;0;0;0]
657	1.73E+08	[5;5;1;0;1;0]
657	3.44E+08	[5;5;2;0;0;0]
657	1.34E+08	[5;5;2;0;1;0]
657	2.66E+08	[5;6;0;0;1;0]
657	1.71E+08	[5;6;1;0;1;0]
657	3.96E+08	[5;6;2;0;1;0]
657	63900000	[5;6;3;0;1;0]
657	5770000	[6;2;0;0;0;0]
657	90100000	[6;4;1;0;0;0]
657	1.32E+08	[6;4;1;0;1;0]
657	11600000	[6;5;0;0;1;0]
657	1.50E+08	[6;5;1;0;0;0]
657	2.91E+08	[6;5;1;0;1;0]
657	3.43E+08	[6;5;2;0;0;0]
657	2.04E+08	[6;5;2;0;1;0]
657	2.63E+08	[6;5;3;0;0;0]
657	1.74E+08	[6;5;3;0;1;0]
657	59700000	[6;6;0;0;1;0]
657	5.12E+08	[6;6;1;0;0;0]
657	2.43E+08	[6;6;1;0;1;0]
657	6.15E+08	[6;6;2;0;0;0]
657	3.99E+08	[6;6;2;0;1;0]
657	71100000	[6;6;3;0;1;0]
657	37700000	[7;10;0;0;0;0]
657	22700000	[7;5;1;0;1;0]
657	1.24E+08	[7;5;2;0;1;0]
657	1.37E+08	[7;6;0;0;1;0]
657	1.78E+08	[7;6;1;0;0;0]
657	2.72E+08	[7;6;1;0;1;0]

657	2.30E+08	[7;6;2;0;0;0]
657	3.86E+08	[7;6;2;0;1;0]
657	1.81E+08	[7;6;3;0;0;0]
657	1.46E+08	[7;6;3;0;1;0]
657	1.10E+08	[7;6;4;0;0;0]
657	84700000	[7;6;4;0;1;0]
657	58800000	[7;7;0;0;1;0]
657	6.24E+08	[7;7;2;0;1;0]
657	4.63E+08	[7;7;3;0;1;0]
657	2640000	[7;7;4;0;1;0]
657	45900000	[7;8;0;0;0;0]
657	87200000	[8;6;1;0;1;0]
657	15900000	[8;7;0;0;0;0]
657	1.79E+08	[8;7;1;0;1;0]
657	2.74E+08	[8;7;2;0;1;0]
657	7540000	[8;7;3;0;1;0]
657	78300000	[9;8;3;0;1;0]
657	72200000	[9;9;0;0;1;0]

Figure S5b. Zhang et al search using GlycReSoft

Site	AUC	Glycan
122	71812707.83	[3;2;0;0;0;0]
122	15369811.75	[3;2;0;0;4;1]
122	95674214.33	[3;3;0;0;0;0]
122	137100184.6	[3;3;0;0;1;0]
122	153442010	[3;3;0;0;4;1]
122	219264988.7	[3;3;1;0;4;0]
122	921172055	[3;3;1;0;5;1]
122	657802579.5	[3;4;0;0;0;0]
122	11151226.17	[3;4;0;0;0;1]
122	2293850251	[3;4;0;0;1;0]
122	26825000.33	[3;4;0;0;2;0]
122	293188777.3	[3;4;1;0;2;0]
122	21561175.83	[3;4;1;0;3;0]
122	9498340.583	[3;4;1;0;4;0]
122	537527909.9	[3;5;0;0;0;0]
122	2819548857	[3;5;0;0;1;0]
122	242517829	[3;5;0;0;2;0]
122	20129030.67	[3;5;0;0;4;0]
122	86925169.33	[3;5;1;0;1;0]
122	52905660.33	[3;6;0;0;0;0]
122	767831683.2	[3;6;0;0;1;0]
122	146921685.8	[3;6;0;0;2;0]
122	12018654	[3;6;0;0;2;2]
122	150498697.3	[3;6;1;0;1;0]
122	4501978	[3;6;1;0;2;0]
122	30802535.33	[3;7;0;0;0;0]
122	113030558.8	[3;7;0;0;1;0]
122	312739371.1	[4;2;0;0;0;0]
122	20939820.5	[4;2;0;0;1;0]
122	176573419.3	[4;2;0;0;4;1]
122	33932477.92	[4;2;0;0;5;1]
122	33932477.92	[4;2;0;0;5;2]
122	356043195.2	[4;3;0;0;0;0]
122	540257740.8	[4;3;0;0;1;0]
122	24200891.17	[4;3;0;0;2;0]
122	107403256.7	[4;3;0;0;4;1]
122	22497198.33	[4;3;1;0;0;0]
122	81143203.83	[4;3;1;0;1;0]
122	4964661.167	[4;3;1;0;2;0]
122	1466228358	[4;3;1;0;3;0]
122	13647156.33	[4;3;1;0;4;0]
122	542896283.2	[4;4;0;0;0;0]
122	1994597117	[4;4;0;0;1;0]
122	1352290859	[4;4;0;0;2;0]
122	281703664.5	[4;4;1;0;0;0]
122	897622538	[4;4;1;0;1;0]
122	2819114.667	[4;4;2;0;0;0]
122	655226939.8	[4;5;0;0;0;0]
122	3488419680	[4;5;0;0;1;0]
122	108690825	[4;5;0;0;1;2]
122	5963698463	[4;5;0;0;2;0]
122	1705363942	[4;5;0;0;3;0]
122	16133384	[4;5;0;0;4;0]
122	132003006.3	[4;5;1;0;0;0]
122	2188532241	[4;5;1;0;1;0]
122	16779864	[4;5;1;0;1;1]
122	60781865.67	[4;5;1;0;1;2]
122	1322525129	[4;5;1;0;2;0]
122	28834677.33	[4;5;1;0;3;0]
122	2365055.5	[4;5;1;0;4;0]
122	243014259	[4;5;2;0;1;0]
122	165094626.7	[4;6;0;0;0;0]
122	841853967.5	[4;6;0;0;1;0]
122	71100137.71	[4;6;0;0;2;0]
122	54642288.33	[4;6;1;0;1;0]
122	68494292.67	[4;7;0;0;1;0]
122	62838484.67	[4;7;0;0;2;0]
122	5227954.667	[4;7;0;0;3;0]
122	54455972	[4;8;1;0;2;1]
122	4938543939	[5;2;0;0;0;0]
122	4292709.667	[5;2;0;0;4;1]
122	1180460981	[5;3;0;0;0;0]
122	1369398559	[5;3;0;0;1;0]
122	219020177.8	[5;3;0;0;2;0]

122	29746688	[5;3;0;0;2;1]
122	792494271.2	[5;3;1;0;0;0]
122	1275841107	[5;3;1;0;1;0]
122	26223700.67	[5;3;1;0;5;0]
122	1602823629	[5;4;0;0;0;0]
122	6909477804	[5;4;0;0;1;0]
122	5456019841	[5;4;0;0;2;0]
122	1708329106	[5;4;0;0;3;0]
122	25092999	[5;4;0;0;4;0]
122	3436665921	[5;4;1;0;0;0]
122	372892618.3	[5;4;1;0;0;1]
122	27881538348	[5;4;1;0;1;0]
122	5012940.833	[5;4;1;0;1;2]
122	4091636448	[5;4;1;0;2;0]
122	43262087.67	[5;4;1;0;3;0]
122	498969901.3	[5;4;2;0;0;0]
122	5174332251	[5;4;2;0;1;0]
122	20448323.83	[5;4;2;0;2;0]
122	79261350.42	[5;4;2;0;3;0]
122	29678671.58	[5;4;2;0;4;0]
122	1450041246	[5;5;0;0;0;0]
122	13853036679	[5;5;0;0;1;0]
122	4010077825	[5;5;0;0;2;0]
122	9042229.333	[5;5;0;0;2;1]
122	1584265796	[5;5;0;0;3;0]
122	61038031.83	[5;5;0;0;4;0]
122	17105956.83	[5;5;0;0;5;0]
122	3557689927	[5;5;1;0;0;0]
122	8925245266	[5;5;1;0;1;0]
122	1107532330	[5;5;1;0;2;0]
122	3174396.167	[5;5;1;0;5;0]
122	27666393.83	[5;5;2;0;0;0]
122	536070758.2	[5;5;2;0;1;0]
122	722830269.8	[5;6;0;0;0;0]
122	623306041.8	[5;6;0;0;1;0]
122	711601974.4	[5;6;0;0;2;0]
122	156573666.1	[5;6;0;0;3;0]
122	3611338.333	[5;6;0;0;4;0]
122	497951852.7	[5;6;1;0;0;0]

122	443527523	[5;6;1;0;1;0]
122	194114131.8	[5;6;1;0;2;0]
122	42641774.17	[5;6;2;0;1;0]
122	49305236.33	[5;7;0;0;1;0]
122	48214135.83	[5;7;0;0;2;0]
122	2883839.667	[5;7;0;0;3;0]
122	2234975.333	[5;7;1;0;1;0]
122	2638083762	[6;2;0;0;0;0]
122	251300965.1	[6;2;0;0;0;1]
122	1837146520	[6;3;0;0;0;0]
122	49908838	[6;3;0;0;0;1]
122	987659714.8	[6;3;0;0;1;0]
122	230192816.6	[6;3;0;0;2;0]
122	1098325193	[6;3;1;0;0;0]
122	393908240	[6;3;1;0;1;0]
122	102907423	[6;4;0;0;0;0]
122	1098686798	[6;4;0;0;1;0]
122	2811711917	[6;4;0;0;2;0]
122	661863735	[6;4;0;0;3;0]
122	49410661.33	[6;4;1;0;1;0]
122	591660151.3	[6;4;1;0;2;0]
122	2842170.667	[6;4;1;0;5;0]
122	76321971.83	[6;5;0;0;0;0]
122	1534186735	[6;5;0;0;1;0]
122	3149233854	[6;5;0;0;2;0]
122	344492449.7	[6;5;0;0;3;0]
122	19561551.83	[6;5;0;0;4;0]
122	119565425	[6;5;1;0;0;0]
122	4627095605	[6;5;1;0;1;0]
122	1003701757	[6;5;1;0;2;0]
122	30342183.25	[6;5;1;0;3;0]
122	104536781.5	[6;5;1;0;5;0]
122	89335239.17	[6;5;2;0;0;0]
122	2589247516	[6;5;2;0;1;0]
122	85722738.92	[6;5;2;0;2;0]
122	1599517.958	[6;5;2;0;3;0]
122	20435472.17	[6;5;2;0;4;0]
122	4408168	[6;5;2;0;5;0]
122	185298066.8	[6;5;3;0;1;0]

122	1936586.833	[6;5;3;0;3;0]
122	159772883.4	[6;6;0;0;0;0]
122	3553941735	[6;6;0;0;1;0]
122	1676898651	[6;6;0;0;2;0]
122	645048212.4	[6;6;0;0;3;0]
122	145054251.4	[6;6;0;0;4;0]
122	181609486.3	[6;6;1;0;0;0]
122	2510835203	[6;6;1;0;1;0]
122	614922534.2	[6;6;1;0;2;0]
122	20001081.25	[6;6;1;0;3;0]
122	796317711	[6;6;2;0;1;0]
122	71617001.67	[6;7;0;0;0;0]
122	135908214	[6;7;0;0;1;0]
122	33367508.83	[6;7;0;0;2;0]
122	12734924.5	[6;7;0;0;3;0]
122	47886919.67	[6;7;1;0;0;0]
122	40255002.5	[6;7;1;0;1;0]
122	2787351.667	[6;7;1;0;2;0]
122	12407753.17	[6;8;0;0;1;0]
122	4908010	[6;8;0;0;2;0]
122	2583606015	[7;2;0;0;0;0]
122	55766681.5	[7;2;0;0;0;1]
122	24626598.5	[7;3;0;0;0;0]
122	32107660.67	[7;3;0;0;0;1]
122	32924705	[7;3;0;0;1;0]
122	16632033.67	[7;3;0;0;2;0]
122	11329245.33	[7;3;1;0;0;1]
122	31173093.67	[7;4;0;0;0;0]
122	13827801.17	[7;5;0;0;1;0]
122	981787854.8	[7;5;0;0;2;0]
122	266735000.4	[7;5;0;0;3;0]
122	538554113.3	[7;5;1;0;2;0]
122	2615963.167	[7;5;1;0;3;0]
122	245909197.8	[7;6;0;0;1;0]
122	842459576.3	[7;6;0;0;2;0]
122	178251574.4	[7;6;0;0;3;0]
122	7615606	[7;6;0;0;4;0]
122	954386465.2	[7;6;1;0;1;0]
122	327494804.9	[7;6;1;0;2;0]
122	720497792.3	[7;6;2;0;1;0]
122	15170082.92	[7;6;2;0;2;0]
122	22651638	[7;6;2;0;3;0]
122	34191351	[7;6;3;0;1;0]
122	1178760888	[7;7;0;0;1;0]
122	494136239	[7;7;0;0;2;0]
122	342614504.8	[7;7;0;0;3;0]
122	32586105.17	[7;7;0;0;4;0]
122	1845249	[7;7;0;0;5;0]
122	1029804648	[7;7;1;0;1;0]
122	274255957.2	[7;7;1;0;2;0]
122	7689430.333	[7;7;1;0;3;0]
122	140771866.5	[7;7;2;0;1;0]
122	21233347	[7;7;3;0;1;0]
122	2616508973	[8;2;0;0;0;0]
122	235646453.3	[8;6;0;0;2;0]
122	44128259.17	[8;6;0;0;3;0]
122	941979	[8;6;0;0;5;0]
122	147941819.3	[8;6;1;0;2;0]
122	65434536.92	[8;6;2;0;2;0]
122	98613633.17	[8;7;0;0;2;0]
122	12641920	[8;7;2;0;1;0]
122	7773600.083	[8;8;0;0;1;0]
122	16290763.67	[8;8;0;0;2;0]
122	9670556	[8;8;1;0;1;0]
122	83090218	[8;8;2;0;5;1]
122	2013207264	[9;2;0;0;0;0]

Zhang et al GlycReSoft Search

Site	AUC	Glycan
149	16870050.67	[5;4;1;0;1;0]

Zhang et al GlycReSoft Search

Site	AUC	Glycan
234	76981510.67	[7;5;2;0;2;0]
234	46305166.46	[7;6;4;0;1;0]
234	75148020.88	[8;6;3;0;2;0]
234	13675582.67	[9;6;2;0;3;0]

Zhang et al GlycReSoft Search

Site	AUC	Glycan
282	193798504.7	[3;4;0;0;1;0]
282	58457176.33	[3;4;1;0;2;0]

282	28846727.17	[3;4;1;0;3;0]
282	634728021.2	[3;5;0;0;1;0]
282	44414046.33	[3;5;0;0;2;0]
282	548473792	[3;6;0;0;1;0]
282	118741696	[3;6;0;0;2;0]
282	1716721.5	[3;6;0;0;2;1]
282	253479213.7	[3;6;0;0;3;0]
282	29795490.92	[3;6;1;0;2;0]
282	108770864	[3;7;0;0;1;0]
282	237170550	[4;2;0;0;5;0]
282	248251905.7	[4;4;0;0;1;0]
282	264201985.8	[4;4;0;0;2;0]
282	68272849.67	[4;4;1;0;1;0]
282	639462754.9	[4;5;0;0;1;0]
282	1188819764	[4;5;0;0;2;0]
282	333467379.8	[4;5;0;0;3;0]
282	208641691	[4;5;1;0;1;0]
282	1776433036	[4;5;1;0;2;0]
282	19467580	[4;5;2;0;1;0]
282	71602138.33	[4;7;0;0;1;0]
282	208004592	[5;2;0;0;0;0]
282	45027760	[5;3;0;0;1;0]
282	78272938	[5;4;0;0;0;0]
282	724366528.4	[5;4;0;0;1;0]
282	518074736	[5;4;0;0;2;0]
282	1691246919	[5;4;1;0;1;0]
282	478924614.7	[5;4;1;0;2;0]
282	765219643.3	[5;4;2;0;1;0]
282	1316311482	[5;5;0;0;1;0]
282	223072503	[5;5;0;0;2;0]
282	55533372.33	[5;5;0;0;3;0]
282	280494585.7	[5;5;1;0;1;0]
282	13395072.67	[5;5;1;0;2;0]
282	262850812.3	[5;6;0;0;1;0]
282	214887260.7	[5;6;0;0;2;0]
282	5552323	[5;6;0;0;5;0]
282	163690757	[5;6;1;0;1;0]
282	50949468	[6;2;0;0;0;0]
282	212625612.2	[6;2;0;0;0;1]
282	123557073.5	[6;3;0;0;0;0]
282	29412302.67	[6;3;0;0;0;1]
282	101439674.7	[6;3;0;0;1;0]
282	17423711.33	[6;3;1;0;1;0]
282	441988591.7	[6;4;0;0;2;0]
282	238810618.8	[6;4;0;0;3;0]
282	89317444	[6;4;1;0;2;0]
282	330974461.3	[6;5;0;0;1;0]
282	287640913	[6;5;0;0;2;0]
282	1824066217	[6;5;1;0;1;0]
282	281092605.8	[6;5;1;0;2;0]
282	11654973.33	[6;5;1;0;4;0]
282	368315547.3	[6;5;2;0;1;0]
282	162841565.3	[6;5;3;0;1;0]
282	482883322	[6;6;0;0;1;0]
282	46373496.67	[6;6;0;0;2;0]
282	220864014.5	[6;6;0;0;3;0]
282	236989060	[6;6;1;0;1;0]
282	40966771.33	[6;6;1;0;3;0]
282	29248340.5	[6;7;0;0;1;0]
282	74869602.67	[7;2;0;0;0;0]
282	8642822	[7;3;0;0;1;1]
282	346688890.3	[7;5;0;0;2;0]
282	120258435.7	[7;5;1;0;2;0]
282	116579156	[7;6;0;0;1;0]
282	169953772	[7;6;0;0;2;0]
282	4766380	[7;6;0;0;3;0]
282	406724243	[7;6;1;0;1;0]
282	142543078.7	[7;6;1;0;2;0]
282	553877089.3	[7;6;2;0;1;0]
282	134062672.3	[7;7;0;0;2;0]
282	45559070.17	[8;2;0;0;0;0]
282	33212469.33	[9;2;0;0;0;0]

Zhang et al GlycReSoft Search

Site	AUC	Glycan
331,343	3956872.333	[4;6;1;0;2;0]
331,343	6587707	[6;6;1;0;2;0]
331,343	174840409.3	[6;8;0;0;2;0]
331,343	12945696.67	[7;4;0;0;5;1]

331,343	3707876.333	[7;6;0;0;4;0]

Zhang et al GlycReSoft Search

Site	AUC	Glycan
657	1556082.333	[3;6;0;0;1;0]
657	125757342.8	[4;4;0;0;2;0]
657	323985134	[4;5;0;0;2;0]
657	128654434.7	[4;5;0;0;3;0]
657	193955617.3	[4;5;1;0;2;0]
657	206564166	[5;2;0;0;0;0]
657	185258037	[5;4;0;0;1;0]
657	153698356.2	[6;2;0;0;0;0]
657	46507393	[6;2;0;0;0;1]
657	76307918.67	[7;2;0;0;0;0]
657	7032722.667	[8;2;0;0;0;0]

Figure S5c. Zhang et al search using MSFragger-Glyco

Site	AUC	Glycan
122	2031584513	[0;0;0;0;0;0]
122	163525562.7	[0;1;0;0;0;0]
122	36293684	[0;1;0;0;1;0]
122	66949208	[1;2;0;0;0;0]
122	134810160	[10;9;0;0;0;0]
122	46853404	[2;2;0;0;0;0]
122	116644543	[3;2;0;0;0;0]
122	156833782.7	[3;3;0;0;0;0]
122	212255910.2	[3;3;0;0;1;0]
122	1237990866	[3;4;0;0;0;0]
122	4684420043	[3;4;0;0;1;0]
122	998415333.8	[3;5;0;0;0;0]
122	4584857121	[3;5;0;0;1;0]
122	767987550	[3;5;0;0;2;0]
122	347099872	[3;5;1;0;1;0]
122	563564731.6	[3;6;0;0;0;0]
122	1381379426	[3;6;0;0;1;0]
122	643091639.5	[3;6;0;0;2;0]
122	168367208	[3;6;1;0;1;0]
122	161516620	[3;7;0;0;0;0]
122	473574064	[3;7;0;0;1;0]
122	329422600.4	[4;2;0;0;0;0]
122	157238536	[4;2;0;0;1;0]
122	725501808.7	[4;3;0;0;0;0]
122	731740124.8	[4;3;0;0;1;0]
122	102527593.6	[4;3;0;0;2;0]
122	150198116	[4;3;1;0;0;0]
122	150210670.7	[4;3;1;0;1;0]
122	851628412.2	[4;4;0;0;0;0]
122	3150169301	[4;4;0;0;1;0]
122	2252884081	[4;4;0;0;2;0]
122	546484628.7	[4;4;1;0;0;0]
122	1620226841	[4;4;1;0;1;0]
122	705162918.2	[4;5;0;0;0;0]
122	4045677414	[4;5;0;0;1;0]
122	3497862781	[4;5;0;0;2;0]
122	337386694.9	[4;5;1;0;0;0]
122	1915561843	[4;5;1;0;1;0]
122	141507269.3	[4;5;2;0;0;0]
122	532378348	[4;5;2;0;1;0]
122	603596230.4	[4;6;0;0;0;0]
122	1327831946	[4;6;0;0;1;0]
122	401999284.8	[4;6;0;0;2;0]
122	989431295	[4;6;1;0;0;0]
122	151417964	[4;7;0;0;0;0]
122	389806032	[4;7;0;0;1;0]
122	50478726	[4;8;0;0;0;0]
122	5783348660	[5;2;0;0;0;0]
122	1816591082	[5;2;1;0;1;0]
122	2892988608	[5;3;0;0;0;0]
122	2677498756	[5;3;0;0;1;0]
122	1177783595	[5;3;1;0;0;0]
122	1723126751	[5;4;0;0;0;0]
122	3967248464	[5;4;0;0;1;0]
122	5087723919	[5;4;0;0;2;0]
122	2353621818	[5;4;1;0;0;0]
122	10782166850	[5;4;1;0;1;0]
122	2290348963	[5;4;1;0;2;0]
122	1577481143	[5;4;1;0;3;0]
122	799100249	[5;4;2;0;0;0]
122	4353051637	[5;4;2;0;1;0]
122	171479384	[5;4;2;0;2;0]
122	2027709179	[5;5;0;0;0;0]
122	7749684000	[5;5;0;0;1;0]
122	3488584941	[5;5;0;0;2;0]
122	3344921061	[5;5;0;0;3;0]
122	2951978490	[5;5;1;0;0;0]
122	4096698582	[5;5;1;0;1;0]
122	1028327830	[5;5;1;0;2;0]
122	412719920.2	[5;5;2;0;0;0]
122	464484598.8	[5;5;2;0;1;0]
122	2226054703	[5;6;0;0;0;0]

122	1035374484	[5;6;0;0;1;0]
122	1030160594	[5;6;0;0;2;0]
122	340295668	[5;6;0;0;3;0]
122	755705537.3	[5;6;1;0;1;0]
122	244357427.2	[5;6;1;0;2;0]
122	123048177.6	[5;6;2;0;1;0]
122	4261932200	[6;2;0;0;0;0]
122	617834788.8	[6;2;2;0;1;0]
122	2736968283	[6;3;0;0;0;0]
122	1058669728	[6;3;0;0;1;0]
122	1376696017	[6;3;1;0;0;0]
122	668705317.1	[6;3;1;0;1;0]
122	157743232.3	[6;4;0;0;0;0]
122	512646307.1	[6;4;0;0;1;0]
122	1919495495	[6;4;0;0;2;0]
122	111650464	[6;4;1;0;0;0]
122	482553867.6	[6;4;1;0;1;0]
122	192019534.6	[6;5;0;0;0;0]
122	1455030657	[6;5;0;0;1;0]
122	1897221157	[6;5;0;0;2;0]
122	843460849.6	[6;5;0;0;3;0]
122	164519121	[6;5;0;0;4;0]
122	1286930529	[6;5;1;0;0;0]
122	2453744772	[6;5;1;0;1;0]
122	1033937695	[6;5;1;0;2;0]
122	375239183.2	[6;5;1;0;3;0]
122	142890042.7	[6;5;1;0;4;0]
122	276291580.8	[6;5;2;0;0;0]
122	935522313	[6;5;2;0;1;0]
122	269127648	[6;5;2;0;3;0]
122	284181493.3	[6;5;3;0;1;0]
122	484074857.3	[6;6;0;0;0;0]
122	2057370796	[6;6;0;0;1;0]
122	1533152850	[6;6;0;0;2;0]
122	628036829	[6;6;1;0;0;0]
122	1730755256	[6;6;1;0;1;0]
122	435033911.9	[6;6;1;0;2;0]
122	126080190	[6;6;2;0;0;0]
122	50632956	[6;6;2;0;2;0]
122	401792654.5	[6;7;0;0;0;0]
122	338953244.6	[6;7;0;0;1;0]
122	4322905715	[7;2;0;0;0;0]
122	87158701.33	[7;4;0;0;0;0]
122	44929100	[7;4;0;0;1;0]
122	61535132	[7;4;1;0;0;0]
122	48537960	[7;5;1;0;1;0]
122	275720736	[7;6;0;0;0;0]
122	783927131.1	[7;6;0;0;2;0]
122	609719241.8	[7;6;0;0;3;0]
122	976948508.9	[7;6;1;0;1;0]
122	287282603.4	[7;6;1;0;2;0]
122	633248017.7	[7;6;1;0;3;0]
122	144946608	[7;6;2;0;0;0]
122	717684999.4	[7;6;2;0;1;0]
122	149265284	[7;7;0;0;0;0]
122	865842900	[7;7;0;0;1;0]
122	114975565.3	[7;7;3;0;1;0]
122	111211548	[7;8;0;0;0;0]
122	2430184241	[8;2;0;0;0;0]
122	336530213.3	[8;7;0;0;0;0]
122	92606970	[8;8;0;0;0;0]
122	3440192568	[9;2;0;0;0;0]
122	137256008	[9;8;0;0;0;0]
122	235461232	[9;8;0;0;1;0]
122	34469168	[9;9;0;0;1;0]

Zhang et al MSFragger-Glyco Search

Site	AUC	Glycan
149	278879552	[3;3;0;0;0;0]
149	36486952	[5;4;0;0;1;0]
149	105866173.3	[5;4;1;0;1;0]
149	223134160	[5;4;1;0;3;0]
149	12164389	[6;4;0;0;2;0]
149	102140304	[4;5;1;0;1;0]
149	46928211	[4;5;0;0;2;0]
149	21048434	[4;5;2;0;0;0]
149	45606120	[5;5;0;0;1;0]
149	519051552	[5;5;1;0;1;0]
149	121131440	[6;5;0;0;3;0]

149	171853536	[6;5;1;0;3;0]
149	74099888	[6;5;0;0;4;0]
149	33085902	[3;6;0;0;2;0]
149	117286664	[6;6;1;0;1;0]
149	237913034.7	[7;6;0;0;0;0]
149	47911312	[7;6;0;0;3;0]
149	909618430	[8;6;1;0;0;0]
149	283581536	[8;7;0;0;0;0]

Zhang et al MSFragger-Glyco Search

Site	AUC	Glycan
165	643207230	[6;2;0;0;1;0]
165	664924933.5	[4;3;0;0;0;0]
165	239303968	[4;3;1;0;0;0]
165	616145568.7	[5;3;0;0;0;0]
165	249576928	[5;3;0;0;1;0]
165	552904325	[6;3;0;0;0;0]
165	37052704	[6;3;0;0;1;0]
165	75685008	[3;4;0;0;0;0]
165	99827868	[3;4;0;0;1;0]
165	333553632	[4;4;0;0;0;0]
165	233639168	[4;4;0;0;1;0]
165	118277405.3	[4;4;1;0;1;0]
165	72750232	[4;4;0;0;2;0]
165	401209208	[5;4;0;0;0;0]
165	182434849.6	[5;4;0;0;1;0]
165	82251472	[5;4;1;0;1;0]
165	713024048	[5;4;0;0;2;0]
165	65969768	[5;4;2;0;2;0]
165	73411784	[5;4;1;0;3;0]
165	247232122.7	[3;5;0;0;0;0]
165	324416374	[3;5;0;0;1;0]
165	116676136	[3;5;1;0;1;0]
165	253209776	[3;5;0;0;2;0]
165	151938408	[4;5;0;0;0;0]
165	443143557	[4;5;0;0;1;0]
165	1122455762	[4;5;1;0;1;0]
165	745909890	[4;5;2;0;1;0]
165	221007526.7	[4;5;0;0;2;0]
165	157655584	[4;5;1;0;0;0]
165	1231072940	[5;5;0;0;0;0]
165	172493344	[5;5;0;0;2;0]
165	278281280	[5;5;0;0;3;0]
165	391274528	[5;5;2;0;0;0]
165	133488856	[6;5;0;0;3;0]
165	20160418	[9;5;0;0;1;0]
165	325880927.1	[3;6;0;0;0;0]
165	399524721.2	[3;6;0;0;1;0]
165	246464480	[3;6;1;0;1;0]
165	398927688	[3;6;0;0;2;0]
165	266694467.6	[4;6;0;0;0;0]
165	767254720.7	[4;6;0;0;1;0]
165	250977382.9	[4;6;0;0;2;0]
165	372354880	[4;6;1;0;0;0]
165	594187200	[5;6;0;0;0;0]
165	284008827	[5;6;0;0;1;0]
165	485296922	[5;6;1;0;1;0]
165	555163317.3	[5;6;2;0;1;0]
165	522273216	[5;6;3;0;1;0]
165	343745696.8	[5;6;0;0;2;0]
165	208648320	[5;6;1;0;2;0]
165	446165193.7	[5;6;0;0;3;0]
165	175606332	[6;6;0;0;2;0]
165	207512480	[6;6;1;0;2;0]
165	610947234	[6;6;1;0;0;0]
165	163490480	[7;6;1;0;3;0]
165	29831632	[8;6;1;0;0;0]
165	173895825.8	[3;7;0;0;0;0]
165	102857932	[3;7;0;0;1;0]
165	206282608	[4;7;0;0;0;0]
165	188728440	[4;7;0;0;1;0]
165	325625464	[6;7;0;0;0;0]
165	273727850.3	[6;7;0;0;1;0]
165	132283616	[7;7;0;0;0;0]
165	179548192	[8;7;0;0;1;0]
165	46636896	[3;8;0;0;0;0]
165	87752720	[4;8;0;0;0;0]
165	112013696	[5;8;0;0;0;0]
165	58156232	[5;8;0;0;1;0]

165	170171224	[7;8;0;0;0;0]

Zhang et al MSFragger-Glyco Search

Site	AUC	Glycan
234	108366776	[6;2;2;0;1;0]
234	5403723	[5;5;0;0;2;0]
234	8758778	[5;5;1;0;2;0]
234	10103887	[5;5;1;0;0;0]
234	20483362	[6;5;3;0;1;0]
234	9162493.25	[6;5;2;0;3;0]
234	29582498.4	[6;6;2;0;0;0]
234	59499501.33	[6;6;3;0;0;0]
234	62730360	[7;6;2;0;1;0]
234	25078336	[7;6;4;0;0;0]
234	11469629.75	[8;7;4;0;1;0]
234	17541173.5	[8;7;1;0;0;0]

Zhang et al MSFragger-Glyco Search

Site	AUC	Glycan
282	640182678.7	[10;9;0;0;0;0]
282	206314368	[3;4;0;0;0;0]
282	1060547950	[3;4;0;0;1;0]
282	371502656	[3;5;0;0;0;0]
282	1282157818	[3;5;0;0;1;0]
282	308543840	[3;5;0;0;2;0]
282	394921581.3	[3;6;0;0;0;0]
282	766651016.8	[3;6;0;0;1;0]
282	799824190	[3;6;0;0;2;0]
282	243416102.7	[3;6;1;0;1;0]
282	476351008	[3;7;0;0;0;0]
282	442286339.3	[3;7;0;0;1;0]
282	85761931.33	[3;8;0;0;0;0]
282	67072060	[3;9;0;0;0;0]
282	107160682	[3;9;0;0;1;0]
282	150159568	[4;2;0;0;0;0]
282	117423952	[4;3;0;0;0;0]
282	160029328	[4;3;0;0;1;0]
282	428554720	[4;4;0;0;0;0]
282	611688030	[4;4;0;0;1;0]
282	640823282	[4;4;0;0;2;0]
282	279553102.7	[4;4;1;0;1;0]
282	464062080	[4;5;0;0;0;0]
282	763841822.5	[4;5;0;0;1;0]
282	1790519965	[4;5;0;0;2;0]
282	126919200	[4;5;1;0;0;0]
282	633209749.2	[4;5;1;0;1;0]
282	665001450	[4;6;0;0;0;0]
282	764417664	[4;6;0;0;1;0]
282	328244335.7	[4;6;0;0;2;0]
282	156403334.7	[4;6;1;0;0;0]
282	224340592	[4;7;0;0;0;0]
282	189467541	[4;7;0;0;1;0]
282	823613345	[5;2;0;0;0;0]
282	103719128	[5;2;1;0;1;0]
282	288528640	[5;3;0;0;0;0]
282	314667909.3	[5;3;0;0;1;0]
282	103591624	[5;3;1;0;0;0]
282	547519174.8	[5;4;0;0;0;0]
282	1893766358	[5;4;0;0;1;0]
282	837903525.5	[5;4;0;0;2;0]
282	302974802.7	[5;4;1;0;0;0]
282	1571215986	[5;4;1;0;1;0]
282	482509610.7	[5;4;1;0;2;0]
282	348293836	[5;4;1;0;3;0]
282	684877600	[5;4;2;0;1;0]
282	714514532.6	[5;5;0;0;0;0]
282	1276051397	[5;5;0;0;1;0]
282	880971418.6	[5;5;0;0;2;0]
282	439949762.5	[5;5;0;0;3;0]
282	414562236	[5;5;1;0;0;0]
282	1318582708	[5;5;1;0;1;0]
282	92646189.6	[5;5;1;0;2;0]
282	208999616	[5;5;2;0;0;0]
282	65670720	[5;5;2;0;1;0]
282	361636991.1	[5;6;0;0;0;0]
282	324408724.2	[5;6;0;0;1;0]
282	385603244.2	[5;6;0;0;2;0]
282	183159576	[5;6;0;0;3;0]
282	752670803.3	[5;6;1;0;1;0]
282	299791276.7	[5;6;1;0;2;0]

282	101842696	[5;6;2;0;1;0]
282	65832192	[5;8;0;0;1;0]
282	518693824	[6;2;0;0;0;0]
282	767772605	[6;2;2;0;1;0]
282	416460450.8	[6;3;0;0;0;0]
282	343514264	[6;3;0;0;1;0]
282	131164144	[6;3;1;0;0;0]
282	137164016	[6;3;1;0;1;0]
282	147588400	[6;4;0;0;0;0]
282	437782458.6	[6;4;0;0;2;0]
282	120654760	[6;4;1;0;0;0]
282	121741200	[6;5;0;0;0;0]
282	481460014.2	[6;5;0;0;1;0]
282	381581328.7	[6;5;0;0;2;0]
282	314715378.9	[6;5;0;0;3;0]
282	304318340.3	[6;5;0;0;4;0]
282	1367964363	[6;5;1;0;1;0]
282	288827321	[6;5;1;0;2;0]
282	554634752.5	[6;5;1;0;3;0]
282	364963408	[6;5;1;0;4;0]
282	932309651.5	[6;5;2;0;1;0]
282	476518351	[6;5;2;0;3;0]
282	915027923.3	[6;5;3;0;1;0]
282	375571424	[6;6;0;0;0;0]
282	381166577.7	[6;6;0;0;1;0]
282	579034602	[6;6;0;0;2;0]
282	451217299.6	[6;6;1;0;0;0]
282	1061397296	[6;6;1;0;1;0]
282	332163588	[6;6;1;0;2;0]
282	432577424	[6;6;2;0;0;0]
282	149788720	[6;6;2;0;2;0]
282	120580424	[6;6;3;0;0;0]
282	156089892	[6;7;0;0;0;0]
282	184525848	[6;7;0;0;1;0]
282	369657616	[7;2;0;0;0;0]
282	111821712	[7;5;1;0;1;0]
282	143541196	[7;6;0;0;0;0]
282	707613733.6	[7;6;0;0;2;0]
282	349531068.6	[7;6;0;0;3;0]
282	1627301953	[7;6;1;0;1;0]
282	439382806.3	[7;6;1;0;2;0]
282	317113904	[7;6;1;0;3;0]
282	2363966850	[7;6;2;0;1;0]
282	125263390.7	[7;7;0;0;0;0]
282	403731507.6	[7;7;0;0;1;0]
282	335188516	[7;7;3;0;1;0]
282	126339736	[7;8;0;0;0;0]
282	436288944	[8;2;0;0;0;0]
282	60747621.5	[8;6;1;0;0;0]
282	227812384	[8;7;0;0;0;0]
282	286929352	[8;7;0;0;1;0]
282	195865632	[8;7;1;0;0;0]
282	144816645.8	[8;7;1;0;1;0]
282	114512472	[8;8;0;0;0;0]
282	348256192	[9;2;0;0;0;0]
282	199515116	[9;8;0;0;0;0]
282	129579746.7	[9;8;0;0;1;0]
282	194371020	[9;9;0;0;1;0]

Zhang et al MSFragger-Glyco Search

Site	AUC	Glycan
657	3295621566	[0;0;0;0;0;0]
657	28220552	[0;2;0;0;1;0]
657	118223984	[1;2;0;0;1;0]
657	131718368	[10;9;0;0;0;0]
657	228074656	[12;2;0;0;0;0]
657	109441216	[3;2;0;0;1;0]
657	30617160	[3;3;0;0;0;0]
657	40767664	[3;3;0;0;1;0]
657	65705728	[3;4;0;0;0;0]
657	264440352	[3;4;0;0;1;0]
657	135999088	[3;5;0;0;0;0]
657	440289896.5	[3;5;0;0;1;0]
657	137048736	[3;6;0;0;0;0]
657	404207806.5	[3;6;0;0;1;0]
657	635428800	[3;6;0;0;2;0]
657	507302334	[3;6;1;0;1;0]
657	138621232	[3;7;0;0;0;0]
657	253393734.4	[3;7;0;0;1;0]

657	29963040	[4;3;0;0;0;0]
657	92511440	[4;4;0;0;0;0]
657	247006688	[4;4;0;0;1;0]
657	352396393.3	[4;4;0;0;2;0]
657	209051136	[4;4;1;0;0;0]
657	86433072	[4;4;1;0;1;0]
657	69658648	[4;5;0;0;0;0]
657	436154043.5	[4;5;0;0;1;0]
657	1253676290	[4;5;0;0;2;0]
657	305957524	[4;5;1;0;0;0]
657	113199461.5	[4;5;1;0;1;0]
657	274024949.3	[4;5;2;0;0;0]
657	326348232	[4;6;0;0;0;0]
657	286033727.4	[4;6;0;0;1;0]
657	596469250	[4;6;0;0;2;0]
657	256847280	[4;6;1;0;0;0]
657	41149282	[4;7;0;0;0;0]
657	199693488	[4;7;0;0;1;0]
657	499432480	[5;2;0;0;0;0]
657	370973184	[5;2;2;0;1;0]
657	157412994.7	[5;3;0;0;1;0]
657	163478352	[5;4;0;0;0;0]
657	920801920	[5;4;0;0;1;0]
657	43995860	[5;4;1;0;0;0]
657	817003137.7	[5;4;1;0;1;0]
657	166554600	[5;4;1;0;2;0]
657	146382308	[5;4;1;0;3;0]
657	209710208	[5;4;2;0;1;0]
657	337612638.9	[5;5;0;0;0;0]
657	622065845.7	[5;5;0;0;1;0]
657	252860834.3	[5;5;0;0;2;0]
657	576366456.4	[5;5;0;0;3;0]
657	206740512	[5;5;1;0;0;0]
657	210691504	[5;5;2;0;0;0]
657	478890856	[5;6;0;0;0;0]
657	204452622.5	[5;6;0;0;1;0]
657	257214936.8	[5;6;0;0;2;0]
657	158831000	[5;6;0;0;3;0]
657	25194326	[5;6;1;0;1;0]

657	169033873.5	[5;6;1;0;2;0]
657	13208683	[5;6;2;0;1;0]
657	313641057	[6;2;0;0;0;0]
657	169674898	[6;2;2;0;1;0]
657	128913264	[6;3;0;0;0;0]
657	126402272	[6;3;0;0;1;0]
657	78938024	[6;3;1;0;1;0]
657	138453362	[6;4;0;0;0;0]
657	255052632	[6;4;0;0;1;0]
657	90013964	[6;4;1;0;0;0]
657	122288128	[6;4;1;0;1;0]
657	155741485	[6;5;0;0;0;0]
657	485077568	[6;5;0;0;1;0]
657	140718928	[6;5;0;0;2;0]
657	374568451.5	[6;5;0;0;3;0]
657	126946278	[6;5;0;0;4;0]
657	316136272	[6;5;1;0;3;0]
657	433485184	[6;5;2;0;1;0]
657	199012138.7	[6;5;2;0;3;0]
657	216504544	[6;5;3;0;1;0]
657	46429984	[6;5;3;0;3;0]
657	128037688	[6;6;0;0;0;0]
657	356437472	[6;6;0;0;1;0]
657	133600536	[6;6;1;0;1;0]
657	137307744	[6;6;1;0;2;0]
657	158703008	[6;6;2;0;0;0]
657	126279472	[6;7;0;0;0;0]
657	80903430	[6;7;0;0;1;0]
657	79386272	[6;9;0;0;0;0]
657	201553128.7	[7;2;0;0;0;0]
657	110427888	[7;2;0;0;1;0]
657	40771596	[7;4;0;0;0;0]
657	61735286	[7;4;0;0;1;0]
657	63152040	[7;6;0;0;2;0]
657	208356368	[7;6;0;0;3;0]
657	27771248	[7;6;1;0;0;0]
657	931322175	[7;6;1;0;3;0]
657	530497954	[7;6;2;0;1;0]
657	59115392	[7;6;4;0;0;0]

657	69336496	[7;7;0;0;0;0]
657	137953376	[7;7;0;0;1;0]
657	91530078	[7;7;3;0;1;0]
657	103782542.4	[7;8;0;0;0;0]
657	166819848	[8;2;0;0;0;0]
657	96694896	[8;6;1;0;0;0]
657	144292416	[8;6;1;0;1;0]
657	22542840	[8;7;0;0;0;0]
657	81592304	[8;7;0;0;1;0]
657	89188888	[8;7;1;0;0;0]
657	73857760	[8;7;1;0;1;0]
657	36790068	[8;7;4;0;1;0]
657	142081926	[8;8;0;0;0;0]
657	13486832	[9;2;0;0;0;0]
657	291851904	[9;9;0;0;1;0]

Figure S5d. Zhang et al search using pGlyco2
Count data is put in place of abundance as pGlyco2 has no native quantitation

Site	Count	Glycans
17	1	[5;4;1;0;1;0]

Zhang et al pGlyco2 Search

Site	Count	Glycans
122	2	[0;1;0;0;1;0]
122	2	[0;1;0;0;1;0]
122	3	[0;2;0;0;0;0]
122	3	[0;2;0;0;0;0]
122	3	[0;2;0;0;0;0]
122	2	[1;2;0;0;0;0]
122	2	[1;2;0;0;0;0]
122	1	[10;2;0;0;0;0]
122	1	[10;6;0;0;3;0]
122	1	[10;6;1;0;1;0]
122	2	[3;7;0;0;1;0]
122	2	[3;7;0;0;1;0]
122	1	[3;7;0;0;2;0]
122	1	[4;3;0;0;0;0]
122	1	[4;3;0;0;1;0]
122	1	[4;3;0;0;2;0]
122	1	[4;3;1;0;0;0]
122	1	[4;4;2;0;2;0]
122	1	[4;5;0;0;1;0]
122	1	[4;5;0;0;2;0]
122	1	[4;5;0;0;4;0]
122	1	[4;5;1;0;0;0]
122	1	[4;5;1;0;2;0]
122	1	[4;6;0;0;4;0]
122	2	[4;6;1;0;1;0]
122	2	[4;6;1;0;1;0]
122	1	[5;2;0;0;0;0]
122	1	[5;4;0;0;0;0]
122	2	[5;4;0;0;1;0]
122	2	[5;4;0;0;1;0]
122	1	[5;4;0;0;2;0]
122	1	[5;4;0;0;3;0]
122	2	[5;4;1;0;0;0]
122	2	[5;4;1;0;0;0]
122	4	[5;4;1;0;1;0]
122	4	[5;4;1;0;1;0]
122	4	[5;4;1;0;1;0]
122	4	[5;4;1;0;1;0]
122	1	[5;4;1;0;2;0]
122	1	[5;4;1;0;4;0]
122	1	[5;4;2;0;1;0]
122	3	[5;5;0;0;0;0]
122	3	[5;5;0;0;0;0]
122	3	[5;5;0;0;0;0]
122	3	[5;5;0;0;1;0]
122	3	[5;5;0;0;1;0]
122	3	[5;5;0;0;1;0]
122	1	[5;5;0;0;2;0]
122	2	[5;5;0;0;3;0]
122	2	[5;5;0;0;3;0]
122	2	[5;5;0;0;4;0]
122	2	[5;5;0;0;4;0]
122	1	[5;5;1;0;0;0]
122	5	[5;5;1;0;1;0]
122	5	[5;5;1;0;1;0]
122	5	[5;5;1;0;1;0]
122	5	[5;5;1;0;1;0]
122	5	[5;5;1;0;1;0]
122	1	[5;5;1;0;2;0]
122	1	[5;5;2;0;0;0]
122	1	[5;5;2;0;1;0]
122	1	[5;5;2;0;4;0]
122	1	[5;6;0;0;0;0]
122	1	[5;6;0;0;1;0]
122	2	[5;6;0;0;2;0]
122	2	[5;6;0;0;2;0]
122	1	[5;6;0;0;3;0]
122	1	[5;6;0;0;5;0]
122	1	[5;6;1;0;1;0]
122	1	[5;6;1;0;2;0]

122	1	[5;6;1;0;3;0]
122	1	[5;6;2;0;1;0]
122	1	[5;6;2;0;2;0]
122	1	[5;7;0;0;1;0]
122	2	[5;7;0;0;2;0]
122	2	[5;7;0;0;2;0]
122	1	[6;4;0;0;0;0]
122	1	[6;4;0;0;1;0]
122	1	[6;4;1;0;1;0]
122	1	[6;4;1;0;2;0]
122	1	[6;4;2;0;0;0]
122	2	[6;5;0;0;0;0]
122	2	[6;5;0;0;0;0]
122	1	[6;5;0;0;1;0]
122	1	[6;5;0;0;2;0]
122	1	[6;5;0;0;4;0]
122	1	[6;5;1;0;1;0]
122	1	[6;5;1;0;2;0]
122	3	[6;5;2;0;2;0]
122	3	[6;5;2;0;2;0]
122	3	[6;5;2;0;2;0]
122	1	[6;5;3;0;1;0]
122	1	[6;6;0;0;1;0]
122	2	[6;6;0;0;4;0]
122	2	[6;6;0;0;4;0]
122	5	[6;6;1;0;0;0]
122	5	[6;6;1;0;0;0]
122	5	[6;6;1;0;0;0]
122	5	[6;6;1;0;0;0]
122	5	[6;6;1;0;0;0]
122	4	[6;6;1;0;3;0]
122	4	[6;6;1;0;3;0]
122	4	[6;6;1;0;3;0]
122	4	[6;6;1;0;3;0]
122	2	[6;6;1;0;4;0]
122	2	[6;6;1;0;4;0]
122	1	[6;6;2;0;0;0]
122	1	[6;6;2;0;2;0]
122	2	[6;7;0;0;0;0]
122	2	[6;7;0;0;0;0]
122	1	[6;7;0;0;1;0]
122	2	[6;7;0;0;2;0]
122	2	[6;7;0;0;2;0]
122	1	[6;7;0;0;3;0]
122	1	[6;7;0;0;4;0]
122	1	[6;7;1;0;0;0]
122	3	[6;7;1;0;1;0]
122	3	[6;7;1;0;1;0]
122	3	[6;7;1;0;1;0]
122	2	[6;7;1;0;2;0]
122	2	[6;7;1;0;2;0]
122	1	[7;2;0;0;1;0]
122	1	[7;5;1;0;1;0]
122	3	[7;5;1;0;2;0]
122	3	[7;5;1;0;2;0]
122	3	[7;5;1;0;2;0]
122	3	[7;5;1;0;3;0]
122	3	[7;5;1;0;3;0]
122	3	[7;5;1;0;3;0]
122	1	[7;6;0;0;0;0]
122	1	[7;6;1;0;1;0]
122	2	[7;6;1;0;2;0]
122	2	[7;6;1;0;2;0]
122	4	[7;6;1;0;3;0]
122	4	[7;6;1;0;3;0]
122	4	[7;6;1;0;3;0]
122	4	[7;6;1;0;3;0]
122	1	[7;6;2;0;3;0]
122	1	[7;6;3;0;1;0]
122	1	[7;6;3;0;2;0]
122	2	[7;7;0;0;2;0]
122	2	[7;7;0;0;2;0]
122	1	[7;7;0;0;3;0]
122	1	[7;7;0;0;4;0]
122	1	[7;7;1;0;0;0]
122	2	[7;7;1;0;2;0]
122	2	[7;7;1;0;2;0]
122	1	[7;7;1;0;3;0]

122	1	[7;7;2;0;0;0]
122	1	[7;7;2;0;1;0]
122	1	[7;7;2;0;2;0]
122	2	[7;8;0;0;0;0]
122	2	[7;8;0;0;0;0]
122	4	[8;6;1;0;2;0]
122	4	[8;6;1;0;2;0]
122	4	[8;6;1;0;2;0]
122	4	[8;6;1;0;2;0]
122	1	[8;7;0;0;0;0]
122	1	[8;7;0;0;1;0]
122	1	[8;7;0;0;2;0]
122	2	[8;7;0;0;3;0]
122	2	[8;7;0;0;3;0]
122	1	[8;7;1;0;1;0]
122	1	[8;7;1;0;2;0]
122	1	[8;7;2;0;2;0]
122	1	[8;8;0;0;1;0]
122	1	[9;6;1;0;3;0]
122	1	[9;6;2;0;0;0]

Zhang et al pGlyco2 Search

Site	Count	Glycans
149	1	[3;3;1;0;0;0]
149	1	[3;6;0;0;2;0]
149	1	[4;5;0;0;0;0]
149	1	[4;5;1;0;2;0]
149	1	[5;3;1;0;0;0]
149	1	[5;4;0;0;0;0]
149	1	[5;4;0;0;2;0]
149	2	[5;4;1;0;1;0]
149	2	[5;4;1;0;1;0]
149	5	[5;4;1;0;3;0]
149	5	[5;4;1;0;3;0]
149	5	[5;4;1;0;3;0]
149	5	[5;4;1;0;3;0]
149	5	[5;4;1;0;3;0]
149	1	[5;5;0;0;1;0]

Zhang et al pGlyco2 Search

Site	Count	Glycans
164	1	[6;5;2;0;3;0]
164	1	[6;5;3;0;1;0]

Zhang et al pGlyco2 Search

Site	Count	Glycans
234	4	[6;5;3;0;1;0]
234	4	[6;5;3;0;1;0]
234	4	[6;5;3;0;1;0]
234	4	[6;5;3;0;1;0]
234	1	[7;6;4;0;0;0]
234	1	[7;6;4;0;1;0]
234	3	[8;6;3;0;2;0]
234	3	[8;6;3;0;2;0]
234	3	[8;6;3;0;2;0]
234	4	[8;7;4;0;1;0]
234	4	[8;7;4;0;1;0]
234	4	[8;7;4;0;1;0]
234	4	[8;7;4;0;1;0]
234	1	[9;6;3;0;1;0]

Zhang et al pGlyco2 Search

Site	Count	Glycans
282	1	[10;6;0;0;2;0]
282	1	[10;7;1;0;1;0]
282	2	[3;6;0;0;1;0]
282	2	[3;6;0;0;1;0]
282	1	[4;4;0;0;2;0]
282	3	[4;5;1;0;0;0]
282	3	[4;5;1;0;0;0]
282	3	[4;5;1;0;0;0]
282	1	[4;5;1;0;1;0]
282	1	[4;5;2;0;3;0]
282	1	[4;6;0;0;1;0]
282	1	[4;6;0;0;2;0]
282	1	[4;6;1;0;0;0]
282	2	[4;6;1;0;1;0]
282	2	[4;6;1;0;1;0]
282	2	[4;7;0;0;1;0]
282	2	[4;7;0;0;1;0]
282	1	[4;7;1;0;1;0]
282	1	[4;7;2;0;0;0]

282	1	[5;2;0;0;0;0]
282	1	[5;4;0;0;2;0]
282	1	[5;4;1;0;0;0]
282	1	[5;4;1;0;1;0]
282	1	[5;4;1;0;2;0]
282	1	[5;5;0;0;0;0]
282	1	[5;5;0;0;1;0]
282	1	[5;5;0;0;3;0]
282	6	[5;5;1;0;0;0]
282	6	[5;5;1;0;0;0]
282	6	[5;5;1;0;0;0]
282	6	[5;5;1;0;0;0]
282	6	[5;5;1;0;0;0]
282	6	[5;5;1;0;0;0]
282	1	[5;5;1;0;2;0]
282	1	[5;5;2;0;0;0]
282	2	[5;5;2;0;1;0]
282	2	[5;5;2;0;1;0]
282	1	[5;5;2;0;4;0]
282	1	[5;6;1;0;1;0]
282	2	[5;6;1;0;2;0]
282	2	[5;6;1;0;2;0]
282	1	[5;6;1;0;3;0]
282	1	[5;6;2;0;1;0]
282	1	[5;6;2;0;2;0]
282	1	[5;7;0;0;0;0]
282	1	[5;7;0;0;1;0]
282	1	[5;7;0;0;3;0]
282	1	[6;2;0;0;0;0]
282	2	[6;3;0;0;0;0]
282	2	[6;3;0;0;0;0]
282	1	[6;4;1;0;2;0]
282	3	[6;5;0;0;1;0]
282	3	[6;5;0;0;1;0]
282	3	[6;5;0;0;1;0]
282	1	[6;5;0;0;3;0]
282	1	[6;5;1;0;1;0]
282	6	[6;5;1;0;2;0]
282	6	[6;5;1;0;2;0]

282	6	[6;5;1;0;2;0]
282	6	[6;5;1;0;2;0]
282	6	[6;5;1;0;2;0]
282	6	[6;5;1;0;2;0]
282	4	[6;5;2;0;1;0]
282	4	[6;5;2;0;1;0]
282	4	[6;5;2;0;1;0]
282	4	[6;5;2;0;1;0]
282	1	[6;5;3;0;1;0]
282	1	[6;6;0;0;0;0]
282	1	[6;6;0;0;1;0]
282	2	[6;6;0;0;2;0]
282	2	[6;6;0;0;2;0]
282	3	[6;6;0;0;3;0]
282	3	[6;6;0;0;3;0]
282	3	[6;6;0;0;3;0]
282	4	[6;6;1;0;0;0]
282	4	[6;6;1;0;0;0]
282	4	[6;6;1;0;0;0]
282	4	[6;6;1;0;0;0]
282	1	[6;6;1;0;1;0]
282	4	[6;6;1;0;2;0]
282	4	[6;6;1;0;2;0]
282	4	[6;6;1;0;2;0]
282	4	[6;6;1;0;2;0]
282	1	[6;6;2;0;0;0]
282	1	[6;6;2;0;1;0]
282	1	[6;6;2;0;2;0]
282	2	[6;6;3;0;0;0]
282	2	[6;6;3;0;0;0]
282	1	[6;6;4;0;0;0]
282	1	[6;7;0;0;1;0]
282	1	[6;7;0;0;2;0]
282	1	[6;7;0;0;4;0]
282	2	[6;7;1;0;0;0]
282	2	[6;7;1;0;0;0]
282	2	[6;7;1;0;1;0]
282	2	[6;7;1;0;1;0]
282	2	[6;7;1;0;2;0]

282	2	[6;7;1;0;2;0]
282	1	[6;7;1;0;3;0]
282	1	[6;7;2;0;0;0]
282	2	[6;7;2;0;1;0]
282	2	[6;7;2;0;1;0]
282	1	[6;7;2;0;2;0]
282	2	[6;7;3;0;1;0]
282	2	[6;7;3;0;1;0]
282	1	[6;8;0;0;1;0]
282	1	[7;5;1;0;1;0]
282	2	[7;5;1;0;2;0]
282	2	[7;5;1;0;2;0]
282	2	[7;6;0;0;1;0]
282	2	[7;6;0;0;1;0]
282	1	[7;6;0;0;2;0]
282	4	[7;6;1;0;1;0]
282	4	[7;6;1;0;1;0]
282	4	[7;6;1;0;1;0]
282	4	[7;6;1;0;1;0]
282	1	[7;6;1;0;2;0]
282	1	[7;6;1;0;4;0]
282	3	[7;6;2;0;1;0]
282	3	[7;6;2;0;1;0]
282	3	[7;6;2;0;1;0]
282	2	[7;6;2;0;2;0]
282	2	[7;6;2;0;2;0]
282	1	[7;6;3;0;0;0]
282	3	[7;6;3;0;1;0]
282	3	[7;6;3;0;1;0]
282	3	[7;6;3;0;1;0]
282	1	[7;6;3;0;2;0]
282	1	[7;6;3;0;3;0]
282	1	[7;6;4;0;1;0]
282	1	[7;7;0;0;1;0]
282	2	[7;7;0;0;2;0]
282	2	[7;7;0;0;2;0]
282	1	[7;7;1;0;0;0]
282	2	[7;7;1;0;1;0]
282	2	[7;7;1;0;1;0]

282	3	[7;7;1;0;2;0]
282	3	[7;7;1;0;2;0]
282	3	[7;7;1;0;2;0]
282	1	[7;7;1;0;3;0]
282	1	[7;7;2;0;0;0]
282	4	[7;7;2;0;1;0]
282	4	[7;7;2;0;1;0]
282	4	[7;7;2;0;1;0]
282	4	[7;7;2;0;1;0]
282	1	[7;7;2;0;2;0]
282	1	[7;7;2;0;3;0]
282	2	[7;7;3;0;1;0]
282	2	[7;7;3;0;1;0]
282	1	[8;6;0;0;0;0]
282	1	[8;6;0;0;1;0]
282	2	[8;6;1;0;0;0]
282	2	[8;6;1;0;0;0]
282	1	[8;6;1;0;2;0]
282	1	[8;6;1;0;3;0]
282	2	[8;6;2;0;2;0]
282	2	[8;6;2;0;2;0]
282	1	[8;6;2;0;3;0]
282	1	[8;6;3;0;0;0]
282	2	[8;6;3;0;2;0]
282	2	[8;6;3;0;2;0]
282	1	[8;7;0;0;2;0]
282	4	[8;7;1;0;1;0]
282	4	[8;7;1;0;1;0]
282	4	[8;7;1;0;1;0]
282	4	[8;7;1;0;1;0]
282	1	[8;7;1;0;2;0]
282	1	[8;7;1;0;3;0]
282	1	[8;7;2;0;0;0]
282	1	[8;8;2;0;1;0]
282	1	[9;6;1;0;1;0]
282	1	[9;6;1;0;2;0]
282	2	[9;6;1;0;3;0]
282	2	[9;6;1;0;3;0]
282	1	[9;6;1;0;4;0]

282	1	[9;7;1;0;1;0]
282	1	[9;7;1;0;2;0]
282	1	[9;7;2;0;2;0]

Zhang et al pGlyco2 Search

Site	Count	Glycans
331	1	[10;7;0;0;1;0]

Zhang et al pGlyco2 Search

Site	Count	Glycans
343	2	[10;6;1;0;1;0]
343	2	[10;6;1;0;1;0]
343	1	[10;7;0;0;1;0]
343	1	[10;7;1;0;1;0]
343	1	[10;8;0;0;1;0]
343	1	[10;8;0;0;2;0]
343	1	[10;8;0;0;4;0]
343	1	[11;7;0;0;0;0]
343	1	[11;7;0;0;1;0]
343	2	[11;8;0;0;0;0]
343	2	[11;8;0;0;0;0]
343	1	[11;8;0;0;1;0]
343	1	[11;8;0;0;2;0]
343	1	[4;7;2;0;1;0]
343	1	[5;6;1;0;2;0]
343	1	[5;6;2;0;1;0]
343	1	[7;10;0;0;1;0]
343	1	[8;6;0;0;4;0]
343	1	[8;6;2;0;1;0]
343	1	[8;8;0;0;1;0]
343	1	[8;8;0;0;2;0]
343	1	[8;8;1;0;1;0]
343	1	[9;6;2;0;0;0]

Zhang et al pGlyco2 Search

Site	Count	Glycans
657	1	[3;4;0;0;0;0]
657	3	[3;4;0;0;1;0]
657	3	[3;4;0;0;1;0]
657	3	[3;4;0;0;1;0]
657	1	[3;4;0;0;2;0]
657	1	[3;6;0;0;1;0]
657	2	[3;6;0;0;3;0]
657	2	[3;6;0;0;3;0]
657	1	[4;4;0;0;1;0]
657	1	[4;4;0;0;2;0]
657	1	[4;4;1;0;1;0]
657	2	[4;5;0;0;1;0]
657	2	[4;5;0;0;1;0]
657	3	[4;5;0;0;3;0]
657	3	[4;5;0;0;3;0]
657	3	[4;5;0;0;3;0]
657	2	[4;5;1;0;2;0]
657	2	[4;5;1;0;2;0]
657	2	[5;3;0;0;1;0]
657	2	[5;3;0;0;1;0]
657	2	[5;4;0;0;0;0]
657	2	[5;4;0;0;0;0]
657	1	[5;4;0;0;1;0]
657	2	[5;4;0;0;2;0]
657	2	[5;4;0;0;2;0]
657	1	[5;4;1;0;0;0]
657	1	[5;4;1;0;1;0]
657	2	[5;4;2;0;1;0]
657	2	[5;4;2;0;1;0]
657	1	[5;5;0;0;1;0]
657	1	[5;5;0;0;2;0]
657	1	[6;3;0;0;0;0]
657	1	[6;3;0;0;1;0]
657	1	[6;3;1;0;1;0]
657	1	[6;4;1;0;0;0]
657	1	[6;4;1;0;1;0]
657	1	[7;2;0;0;0;0]
